# Lipid mediators in post-mortem brain samples from patients with Alzheimer's disease: A systematic review

**DOI:** 10.1016/j.bbih.2024.100938

**Published:** 2024-12-23

**Authors:** Aidan D. Tyrrell, Giulia Cisbani, Mackenzie E. Smith, Chuck T. Chen, Yue-Tong Chen, Raphael Chouinard-Watkins, Kathryn E. Hopperton, Ameer Y. Taha, Richard P. Bazinet

**Affiliations:** a Department of Nutritional Sciences, Temerty Faculty of Medicine, University of Toronto, Canada; b Department of Food Science and Technology, College of Agriculture and Environmental Sciences, University of California, Davis, CA, USA

**Keywords:** Lipid mediators, Alzheimer's disease, Neuroinflammation, Post-mortem

## Abstract

A proposed contributor to Alzheimer's disease (AD) pathology is the induction of neuroinflammation due to tau and beta-amyloid protein accumulation causing neuronal injury and dysfunction. Dysregulation of lipid mediators derived from polyunsaturated fatty acids may contribute to this inflammatory response in the brain of patients with AD, yet the literature has not yet been systematically reviewed. A systematic search was conducted in Medline, Embase and PsychINFO for articles published up to April 22, 2024. Papers were included if they measured levels of lipid mediators and/or enzymes involved in their production in *post-mortem* brain samples from patients with AD and control without neurological disease. A total of 50 relevant studies were identified. Despite heterogeneity in the results, pro-inflammatory lipid mediators, including 5-, 11-, 12- and 15-hydroxyeicosatetraenoic acid oxylipins and prostaglandin D2, were significantly higher, while anti-inflammatory lipoxin A4 and DHA-derived docosanoids were significantly lower in brains of patients with AD compared to control (16 studies). Thirty-seven articles reported on enzymes, with 32 reporting values for enzyme level changes between AD and controls. Among the 32 articles, the majority reported on levels of cyclooxygenase (COX) (18/32), with fewer studies reporting on phospholipase (8/32), lipoxygenase (LOX) (4/32) and prostaglandin E synthase (4/32). Enzyme levels also exhibited variability in the literature, with a trend towards elevated expression of enzymes involved in the pro-inflammatory response, including COX and LOX enzymes. Overall, these results are consistent with the involvement of neuroinflammation in the pathogenesis of AD measured by lipid mediators. However, the specific contribution of each lipid metabolite and enzymes to either the progression or persistence of AD remains unclear, and more research is required.

## Highlights

• Systematic review of lipid mediators in brains of patients with Alzheimer's disease (AD).
• Dysregulation of lipid mediator levels may contribute to AD pathology.
• Pro-inflammatory lipid mediators such as prostaglandin D2 are higher in AD.
• Anti-inflammatory lipid mediators, including docosanoids, are lower in AD.
• Enzymes involved in the pro-inflammatory response are elevated in AD.

## 1 Introduction

Alzheimer's disease (AD) is a common form of dementia affecting over 50 million people worldwide, characterized by cognitive decline and memory loss, with key pathological features being amyloid plaques and neurofibrillary tangles in the brain (Breijyeh and Karaman, 2020). Despite being the primary hallmarks of AD, emerging evidence suggests that amyloid plaque and neurofibrillary tangle accumulation alone cannot explain the complete pathogenesis of AD (Leng and Edison, 2020). A large body of literature points to the contribution of neuroinflammation in AD neuropathology due to the elevated presence of inflammatory markers as well as genes involved in immune function being associated with AD risk (Leng and Edison, 2020; Hopperton et al., 2018). In the early stages, inflammation mediated by microglia clears the pathological proteins accumulating in the parenchyma, however, as inflammation becomes chronic and not resolving, it can eventually lead to neuronal damage, exacerbating disease progression (Hopperton et al., 2018; Leng and Edison, 2021; Patani et al., 2023a; Desale and Chinnathambi, 2020).

**Fig. 1:**
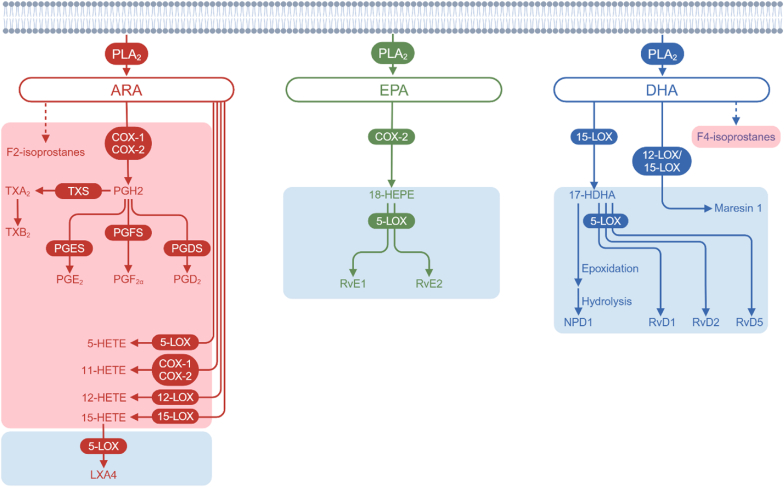
Synthesis pathways of select lipid mediators and enzymes involved in their synthesis reported in *post-mortem* human brain samples and discussed in this review. Solid arrows represent enzymatic pathways. Dashed arrows represent non-enzymatic reactions. Red background box represents pro-inflammatory lipid mediators. Blue background box represents pro-resolution lipid mediators. ARA, arachidonic acid; COX, cyclooxygenase; DHA, docosahexaenoic acid; EPA, eicosapentaenoic acid; HDHA, hydroxydocosahexaenoic acid; HEPE, hydroxyeicosapentaenoic acid; HETE, hydroxyeicosatetraenoic acids; LOX, lipoxygenase; LXA4, lipoxin A4; NPD1, neuroprotectin D1; PGDS, prostaglandin D synthase; PGD2, prostaglandin D2; PGES, prostaglandin E synthase; PGE_2_, prostaglandin E2; PGFS, prostaglandin F synthase; PGF_2α_, prostaglandin F2α; PGH2, prostaglandin H2; PLA_2_, phospholipase A_2_; RvE, E-series resolvins; RvD, D-series resolvins; TXA_2_, Thromboxane A2; TXB_2_, Thromboxane B2; TXS, thromboxane synthase.

Various pro-inflammatory mediators, including bioactive oxygenated lipids broadly termed lipid mediators or “oxylipins”, can influence microglia and astrocytes behaviour (Hopperton et al., 2018; Calsolaro and Edison, 2016). Lipid mediators are derived from polyunsaturated fatty acids (PUFA) including arachidonic acid (ARA), eicosapentaenoic acid (EPA), and docosahexaenoic acid (DHA). PUFA esterified in the phospholipid membrane are released via the action of phospholipase A_2_ (PLA_2_) to produce free fatty acids which are available for metabolism via β-oxidation or production of lipid mediators (Lacombe et al., 2018). Lipid mediators derived from ARA exert both pro-inflammatory (prostaglandins, leukotrienes, and thromboxanes), and anti-inflammatory (lipoxins and epoxides) effects (Serhan, 2014). Conversely, lipid mediators derived from DHA and EPA, categorized as specialized pro-resolving mediators (SPM), exert anti-inflammatory or pro-resolving effects (Serhan et al., 2014; Levy et al., 2001; Serhan and Levy, 2018; Bazinet and Layé, 2014). The enzymes responsible for the synthesis of lipid mediators includes lipoxygenases (LOX), cyclooxygenases (COX), and cytochrome P450 (CYP) (Hajeyah et al., 2020; Dyall et al., 2022; Christi and Harwoo, 2020) (Hajeyah et al., 2020; Dyall et al., 2022; Christi and Harwoo, 2020). PUFA including ARA, DHA and EPA may also undergo nonenzymatic, free radical peroxidation to form prostaglandin-like isoprostanes (IsoPs) upon their release from the lipid membrane (Morrow et al., 1999). Peroxidation of ARA leads to the formation of four F_2_-IsoPs regioisomers, 5-,12-,8-, and 15-series, while peroxidization of EPA and DHA produces F_3_-IsoPs and F_4_-IsoPs, respectively, which are released from the membrane by phospholipase enzymes (Geng et al., 2022) (Fig. 1).

To date, the literature has not been systematically reviewed to assess the contribution of lipid mediators, IsoPs and the enzymes involved in lipid mediator synthesis to AD and to determine whether significant changes in the brain exist. In this systematic review, we reviewed the literature to examine changes in lipid mediators and the enzymes involved in their synthesis in *post-mortem* human brain samples from patients with AD and control.

## 2 Materials & methods

### 2.1 Search strategy

The Ovid interface was used to conduct a systematic search in databases including Medline, Embase and PsychInfo for articles published up to April 22, 2024. The search protocol was developed based on Preferred Reporting Items for Systematic Reviews and Meta-Analyses (PRISMA) and World Health Organization (WHO) Review Protocol Template Guidelines where applicable for a systematic review of descriptive (non-interventional) data (Page et al., 2021).

### 2.2 Inclusion and exclusion criteria

Articles were screened using the Covidence software (https://www.covidence.org/) following eligibility criteria **(see supplementary material ‘eligibility criteria’)**. Papers published in peer-reviewed journals measuring levels of lipid mediators, IsoPs and enzymes involved in lipid mediator production in human *post-mortem* brain samples from patients with AD and controls without neurological disease were included. The search included ARA-derived eicosanoids and IsoPs, EPA-derived eicosanoids, DHA-derived docosanoids and IsoPs, endocannabinoids, cyclooxygenases, lipoxygenases, prostaglandin E synthase, and phospholipase terms with synonyms and related words as both MeSH/EMTREE terms and as keywords for title, abstract and keywords search terms specifically designed for each database. Full search terms for each database and results found per database are listed in the Supplementary material Appendix 1, 2 and 3. Conference proceedings, abstracts, reviews and animal studies were excluded. Additionally, studies were excluded if they measured lipid mediators and enzymes outside of the brain, consisted of PET studies on living subjects, or did not include a control group of patients without neurological disease. Grey literature search was conducted by reviewing the bibliographies of papers deemed eligible for this review. The initial aim of the study was to include all lipid mediators however it was decided to omit articles that measured endocannabinoids and endocannabinoid receptors to keep the discussion more focused.

### 2.3 Data extraction

Two independent reviewers screened each article (GC, MES). A third reviewer (KEH) independently assessed any conflict raised during the screening phases. Extracted data included number of cases per sex, age, brain regions examined, *post-mortem* delay (time between death and retrieval of brain), origin of brain samples, brain fixation technique, anti-inflammatory drug use at death, the methods used for identification and quantification of lipid mediators and enzymes, the results of the expression levels in AD compared to control, and the percent difference in AD compared to control (**see supplementary material ‘outcomes and prioritization’**). The terms ‘higher’ or ⇑ and ‘lower’ or ⇓ are used in the tables to show statistically significant higher or lower levels of lipid mediators and enzymes in the study relative to the neuropathologically normal patient control. ⇔ was also used to denote no statistically significant change between patients with AD and controls. Statistical differences are reported as described in the original articles. Additionally, percent difference was used to show the difference between AD compared to control which is reported in both the supplementary and main tables. Data were listed as ‘Not Reported’ if the relevant information could not be found in the article or supplementary text. The data was not comparable enough (brain region analyzed, lipid mediators measured, analytical method, participant age and sex, AD stage) to evaluate levels of lipid mediators and enzymes involved in their synthesis to perform a meta-analysis. However, we reported the percent difference between AD and control brains within each study. Data that are only available in graphical representations, was extracted using online software (https://apps.automeris.io/wpd/) which estimates the data values to then be used to calculate percentage of difference between groups.

## 3 Results

**Fig. 2:**
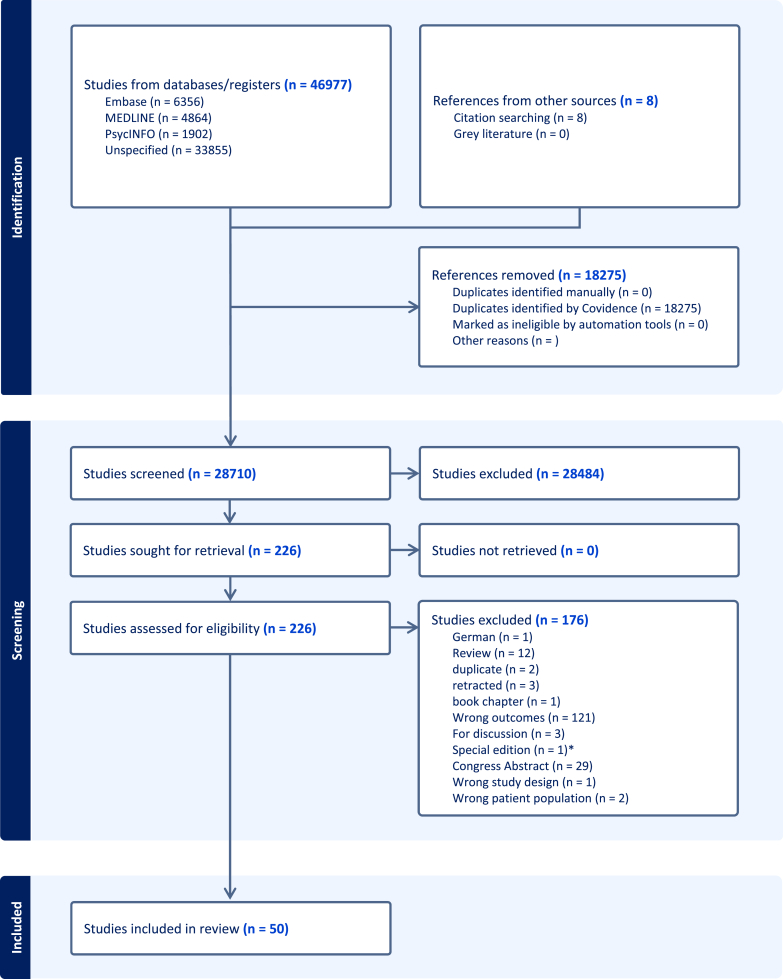
Covidence flow diagram of systematic search. ∗(Gattaz et al., 1996) was excluded as it was a special edition including results from Gattaz et al., 1995).

A total of 28 710 articles were screened, of which 226 met the inclusion criteria for full text review. A total of 50 articles, selected during the full text review screening (n = 42) and citation searching (n = 8) phases, were fully extracted, and summarized in the present review (Fig. 2). Details on subjects, diagnosis criteria of AD, *post-mortem* delays and methodologies are reported in Supplementary Tables 1–5.

### 3.1 Lipid mediators

A total of 16 of 50 articles included in this review reported on lipid mediators derived from ARA, EPA or DHA in several *post-mortem* brain regions from patients with AD and control brains assessing either prostanoid production (Wong et al., 1992), prostanoid localization by immunohistochemistry (Casadesus et al., 2007) or analyzing specific lipid mediators by gas chromatography–mass spectrometry (GC/MS), GC-MS/negative ion chemical ionization (NICI) (Praticò et al., 1998, 2004; Nourooz-Zadeh et al., 1999; Reich et al., 2001; Yao et al., 2003; Forman et al., 2007; Bhatia et al., 2013; Fessel et al., 2003), Liquid chromatography–mass spectrometry (LC/MS/MS) (Lukiw et al., 2005; Wang et al., 2015; Zhu et al., 2016; Furman et al., 2018; Kurano et al., 2022; Ebright et al., 2022), or enzyme immunoassay (Wang et al., 2015) (Supplementary Table 6).

#### 3.1.1 Prostanoids

**Fig. 3:**
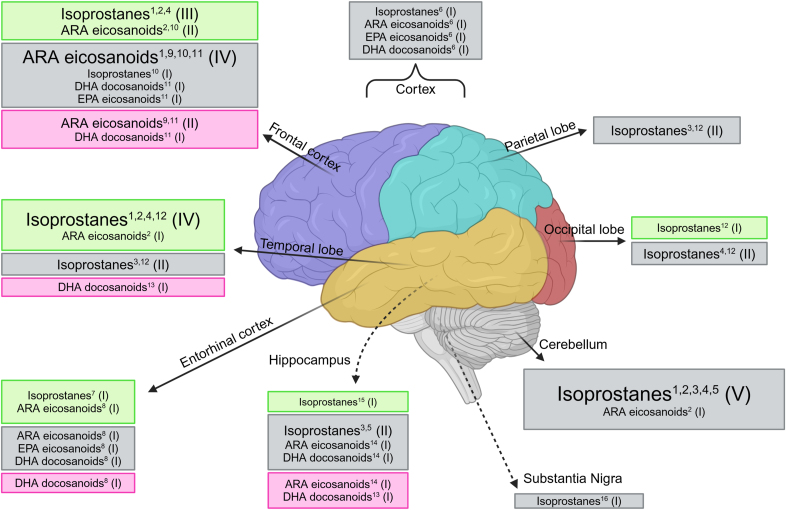
Lipid mediator summary results. Boxes represent lipid mediators where levels were reported and compared between *post-mortem* brains from patients with AD or control. Green boxes represent significant increase, grey represent no significant difference, and magenta represent significant decrease in *post-mortem* brains from patients with AD compared to control. Roman numeral in brackets represents number of studies reporting the finding, with the size of text proportional to the number of findings. References are indicated in superscript: 1, (Pratico et al., 1998); 2, (Praticò et al., 2004); 3, (Reich et al., 2001); 4, (Yao et al., 2003); 5, (Bhatia et al., 2013); 6, (Kurano et al., 2022); 7, (Forman et al., 2007); 8, (Zhu et al., 2016); 9, (Wong et al., 1992); 10, (Furman et al., 2018); 11, (Ebright et al., 2022); 12, (Nourooz-Zadeh et al., 1999); 13, (Lukiw et al., 2005); 14, (Wang et al., 2015); 15, (Casadesus et al., 2007); 16, (Fessel et al., 2003). ARA eicosanoids include all lipid mediators except for F_2_-isprostanes. Isoprostanes includes both F_2_- and F_4_-isoprostanes. DHA docosanoids includes all docosanoids reported. ARA, Arachidonic acid; DHA, Docosahexaenoic acid; EPA, Eicosapentaenoic acid.

Through the COX pathway, the metabolism of ARA leads to prostanoid production including prostaglandin and thromboxane (Malmsten, 1986). Using an assay, Wong and colleagues reported a significant reduction in prostanoid levels in *post-mortem* frontal cortex of patients with AD as compared to control (Wong et al., 1992). Prostaglandin E_2_ (PGE_2_), including prostaglandin A_2_ (PGA_2_), was the major metabolite and accounted for approximately 63% of total production through the COX pathway in this brain region (Wong et al., 1992). Prostaglandin F2_α_ (PGF_2α_), thromboxane B_2_ (TXB_2_) and prostaglandin D_2_ (PGD_2_) were relatively minor products and accounted for 21.5, 9 and 6.5%, respectively (Wong et al., 1992). Compared to control, *post-mortem* brains of patients with AD had a 46% downregulation in ARA metabolism measured by total prostanoid production (Wong et al., 1992). Overall, the production of PGE_2_ (45% reduction), PGF_2α_ (48% reduction) and PGD_2_ (63% reduction) were significantly lower in the frontal cortex of patients with AD compared to control (Wong et al., 1992). Interestingly, the use of anti-inflammatory drugs by patients’ *pre-mortem*, was associated with a significant increase in prostanoid production (Wong et al., 1992). Excluding samples from patients that used anti-inflammatory drugs, a significantly lower production of PGE_2_, PGF_2_, PGD_2_ and total prostanoid of about 66% was observed in the frontal cortex of patients with AD compared to control (Wong et al., 1992). More recently, using LC/MS/MS to determine the lipid mediator profile, PGD_2_ was significantly higher in the entorhinal cortex (Zhu et al., 2016) of patients with AD compared to control, with no changes in either the frontal cortex (Furman et al., 2018) or hippocampus (Wang et al., 2015), while PGF_2α_ was significantly lower in the dorsolateral prefrontal cortex (DLPFC) of patients with AD compared to controls (Ebright et al., 2022) (Fig. 3).

#### 3.1.2 Isoprostanes

One paper reported on the levels of iPF_2α_ -III (8-series F_2_-IsoP), iPF_2α_ -VI (5-series F_2_-IsoP) and 6-keto PGF_1α_ in the frontal and temporal cortices, and cerebellum (Praticò et al., 1998). While 6-keto PGF_1α_ (an index of prostaglandin production) did not change, iPF2_α_ -III and iPF2_α_ -VI were significantly elevated in both the frontal and temporal cortices in *post-mortem* brains of patient with AD as compared to control (Supplementary Table 6) (Praticò et al., 1998). However, the cerebellum did not have the same elevation in those lipid mediators (Praticò et al., 1998). The levels of another major F2-IsoPS, 8,12-iso-iPF_2α_ -VI, was reported in three papers which also demonstrated significantly higher levels in the frontal and temporal cortex with no differences in the occipital cortex and cerebellum of *post-mortem* brains of patients with AD as compared to control (Yao et al., 2003; Forman et al., 2007; Praticò et al., 2004). F_2_-IsoPs were significantly elevated in the hippocampus of *post-mortem* brains of patients with AD only at Braak stage III/IV compared to control, while there were no differences in the cerebellum (Bhatia et al., 2013). Microscopic localization of 8-iso-PGF_2α_ and 13,14-dihydro 15-keto PGF_2α_ by immunohistochemistry found significantly higher immunoreactivity of pyramidal neurons in the hippocampus of *post-mortem* brains of patients with AD as compared to control (Casadesus et al., 2007). Two papers reported on F_4_-IsoPs derived from DHA (Nourooz-Zadeh et al., 1999; Reich et al., 2001). F_4_-IsoPs were significantly increased in temporal and occipital cortex (Nourooz-Zadeh et al., 1999) with no change in the cortex (Reich et al., 2001), parietal cortex (Nourooz-Zadeh et al., 1999), cerebellum (Reich et al., 2001), or hippocampus (Reich et al., 2001) of patients with AD when compared to control. Interestingly in both papers F2-IsoPs did not differ between *post-mortem* brains of patients with AD as compared to control (Nourooz-Zadeh et al., 1999). Levels of ARA and DHA in the brain regions was also analyzed in the two mentioned studies, however no significant differences were also observed (Nourooz-Zadeh et al., 1999; Reich et al., 2001) (Fig. 3).

#### 3.1.3 12/15 LOX products

Five articles reported on hydroxyeicosatetraenoic acids (HETE), products of ARA oxidation (Wang et al., 2015; Zhu et al., 2016; Furman et al., 2018; Kurano et al., 2022; Praticò et al., 2004). Levels of 5-HETE, 11-HETE, 12-HETE, and 15 HETE were significantly higher by 53–57% in the frontal cortex of patients with AD (Pratico et al., 2004; Furman et al., 2018), alongside 12-HETE and 15-HETE being significantly higher (52–64%) in the temporal cortex of patients with AD (Praticò et al., 2004). In contrast, levels of 12-HETE and 15-HETE did not differ in the cerebellum (Praticò et al., 2004). Additionally, others reported no differences in any HETE lipid mediators in the hippocampus (Wang et al., 2015), cortex (Kurano et al., 2022), and entorhinal cortex (Zhu et al., 2016) (Fig. 3).

**Specialized pro-resolving mediators(SPM):** SPM include lipoxins, resolvins, protectins and maresins (Serhan, 2014). Lipoxins are derived from ARA, while resolvins, protectins and maresins are derived from omega-3 PUFAs, EPA and DHA (Serhan, 2014). Three articles reported on the levels of pro-resolving lipid mediators in *post-mortem* AD patient brains (Lukiw et al., 2005; Wang et al., 2015; Zhu et al., 2016). Neuroprotectin D1 (NPD1) was significantly lower in the DLPFC (Ebright et al., 2022), hippocampus (Lukiw et al., 2005), temporal lobe (Lukiw et al., 2005) and entorhinal cortex (Zhu et al., 2016) but not in the thalamus (Lukiw et al., 2005) and occipital lobe (Lukiw et al., 2005) of *post-mortem* brains of patients with AD as compared to control. Lipoxin A4 (LXA4) was significantly lower in the hippocampus in *post-mortem* brains of patients with AD using an enzyme immunoassay (Wang et al., 2015). However, both LXA4 and Maresin1 were not detected in *post-mortem* brains of patients with AD using LC/MS/MS (Wang et al., 2015; Zhu et al., 2016). Levels of resolvin D1 (RvD1) as well as resolvin D2 (RvD2) were similar between patients with AD and control groups (Wang et al., 2015; Zhu et al., 2016). While resolvin D5 (RvD5) levels did not change between cortex of patients with AD and control, when specific cortical regions were analyzed RvD5 was significantly lower in entorhinal cortex of patients with AD as compared to control (Zhu et al., 2016) (Fig. 3).

### 3.2 Cyclooxygenases

Eighteen articles reported values for COX-2 and COX-1 levels in *post-mortem* brains (Supplementary Table 7). The majority of the articles (17/18) reported on the levels of COX-2 (Chang et al., 1996; Lukiw and Bazan, 1997; Pasinetti and Aisen, 1998; Ho et al., 1999; Ho et al., 2001; Yermakova and O'Banion, 2001; Kitamura et al., 1999; Hoozemans et al., 2001; Hoozemans et al., 2005; Hoozemans et al., 2004; Yokota et al., 2003; Yokota et al., 2004; Mohri et al., 2007; Fujimi et al., 2007; Yasojima et al., 1999; Hoozemans et al., 2002; Colangelo et al., 2002) with 6 reporting on COX-1 levels (Lukiw and Bazan, 1997; Pasinetti and Aisen, 1998; Kitamura et al., 1999; Mohri et al., 2007; Yasojima et al., 1999; Yermakova et al., 1999) (Supplementary Table 7). COX levels were determined using three different techniques (qPCR, immunohistochemistry, and western blot) and reported as COX mRNA, immunoreactivity , protein or density.

#### 3.2.1 COX-2

**Fig. 4:**
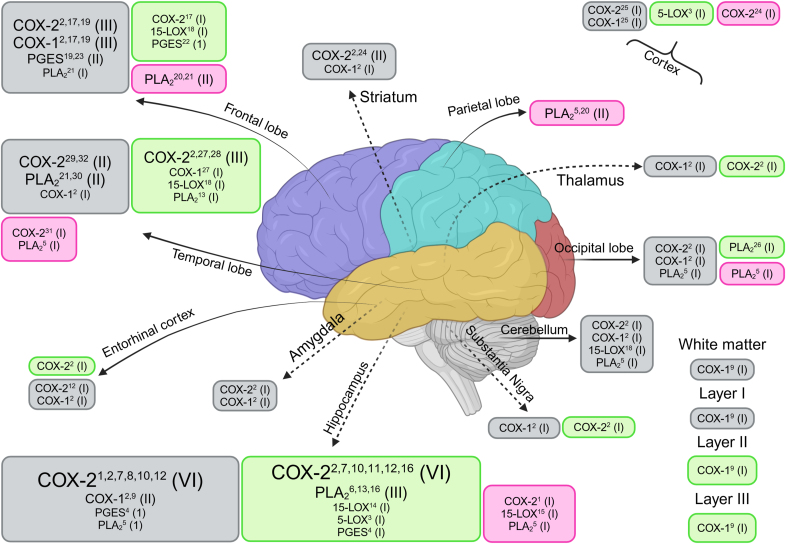
Enzyme summary results. Boxes represent enzymes where levels (mRNA, protein or density) were reported and compared between *post-mortem* brains from patients with AD or control. Green boxes represent significant increase, grey represent no significant difference, and magenta represent significant decrease in *post-mortem* brains from patients with AD compared to control. Roman numeral in brackets represents number of studies reporting the finding, with the size of text proportional to the number of findings. References are indicated in superscript: 1, (Yermakova & O’Banion, 2001); 2, (Yasojima et al., 1999); 3, (Firuzi et al., 2008); 4, (Akitake et al., 2013); 5, (Ross et al., 1998); 6, (Sanchez-Mejia et al., 2008); 7, (Ho et al., 1999); 8, (Ho et al., 2001); 9, (Yermakova et al., 1999); 10, (Yokota et al., 2003); 11, (Yokota et al., 2004); 12, (Fujimi et al., 2007); 13, (Moses et al., 2006); 14, (Wang et al., 2015); 15, (Lukiw et al., 2005); 16, (Colangelo et al., 2002); 17, (Pasinetti & Aisen, 1998); 18, (Praticò et al., 2004); 19, (Mohri et al., 2007); 20, (Gattaz et al., 1995); 21, (Talbot et al., 2000); 22, (U. A. Chaudhry et al., 2008); 23, (U. Chaudhry et al., 2010); 24, (Chang et al., 1996); 25, (Lukiw & Bazan, 1997); 26, (Stephenson et al., 1996); 27, (Kitamura et al., 1999); 28, (J. J. Hoozemans et al., 2001); 29, (J. J. M. Hoozemans et al., 2005); 30, (Kanfer et al., 1993); 31, (J. J. M. Hoozemans et al., 2004); 32, (J. J. M. Hoozemans et al., 2002). PGES includes mPGES1, mPGES2, and cPGES. PLA_2_ includes cPLA_2_ and sPLA_2_. COX, cyclooxygenase; LOX, lipoxygenase; cPGES, cytosolic prostaglandin E synthase; mPGES, membrane-associated prostaglandin E synthase; PLA_2_, phospholipase A_2_.

*COX-2* mRNA expression levels were significantly downregulated in the neocortex (Chang et al., 1996), and significantly upregulated in the hippocampus (Yasojima et al., 1999; Colangelo et al., 2002), entorhinal cortex (Yasojima et al., 1999), frontal cortex (Pasinetti and Aisen, 1998), mid temporal gyrus (Yasojima et al., 1999), substantia nigra (Yasojima et al., 1999), and thalamus (Yasojima et al., 1999) of patients with AD as compared to control. Conversely others reported no change in the neocortex (Lukiw and Bazan, 1997, 1998), frontal cortex (Mohri et al., 2007), occipital cortex (Yasojima et al., 1999), motor cortex (Yasojima et al., 1999), superior temporal gyrus (Lukiw and Bazan, 1998), amydala (Yasojima et al., 1999), striatum (Yasojima et al., 1999), and cerebellum (Yasojima et al., 1999) between patients with AD and control. At the protein level, COX-2 was significantly upregulated in the hippocampus (Ho et al., 1999; Yokota et al., 2003, 2004; Fujimi et al., 2007), including the CA1, CA2, CA3, and CA4 pyramidal layers, and temporal cortex (Kitamura et al., 1999; Hoozemans et al., 2001) in patients with AD as compared to control. Conversely, Yermakenova *et al*. following similar immunohistochemistry techniques reported significant downregulation of COX-2 protein expression in the hippocampal CA1 and CA3 regions in patients with AD as compared to control (Yermakova and O'Banion, 2001) (Fig. 4).

**Fig. 5:**
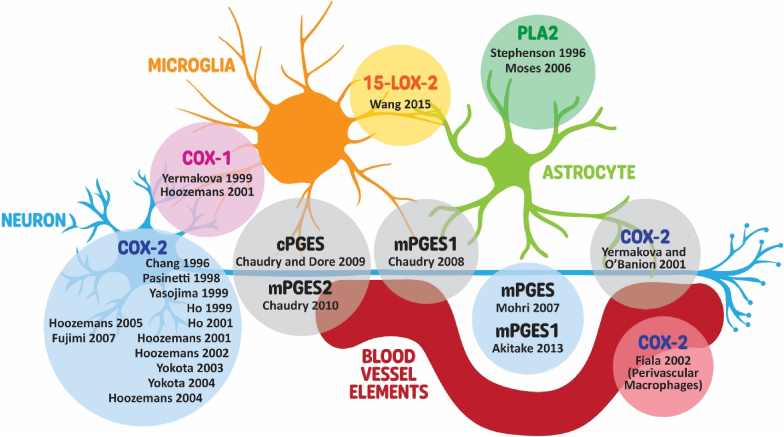
Schematic representation of enzyme localization within cellular structures in the brain. Circles denote enzymes, with each containing the names of articles reporting their localization. Contact between a circle and a cellular structure indicates reported localization of the enzyme within that structure. COX, cyclooxygenase; LOX, lipoxygenase; cPGES, cytosolic prostaglandin E synthase; mPGES, membrane-associated prostaglandin E synthase; PLA_2_, phospholipase A_2_.

When looking at the cellular localization of COX-2, expression was largely detected in neuronal perikaryons, but also in dendrites and axons in the temporal cortex (Hoozemans et al., 2001, 2002, 2004, 2005; Yasojima et al., 1999) and hippocampus (Yokota et al., 2003, 2004; Fujimi et al., 2007; Yasojima et al., 1999) as well as other brain regions (Yasojima et al., 1999). COX-2 was also expressed in perivascular macrophages (Fiala et al., 2002) but undetected in cells with glial morphology (Hoozemans et al., 2001) (Fig. 5).

#### 3.2.2 COX-1

No significant changes in mRNA levels of *COX-1* were reported (Lukiw and Bazan, 1997; Pasinetti and Aisen, 1998; Mohri et al., 2007; Yasojima et al., 1999; Yermakova et al., 1999). Conversely, COX-1 protein levels were significantly elevated in the temporal cortex of patients with AD as compared to control (Kitamura et al., 1999) (Fig. 4). Additionally, COX-1 was expressed in neurons and microglia (Hoozemans et al., 2001; Yermakova et al., 1999), but not in astrocytes (Yermakova et al., 1999) (Fig. 5).

### 3.3 Lipoxygenases

Four articles reported values for LOX enzymes levels in *post-mortem* brains (Lukiw et al., 2005; Wang et al., 2015; Praticò et al., 2004; Firuzi et al., 2008), more specifically 3 on 15-LOX (Lukiw et al., 2005; Wang et al., 2015; Praticò et al., 2004) and 1 on 5-LOX (Firuzi et al., 2008) using immunohistochemistry (Supplementary Table 7).

#### 3.3.1 15-LOX

Gene expression of *15-LOX* was significantly lower by 2-fold in the CA1 of patients with AD as compared to the control (Lukiw et al., 2005). In contrast, 15-LOX protein levels were significantly higher in the CA1 (154%) (Wang et al., 2015), the mid-frontal (60%), and mid-temporal (55%) cortices (Pratico et al., 2004), but not in the cerebellum (Pratico et al., 2004) of *post-mortem* brains of patients with AD as compared to control (Fig. 4). Additionally, immunohistochemistry staining revealed that 15-LOX was localized to the glial cells but not neurons and was most predominant in the CA2-4 and sub granular zone of the dentate gyrus (DG) regions in the hippocampus, with markedly fewer immunopositive cells in the CA1 where significant changes in 15-LOX protein level was observed (Wang et al., 2015) (Fig. 5).

#### 3.3.2 5-LOX

5-LOX was significantly upregulated in hippocampus (403% elevated) and cortex (40% elevated) of patients with AD when compared to control brains (Firuzi et al., 2008) (Fig. 4). Additionally, a robust elevation of 5-LOX in dystrophic neurons and amyloid plaques found in the medial temporal lobe of patients with AD was observed however values were not reported (Ikonomovic et al., 2008).

### 3.4 Prostaglandin E synthase

Four studies reported values for three different types of prostaglandin E synthase (PGES) levels in *post-mortem* brains: microsomal prostaglandin E synthase-1 (mPGES-1) (Mohri et al., 2007; Chaudhry et al., 2008; Akitake et al., 2013), microsomal prostaglandin E synthase-2 (mPGES-2) (Mohri et al., 2007; Akitake et al., 2013; Chaudhry et al., 2010) and cytosolic prostaglandin E2 synthase (cPGES) (Mohri et al., 2007; Chaudhry et al., 2008, 2010; Akitake et al., 2013) in *post-mortem* brains of patients with AD (Supplementary Table 7). Results of studies reporting on either three types of PGES have been summarized in Fig. 4.

#### 3.4.1 mPGES-1

*mPGES-1* mRNA levels were similar in the frontal cortex of patients with AD as compared to control brains by real-time polymerase chain reaction (RT-qPCR) (Mohri et al., 2007). Conversely, protein expression by immunohistochemistry was significantly higher in the middle frontal gyrus (MFG) (Chaudhry et al., 2008) and hippocampus (Akitake et al., 2013) of patients with AD as compared to control. mPGES-1 was mainly localized in pyramidal neuron, microglia, astrocytes, and endothelial cells but not in smooth muscle in the MFG (Chaudhry et al., 2008) (Fig. 5). On the contrary, others found mPEGS-1 positive immunostaining in neurites and cytoplasm of neurons in CA3-4; CA1 and CA4, where the DG, astrocytes, and white matter of the cortex was devoid of staining (Akitake et al., 2013) (Fig. 5).

#### 3.4.2 mPGES-2

mPGES-2 was expressed at similar levels in the frontal cortex (Mohri et al., 2007; Akitake et al., 2013), the MFG, parietal cortex and hippocampus (Akitake et al., 2013; Chaudhry et al., 2010) between patients with AD and control. Using immunofluorescent staining, constitutive cytoplasmic expression of mPGES-2 was detected in neurons, activated microglia, and endothelium but not in resting microglia, astrocytes, or smooth muscle cells of the MFG in control *post-mortem* human brains (Chaudhry et al., 2010). A stronger PGES-2 staining was observed in pyramidal neurons of patients with AD compared to control (Akitake et al., 2013). mPGES-2 was also found in neurons and glial cells, including astrocytes, of white matter with similar expression levels between groups (Akitake et al., 2013) (Fig. 5).

#### 3.4.3 cPGES

cPGES levels were also similar between prefrontal cortex (Mohri et al., 2007; Akitake et al., 2013), parietal cortex (Akitake et al., 2013), and hippocampus (Akitake et al., 2013) of patients with AD and control when measured by immunohistochemistry or RT-qPCR. cPGES staining was found to co-localize with markers of microglia, neurons, endothelium but not astrocytes or smooth muscle cells in the MFG of control brains, however values were not reported (Chaudhry and Dore, 2009) (Fig. 5). Staining was weak in pyramidal neurons in the MFG of *post-mortem* brains of patients with AD as compared to control, however establishing whether levels diminish progressively is not possible due to the use of end-stage cases (Chaudhry and Dore, 2009). A weak and dispersed staining was also found in the hippocampus and cortex of patients with AD (Akitake et al., 2013).

### 3.5 Phospholipases

Eight articles reported values of PLA_2_ levels in *post-mortem* brains, including calcium-dependant cytosolic PLA_2_ (cPLA_2_) and secretory PLA_2_ (sPLA2-IIA), using either enzyme assays (Ross et al., 1998; Gattaz et al., 1995; Talbot et al., 2000; Kanfer et al., 1993), mRNA expression (Colangelo et al., 2002; Moses et al., 2006) or protein levels (Sanchez-Mejia et al., 2008; Stephenson et al., 1996). Lipoprotein associated PLA_2_(Lp-PLA_2_) was assessed via immunoassay however values were not reported (Doody et al., 2015) **(**Supplementary Table 7). Results of studies reporting on PLA_2_ by any type or method have been summarized in (Fig. 4)**.**

#### 3.5.1 PLA_2_

PLA_2_ enzymatic activity either calcium dependent or independent was reported in five articles. Calcium dependent cPLA_2_ activity was significantly lower in the hippocampus (Ross et al., 1998), frontal cortex (Gattaz et al., 1995; Talbot et al., 2000), occipital cortex (Ross et al., 1998), parietal cortex (Ross et al., 1998; Gattaz et al., 1995), and temporal cortex (Ross et al., 1998) of patients with AD as compared to control. Calcium independent PLA_2_ activity was measured in the same brain regions but with only significantly lower levels in the temporal and parietal cortices (Ross et al., 1998) of patients with AD as compared to control. No changes were reported in the cerebellum for either calcium dependent cPLA_2_ or independent PLA_2_ activity between patients with AD and control. Additionally, PLA_2_ protein levels by western blot were significantly higher in the hippocampus of patients with AD compared to control (Sanchez-Mejia et al., 2008).

#### 3.5.2 cPLA_2_

*cPLA*_*2*_ level was measured by qPCR in the hippocampus and was found to be significantly higher by 350% in the CA1 region of hippocampus in patients with AD as compared to control. This finding was reported by (Colangelo et al., 2002) and presented by (Lukiw et al., 2005) using the same data set, and here we report the former. Others, using an immunohistochemical approach, found cPLA_2_ levels were significantly higher by 48.5% in the occipital cortex of patients with AD but not in the cerebellum (Stephenson et al., 1996). Finally, cPLA_2_ was mainly localized in astrocytes, while neurons, other glial cells and endothelial cells were mostly negative in the cortex of patients with AD (Stephenson et al., 1996) (Fig. 5).

#### 3.5.3 sPLA_2_

One study assessed sPLA_2_ levels by both immunohistochemistry and qPCR. mRNA levels of *sPLA2-IIA* were significantly higher in the hippocampal DG, CA3 region, and inferior temporal gyri but not in the cerebellum of patients with AD as compared to control (Moses et al., 2006). Additionally, sPLA_2_-IIA protein levels were significantly higher in astrocytes within the hippocampus (DG and CA3 regions), and inferior temporal gyrus of *post-mortem* brain with AD as compared to control, while microglia did not express the enzyme (Moses et al., 2006) (Fig. 5). Interestingly, in the grey matter of the inferior temporal gyrus, more than two thirds of sPLA_2_-IIA-positive astrocytes also co-localized with amyloid β (Aβ)-containing plaques, while in the DG, most sPLA_2_-IIA-positive astrocytes were not associated with Aβ-containing plaques (Moses et al., 2006).

#### 3.5.4 Lp-PLA_2_

Only one study reported on Lp-PLA_2_ and found no detectable levels of the enzyme in the *post-mortem* brains with AD, but its levels were higher in the blood of patients with AD as compared to the control (Doody et al., 2015).

## 4 Discussion

The analysis of the included studies revealed notable heterogeneity in the reported results of both lipid mediators and enzymes, reflecting both the complex nature of lipid mediator dysregulation in AD, and the differences in methodology between papers.

### 4.1 Changes to specific lipid mediators in AD brains

ARA-derived pro-inflammatory lipid mediators including 5-, 11-, 12- and 15-HETE oxylipins were significantly upregulated in the frontal (Furman et al., 2018; Praticò et al., 2004) and temporal (Praticò et al., 2004) cortices with no differences reported in the cerebellum (Praticò et al., 2004) or hippocampus (Wang et al., 2015). Pro-inflammatory PGD_2_ was similarly upregulated in the entorhinal corticex (Zhu et al., 2016), while PGE_2_, PGD_2_ and PGF_2α_ were significantly downregulated in the frontal cortex (Wong et al., 1992; Ebright et al., 2022) of patients with AD compared to control. In contrast, ARA-derived pro-resolving LXA4 was significantly downregulated in the hippocampus of *post-mortem* brains from patients with AD compared to control (Sun et al., 2015). Therefore, despite heterogeneity in the results, an overall elevation in HETE products, reduction in LXA4 and change in prostaglandin levels suggests that brains from patients with AD exhibited a dysregulation of ARA-derived lipid mediator pathways and a pro-inflammatory state as compared to control. Five studies reported on levels of DHA- and EPA-derived SPM. No significant differences were reported among the SPM derived from EPA, while DHA-derived RvD5, NPD1/PD1 and Maresin 1 were significantly decreased in *post-mortem* AD brains, indicating a downregulation of SPM involved in the resolution of inflammation (Lukiw et al., 2005; Zhu et al., 2016; Ebright et al., 2022). Due to limitations in study designs it is not possible to make conclusions regarding whether these changes are a cause and/or effect of AD. However, preclinical studies established through neurotoxin exposure (lipopolysaccharide, Aβ42 infusion models) and genetic manipulation (transgenic mouse models) have suggested that administration of SPM could provide neuroprotective effects against AD pathology as discussed in a recent review (Ponce et al., 2022).

In addition to enzymatic reactions, ARA and DHA are also susceptible to non-enzymatic autoxidation in the brain. DHA-derived F_4_-IsoPs were reported in 2 of 16 studies, with only one study reporting a significant elevation in the temporal and occipital cortices of patients with AD as compared to control (Nourooz-Zadeh et al., 1999; Reich et al., 2001). ARA-derived F_2_-IsoPs were reported in 11 out of 16 studies with the majority reporting significantly higher levels in several isoprostane species in the frontal (Praticò et al., 1998; Yao et al., 2003; Praticò et al., 2004), temporal (Praticò et al., 1998; Yao et al., 2003; Praticò et al., 2004) and entorhinal cortices (Forman et al., 2007), in addition to the hippocampus (Casadesus et al., 2007) with no changes in the cerebellum (Praticò et al., 1998, 2004; Reich et al., 2001; Yao et al., 2003; Bhatia et al., 2013) of patients with AD as compared to control. DHA- and ARA-derived isoprostanes are proposed biomarkers of oxidative damage and neurological diseases (Wang et al., 2015), therefore alongside significant upregulation of HETE oxylipins, these results suggest a pro-inflammatory state of brains from patients with AD.

Overall, the frontal, temporal, entorhinal cortices, and the hippocampus were the most studied brain regions representing the majority of the significant findings. This aligns with the current knowledge of AD pathology which affects the hippocampus and entorhinal cortex first before affecting the cerebral cortex responsible for language and behaviour (DeTure and Dickson, 2019). Problematically, only two of the total 16 studies, reported on lipid mediators derived from all three PUFAs: ARA, EPA, and DHA (Zhu et al., 2016; Ebright et al., 2022). The other studies reported on lipid mediators derived from one or two PUFAs. Therefore, comparison of the various lipid mediators reported between studies is quite challenging and provides a more limited representation of the brain lipid chemistry.

### 4.2 Changes to enzyme levels in AD brains more heterogenous

While 16 studies evaluated lipid mediator expression *post-mortem*, we found 38 studies reporting on the enzymes involved in the production of these mediators. Lipid mediator quantification requires more advanced LC/MS/MS methodologies due to being susceptible to oxidation and found at low concentrations in brain tissues (pg-ng) (Chiang and Serhan, 2020). In contrast, enzymes are more stable both at the mRNA and protein level, and detectable with more conventional and accessible methodologies. This could potentially explain the unbalance seen in the literature. Both 15-LOX and 5-LOX are the most abundant LOX isoforms in the human brain (Kumar et al., 2020a). Their role in AD progression is currently not fully understood as they can exert both a neurotoxic role, contributing to brain oxidative stress (Praticò et al., 2004) and neuroinflammation (Kumar et al., 2020b), and a neuroprotective role through the synthesis of pro resolving lipid mediators (Wang et al., 2015; Sun et al., 2015; Singh and Rao, 2019). Additionally, 5-LOX catalyzes the conversion of ARA to pro-inflammatory leukotrienes, which are involved in chronic inflammatory states, and the biosynthesis of anti-inflammatory lipid mediators including SPM (Gilbert et al., 2021). This uncertainty regarding the role of LOX in brain physiology makes interpretation of changes to brain levels difficult. 5-LOX was significantly upregulated in the entorhinal cortex and hippocampus of patients with AD compared to control (Firuzi et al., 2008). Pre-clinical models have shown that 5-LOX over-expression in transgenic AD mice leads to tau hyperphosphorylation, while 5-LOX inhibition reduces tau phosphorylation pointing to its contribution to the pathology (Chu and Praticò, 2011; Chu et al., 2013). Of the three studies reporting on 15-LOX levels, two reported significantly higher levels and one significantly lower levels between patients with AD and control, however the conclusions of these studies were vastly different. Pratico et al. reported an increase in the 15-LOX metabolic pathway in cortex of patients with AD as compared with control brains, and a direct correlation with an oxidative imbalance in the CNS, concluding a pro-inflammatory state (Praticò et al., 2004). Lukiw *et al*. reported a significant reduction in 15-LOX levels in the hippocampus along with NPD1, an SPM involved in the resolution of inflammation (Lukiw et al., 2005). Therefore, the researchers concluded an impairment in the resolution response in patients with AD as compared to control (Lukiw et al., 2005). Finally, Wang *et al*. reported a significantly lower levels of the anti-inflammatory LXA4 in the hippocampus of patients with AD as compared to control, with significantly higher 15-LOX expression (Wang et al., 2015). Although 15-LOX is required for the synthesis of LXA4, researchers concluded its involvement in the pro-inflammatory response may explain its higher expression among patients with AD (Wang et al., 2015). The apparent heterogeneity in the literature clearly demonstrates the limitations of looking at enzymatic expression levels alone. This suggests the need for further evaluation of the brain inflammatory state in addition to measuring enzyme levels to better understand the contribution of lipid mediators. Further, inhibition of LOX may not lead to therapeutic effects and further research is required to better understand the role of LOX in AD progression.

COX-1 and COX-2 enzymes are important for inflammatory lipid mediator biosynthesis, including prostaglandins. COX-1 is a constitutively expressed enzyme, whereas COX-2 is an inducible enzyme activated during a pro-inflammatory response (Lukiw and Bazan, 1997). Induction of COX-2 could have a central role early in the AD pathology. Indeed, inhibition of COX-2 by use of non-steroidal anti-inflammatory drugs (NSAIDs) is a proposed treatment for AD, however results are inconclusive (Cisbani and Rivest, 2021). Although NSAIDs have been shown to reduce AD pathogenesis in animal models for AD, clinical trials including RCT's assessing NSAIDs in AD show null effect (Cisbani and Rivest, 2021; Jaturapatporn et al., 2012; McGeer and McGeer, 2007). In contrast to the lipid mediators, although some heterogeneity existed, overall, there was significantly higher COX-2 protein expression in multiple brain regions of AD brains compared to control. A similar trend was seen amongst the COX-1 enzymes, however fewer studies reported significant changes. COX-2 is involved in both the pro-inflammatory and pro-resolution response (Serhan et al., 2008), therefore interpretation of elevated activity of this enzyme is difficult and cannot be used alone to determine the stage of inflammatory response. Heterogeneity amongst the results may be due to the different methodologies used (qPCR, western blot, and immunohistochemistry) (Kitamura et al., 1999; Yermakova et al., 1999) however no clear patterns were identified between the methodologies.

Three types of prostaglandin synthase isoforms have been characterized: mPGES-1 that is preferentially functionally coupled to COX-2; mPGES-2, which is constitutively expressed, and functionally coupled to COX-1 and COX-2; and cPGES, a ubiquitously and constitutively expressed cytoplasmic enzyme and functionally coupled to COX-1 (Ricciotti and Fitzgerald, 2011). Interestingly, mPGES-1 staining varied in intensity in sporadic AD cases, while familial AD cases appeared to have a more consistent level of intensity, without presenting any distinctive pattern of expression (Chaudhry et al., 2008). mPGES-1 co-localization with microglia could imply a potential role of the enzyme in Aβ phagocytosis (Chaudhry et al., 2008). mPGES-1 is preferentially coupled with COX-2 to increase the production of PGE2 (Hara et al., 2010; Murakami et al., 2000). Along with higher levels of mPGES-1 others have also reported an elevation in COX-2 protein levels in *post-mortem* brains of patients with AD (Pasinetti and Aisen, 1998; Kitamura et al., 1999). While differences were detected only for mPGES-1, no differences in mPGES-2 levels were found between patients with AD and control. Although protein levels by western blot were similar between groups, mPGES-2 staining was stronger in pyramidal neurons of *post-mortem* AD brains compared to control brains, suggesting a potential upregulation of mPGES-2 in end-stage AD. However, western blot may not be sensitive enough to detect slight changes of expression in brain sub-regions (Chaudhry et al., 2010) whereas proteomics would provide more sensitivity to assess regional differences.

PLA_2_ is a key enzyme in the metabolism of membrane phospholipids and there is a growing body of evidence pointing towards an altered phospholipid metabolism with an increased degradation of phospholipids in patients with AD (Nitsch et al., 1992; McClure et al., 1994; Kosicek and Hecimovic, 2013). PLA_2_ is also responsible for the de-esterification of PUFA from the brain phospholipids, producing free PUFA more readily available for the synthesis of bioactive lipid mediators (Klievik et al., 2023). Of the included studies, PLA_2_ activity was significantly lower in multiple cortical regions and the hippocampus of *post-mortem* brains of patients with AD compared to control (Ross et al., 1998; Gattaz et al., 1995; Talbot et al., 2000). As there is evidence of altered phospholipid metabolism in *post-mortem* AD brain (Nitsch et al., 1992; McClure et al., 1994; Kosicek and Hecimovic, 2013), this could suggest a reduced breakdown of membrane phospholipids by PLA_2_ in patients with AD. This reduction was associated with neuropathology severity, as it correlated with earlier disease onset, earlier age at death, and neurofibrillary tangles (Gattaz et al., 1995). Additionally, this reduction could be associated with the dysregulation of downstream lipid mediators, however future studies measuring PLA_2_ and lipid mediator levels in the same brain sample are required to further investigate this relationship in AD.

### 4.3 Cellular localization of enzymes involved in lipid mediator synthesis

Finally, gaining insight into the cellular localization of enzymes responsible for the synthesis of lipid mediators strengthens the evidence supporting the involvement of specific cell types in the pathogenesis of 10.13039/100020014AD. COX-2 was predominantly reported to be expressed in neurons while COX-1 was in microglia (Fig. 5). PGEs were reported in neurons, microglia and astrocytes, 15-LOX in microglia and astrocytes, and PLA_2_ in astrocytes only (Fig. 5). Phenotypic changes in microglia and astrocytes are believed to be significant contributors to the pathogenesis of neurodegenerative disorders including AD (Hopperton et al., 2018; Bazinet and Layé, 2014; Patani et al., 2023b). Consequently, alterations in enzyme levels within these specific cell types could potentially lead to the reported phenotypic changes and may offer novel avenues for therapeutic intervention.

### 4.4 Possible explanations for heterogeneity among the literature

**Fig. 6:**
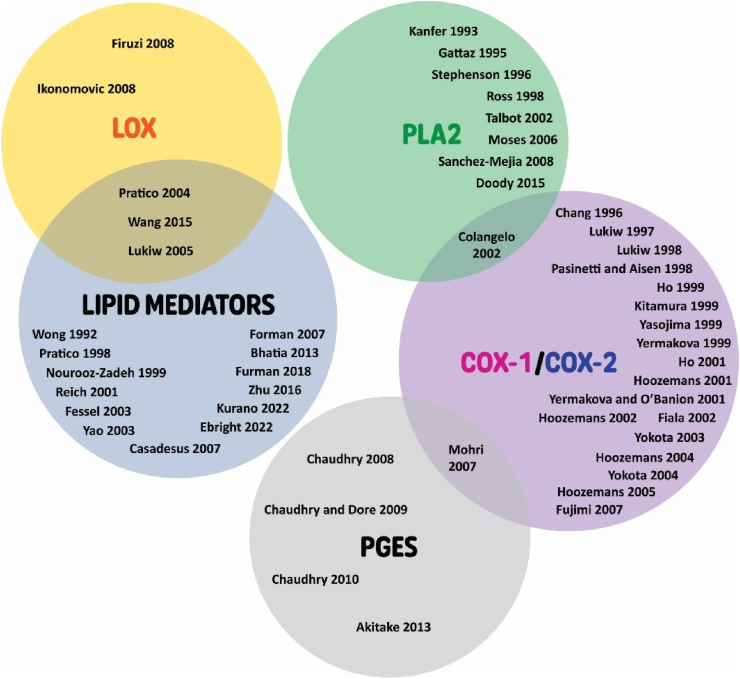
Summary results of articles reporting on levels of lipid mediators and enzymes involved in lipid mediator synthesis. Articles within regions of overlapping circles indicates reporting of multiple enzymes and lipid mediators. COX, cyclooxygenase; LOX, lipoxygenase; PGES, prostaglandin E synthase; PLA_2_, phospholipase A_2_.

Some of the heterogeneity identified between studies may be attributed to the use of NSAIDs prior to sample collection. NSAIDs are non-specific COX inhibitors and therefore would likely affect the expression levels of COX itself and its metabolites. Surprisingly, only one article reported on the use of anti-inflammatory drugs *pre-mortem* and should therefore be a factor to take into consideration for future studies (Wong et al., 1992). Additionally, differences in *post-mortem* delay may contribute to the heterogeneity of the results. Ischemia *post-mortem* causes rapid increases in brain oxylipins (within minutes of ischemia) (Trépanier et al., 2017), consequently contributing to the reported oxylipin changes which may not accurately reflect the chemical state prior to death. *Post-mortem* delay ranged from approximately 2-40 h among the studies included in this review. Interestingly one study assessed whether *post-mortem* delay could impact prostanoid production (Wong et al., 1992). The authors observed that *post-mortem* delay decreased prostanoid levels by 60% in rat brains. They did not observe any correlation between the *post-mortem* delay and prostanoid levels in the human brains, likely due to the lack of variation in *post-mortem* delay among human cases (Wong et al., 1992). Therefore, further research is warranted to better understand the effect of *post-mortem* delay on lipid mediator levels in humans. Differences in disease stage in AD pathologies and control may have further contributed to the heterogeneity (e.g. Braak stages). Disease stage is also important as the notion of “class switching” between pro-inflammatory and anti-inflammatory lipid mediators throughout disease progression may vastly change the levels and expression of the enzymes and lipid mediators, further leading to variability among the studies. The presence of concomitant pathologies with AD could also explain the discrepancy between studies as vascular or other proteinopathies (TDP-43, Lewy bodies, etc.) may affect lipid mediators and enzymes differently. Apolipoprotein ε4 allele (*APOE*4) is also a known risk factor for AD and may contribute to the heterogeneity observed (Serrano-Pozo et al., 2021). Ebright *et al.* was the only study included in this review that stratified AD patients by *APOE* genotype (Ebright et al., 2022). Although they did not show any significant differences in lipid mediator levels among the *APOE*4 compared to *APOE*3 carriers, future studies should take this into consideration (Ebright et al., 2022). Further, few studies reported on both enzymes and lipid mediator levels, which would provide a more complete understanding of the changes to the brain metabolome and potentially lead to more consistent results (Fig. 6).

Finally, different methodologies were used to measure lipid mediator and enzyme levels (GC/MS, LC/MS, immunohistochemistry, enzyme assay, mRNA expression) which further adds to the heterogeneity of the results, however no patterns comparing methods used were identified.

### 4.5 Limitations

While great effort was made to include all possible studies measuring lipid mediators and enzymes in *post-mortem* human samples from patients with AD and control, the number of papers included in our first screening may have increased the chance of misidentifying articles of interest. None of the articles included in this systematic review reported sex differences for enzymes or lipid mediator levels, despite the known sex differences that exist among AD pathology (Guo et al., 2022; Aggarwal and Mielke, 2023). Additionally, relatively few articles were found reporting on each lipid mediator and enzyme from every brain region, therefore potentially biasing the overall conclusions drawn.

## 5 Conclusion

Overall, significant upregulation of pro-inflammatory eicosanoids and oxidative products (isoprostanes), and downregulation of pro-resolution docosanoids are consistent with the previous literature suggesting a pro-inflammatory state in brains from patients with AD. Moreover, enzymes involved in the synthesis of pro-inflammatory lipid mediators were similarly upregulated, while PLA_2_ an enzyme responsible for phospholipid metabolism was downregulated in brains from patients with AD further suggesting a pro-inflammatory state and dysregulation of lipid mediator synthesis. These significant findings were primarily reported in the brain regions that are most affected by AD. Expectedly the cerebellum, which does not present significant neuropathological manifestation in AD, had no differences in the levels of the enzymes or lipid mediators measured. However, the contribution of each specific lipid mediator species and enzymes remains unclear due to the heterogeneity of the findings and the small number of studies reporting on their levels in *post-mortem* human brains. Consequently, additional studies investigating a more comprehensive panel of lipid mediators from all PUFA precursors and enzymes, while controlling for factors such as *post-mortem* delay, disease heterogeneity, anti-inflammatory drug use, disease stage and sex are required. Moreover, whether alterations in lipid mediator and enzyme levels involved in inflammation are a cause or consequence of AD remains an essential area worth further investigation. Characterization of the brain inflammatory state in conjunction with lipid mediator identification could help to provide a better understanding of the relationship between the lipid mediator profile, inflammation, and AD prognosis.

## CRediT authorship contribution statement

**Aidan D. Tyrrell:** Formal analysis, Investigation, Validation, Visualization, Writing – original draft, Writing – review & editing. **Giulia Cisbani:** Formal analysis, Investigation, Methodology, Visualization, Writing – original draft, Writing – review & editing. **Mackenzie E. Smith:** Investigation, Writing – review & editing. **Chuck T. Chen:** Visualization, Writing – review & editing. **Yue-Tong Chen:** Writing – review & editing. **Raphael Chouinard-Watkins:** Investigation, Writing – review & editing. **Kathryn E. Hopperton:** Investigation, Writing – review & editing. **Ameer Y. Taha:** Writing – review & editing. **Richard P. Bazinet:** Conceptualization, Funding acquisition, Project administration, Resources, Supervision, Writing – review & editing.

## Declaration of competing interest

The authors declare the following financial interests/personal relationships which may be considered as potential competing interests: Richard Bazinet reports financial support was provided by 10.13039/501100000024Canadian Institutes of Health Research. 10.13039/100001818RPB is supported by grant funding through the Canadian Institutes of Health Research and the 10.13039/501100000038Natural Sciences and Engineering Research Council of Canada and holds a Canada Research Chair in Brain Lipid Metabolism. RPB has received industrial grants, including those matched by the Canadian government, and/or travel support related to work on brain fatty acid uptake from Arctic Nutrition, Bunge Ltd., Capsoil Technologies, DSM, Fonterra, Mead Johnson, Natures Crops International, and Nestec Inc. Moreover, RPB is on the executive committee of the International Society for the Study of Fatty Acids and Lipids and held a meeting on behalf of fatty acids and cell signaling, both of which rely on corporate sponsorship. RPB has given expert testimony in relation to supplements and the brain. There was no role of funders in the conceptualization, design, data collection, analysis, decision to publish or preparation of the manuscript. If there are other authors, they declare that they have no known competing financial interests or personal relationships that could have appeared to influence the work reported in this paper.

## Data Availability

Data will be made available on request.
