# Supplementary material for: Lipid mediators in post-mortem brain samples from patients with Alzheimer's disease: A systematic review
Source: Brain Behav Immun Health. 2024 Dec 23;43:100938. doi: 10.1016/j.bbih.2024.100938 (PMC11782888; doi:10.1016/j.bbih.2024.100938)
Supplement: Multimedia component 3 [file mmc3.docx]

| **Section and topic** | **Item No** | **Checklist item** |
| --- | --- | --- |
| **ADMINISTRATIVE INFORMATION** | | |
| Title: |  |  |
|  | 1a | Lipid mediators in Alzheimer’s disease post-mortem brains: a systematic review protocol |
| Registration | 2 | This protocol is registered with the Open Science Framework |
| Authors: |  |  |
| Contact | 3a | Contact:  Dr. Giulia Cisbani (University of Toronto, Department of Nutritional Sciences, Toronto, Ontario; [giulia.cisbani@utoronto.ca](mailto:giulia.cisbani@utoronto.ca))  Aidan D. Tyrrell (University of Toronto, Department of Nutritional Sciences, Toronto, Ontario; aidan.tyrrell@mail.utoronto.ca)  Dr. Raphael Chouinard-Watkins (University of Toronto, Department of Nutritional Sciences, Toronto, Ontario; raphael.chouinard.watkins@utoronto.ca)  Dr. Kathryn Hopperton (University of Toronto, Department of Nutritional Sciences, Toronto, Ontario; kathryn.hopperton@mail.utoronto.ca)  Mackenzie E Smith (University of Toronto, Department of Nutritional Sciences, Toronto, Ontario; kenzie.smith@mail.utoronto.ca)  Dr. Chuck Chen (University of Toronto, Department of Nutritional Sciences, Toronto, Ontario; [tzuhuan.chen@utoronto.ca](mailto:tzuhuan.chen@utoronto.ca))  Yue-Tong Chen (University of Toronto, Department of Nutritional Sciences, Toronto, Ontario; stevenk.chen@mail.utoronto.ca)  Dr. Ameer Taha (UC Davis, Food Science and Technology, Davis, California, USA; ameer.taha@gmail.com)  Dr. Richard Bazinet (University of Toronto, Department of Nutritional Sciences, Toronto, Ontario; richard.bazinet@utoronto.ca)  Physical mailing address of corresponding author: Department of Nutritional Sciences, University of Toronto, 1 Kings College Circle, Toronto, Ontario, M5S 1A8 |
| Contributions | 3b | Mr. Aidan Tyrrell reviewed the extracted data, interpreted results, created figures and wrote the manuscript.  Dr. Cisbani developed the protocol and search strategy, executed the search, screened articles and extracted data and synthesized results and wrote the manuscript.  Ms. Smith screened articles and reviewed the extracted data and manuscript.  Dr. Chuck Chen reviewed the manuscript and prepared the tables and figures for publication.  Mr. Yue-Tong Chen helped write the introduction of the manuscript.  Dr. Chouinard-Watkins developed the protocol and search strategy, executed the search and screened articles.  Dr. Hopperton solved conflicts raised at the first and second stages of screening.  Dr. Taha revised the data collection and provide input.  Dr. Bazinet conceptualized the review and supervised data collection. |
| Amendments | 4 | July 2021: Dr. Chuck Chen added as author.  July 2021: endocannabinoid and cannabinoid receptors, PLD, PLC, Lp-PLA2 as well as lipid mediators receptors/transported excluded from extraction.  January 2023: Mr. Aidan Tyrrell and Mr. Yue-Tong Chen added as authors. |
| Support: |  |  |
| Sources/Sponsor | 5a/b | Sources/Sponsor: Natural Sciences and Engineering Research Council of Canada [482597], Canadian Institutes of Health Research. |
| Role of sponsor or funder | 5c | None |
| **INTRODUCTION** |  |  |
| **Rationale** | 6 | Lipid mediators or oxylipins are signalling molecules implicated in physiological responses, such as inflammation. These molecules are derived from polyunsaturated fatty acids, such as arachidonic acid (AA), eicosapentaenoic acid (EPA) and docosahexaenoic acid (DHA). A multitude of oxylipins are produced through different enzymatic pathways and can elicit their effects through distinct receptors. According to their origin and substrate, oxylipins are playing different roles during inflammatory response, either proinflammatory or anti-inflammatory. While neuroinflammation is likely important during the development of Alzheimer’s disease, it is still not clear which lipid mediator is playing a key role in the pathology. |
| Objectives | 7 | This review aims to bring together all the research on measures of lipid mediators in post-mortem Alzheimer’s disease brains to identify their role in this pathology.  **Review question** (Population Intervention Comparison Outcome -PICO)  **Population:** Neuropathologically confirmed Alzheimer’s Disease patients, examined post-mortem  **Intervention:** N/A  **Comparison:** neuropathologically normal controls,  **Outcome**: levels of lipid mediators and enzymes that regulate their levels |
| **METHODS** |  |  |
| Eligibility criteria | 8 | **Inclusion criteria:**  i) Type of study included: Papers published in peer-reviewed journals;  ii) Types of participants: Studies that examine the brains of human patients with Alzheimer’s Disease post-mortem (after death) will be included. Alzheimer’s disease must have been confirmed by neuropathological examination at autopsy. Controls should be neuropathologically normal;  iii) Types of outcome measures: Measurement of the levels of lipid mediators and the enzyme involved in their production in the brain at death. These include lipid mediators from the arachidonic acid, linoleic acid and DHA pathways and the enzymes responsible for their synthesis. Measures of lipid mediators and enzymes that regulate their levels detected by any technique will be included, provided the assessment occurred post-mortem.  Sex, post-mortem interval and ethnic background will be taken also in to account as pre-clinical studies showed different DHA levels between male and females under controlled dietary intake. (McNamara, 2009).  **Exclusion criteria:**  Conference proceedings and abstracts will be excluded as they do not provide sufficient information to evaluate the study. Reviews, case studies, animal studies, studies not taking place in neuropathologically confirmed Alzheimer’s Disease, papers measuring lipid mediators and enzymes outside of the brain, PET studies on living subjects and studies not using neuropathologically normal patients as a control will be excluded. |
| Information sources | 9 | Ovid will be used to query the Medline, Embase and PsychInfo databases using exp (MeSH, EMTREE) terms and title, abstract and keywords search terms specifically designed for each database. Full search terms for each database and results found per database are listed in the **Appendix 1, 2 and 3.**  Reference searches: Bibliographies of papers deemed eligible for this review will be hand searched to identify any additional eligible references, which will then be screened for title, abstract or full text as appropriate.  Initial Search and Screening:  Date of coverage: January 2020-January 2021-02-0  Extraction:  Planned date of coverage: February-April 2021  Updated Search:  Planned date of coverage: April-December 2021  Electronic Databases: Medline, Embase and PsychInfo  Grey Literature Search:  Planned Date of Coverage: May-December 2021  Updated Search:  Planned date of coverage December 2021-May 2023  Updated Search:  Planned date of coverage May 2023-April 2024 |
| Search strategy | 10 | **See Appendix 1, 2 and 3** |
| Study records: |  |  |
| Data management | 11a | Managing records (i.e. screening) will be performed in Covidence. Deduplication will be performed using Bramer method (Bramer et al., 2016) as well as Covidence |
| Selection process | 11b | Articles will then be screened using Covidence for title and abstract, and then full text following eligibility criteria. Two independent reviewers will screen the articles and they will be blind to each other’s decisions. A third reviewer will independently assess any conflict raised during the screening phases. |
| Data collection process | 11c | Data will be extracted by two independent reviewers in tables and then summarized in the result section of the review. Conflict resolution was resolved by consensus. (See **Appendix 4**). |
| Data items | 12 | The variables that we will be looking into are the levels of lipid mediators and the levels of enzymes that regulate their levels. Additionally, we will report the number of samples and the average age of the subjects included, the brain region analysed and the post-mortem delay as well as any other information that could be relevant for the review. |
| Outcomes and prioritization | 13 | Levels of lipid mediators and enzymes will be reported as percentage of difference according to the formula  (V_1_ – V_2_) / ((V_1_ + V_2_)/2) x 100  Where V_1_ is value of the AD group  And V_2_ is value of the control group |
| Risk of bias in individual studies | 14 | Only articles meeting the inclusion and exclusion criteria will be deemed eligible during the screening phases (title and abstract screening and full text screening). Two independent reviewers will screen the articles and they will be blind to each other’s decisions. A third reviewer will independently assess any conflict raised during the screening phases. Additionally, a checklist to assess factors that can bias the results of the studies including blinding, use of appropriate samples (collection, handling), use of appropriate analytical methodology, identification of cases and controls was used.  . |
| Data synthesis | 15a | The data will unlikely be homogeneous enough to evaluate similar outcomes to perform a meta-analyses. The literature will be summarized to present systematic overview of the current results and methodology used to assess the levels of lipid mediators (i.e. LC/MS and ELISA) in the brain of AD patients and their controls.  The results will be provided in tables while the main conclusion will be provided in the discussion section. |

**References**

Bramer, W.M., Giustini, D., de Jonge, G.B., Holland, L., and Bekhuis, T. (2016). De-duplication of database search results for systematic reviews in EndNote. J Med Libr Assoc *104*, 240-243.

McNamara, R.K. (2009). Evaluation of docosahexaenoic acid deficiency as a preventable risk factor for recurrent affective disorders: current status, future directions, and dietary recommendations. Prostaglandins Leukot Essent Fatty Acids *81*, 223-231.

**APPENDIX 1 – SEARCH WITH MEDLINE APPENDIX**

1. exp brain/ or exp Central nervous system/ or exp Somatosensory Cortex/ or exp Cerebellar Cortex/ or exp Motor Cortex/ or exp Prefrontal Cortex/ or exp Visual Cortex/ or exp Olfactory Cortex/ or exp Cerebral Cortex/ or exp Sensorimotor Cortex/ or exp Entorhinal Cortex/ or exp cerebrum/ or exp basal ganglia/ or exp cerebral cortex/ or exp olfactory cortex/

2. (brain*).ab,ti,kf

3. (cereb*).ab,ti,kf

4. central nervous system.ab,ti,kf

5. CNS.ab,ti,kf

6. ((cerebr* ADJ3 tissue*) or (brain ADJ3 tissue*) or (cerebral ADJ3 sample*) or (brain ADJ3 sample*)).ab,ti,kf

7. exp central nervous system/ or exp brain/ or exp blood-brain barrier/ or exp brain stem/ or exp mesencephalon/ or exp cerebral peduncle/ or exp cerebral crus/ or exp substantia nigra/ or exp pars compacta/ or exp pars reticulata/ or exp tegmentum mesencephali/ or exp cerebral aqueduct/ or exp midbrain reticular formation/ or exp pedunculopontine tegmental nucleus/ or exp oculomotor nuclear complex/ or exp edinger-westphal nucleus/ or exp periaqueductal gray/ or exp raphe nuclei/ or exp dorsal raphe nucleus/ or exp interpeduncular nucleus/ or exp midbrain raphe nuclei/ or exp red nucleus/ or exp ventral tegmental area/ or exp locus coeruleus/ or exp tectum mesencephali/ or exp inferior colliculi/ or exp posterior cerebellar commissure/ or exp pretectal region/ or exp subcommissural organ/ or exp superior colliculi/ or exp reticular formation/ or exp respiratory center/ or exp rhombencephalon/ or exp metencephalon/ or exp cerebellum/ or exp cerebellar cortex/ or exp cerebellar vermis/ or exp purkinje cells/ or exp cerebellar nuclei/ or exp cerebellopontine angle/ or exp pons/ or exp barrington's nucleus/ or exp cochlear nucleus/ or exp kolliker-fuse nucleus/ or exp middle cerebellar peduncle/ or exp pontine tegmentum/ or exp abducens nucleus/ or exp facial nucleus/ or exp parabrachial nucleus/ or exp nucleus raphe magnus/ or exp superior olivary complex/ or exp trapezoid body/ or exp trigeminal motor nucleus/ or exp trigeminal nucleus, spinal/ or exp vestibular nuclei/ or exp vestibular nucleus, lateral/ or exp myelencephalon/ or exp medulla oblongata/ or exp area postrema/ or exp olivary nucleus/ or exp nucleus raphe obscurus/ or exp nucleus raphe pallidus/ or exp solitary nucleus/ or exp trigeminal caudal nucleus/ or exp tectospinal fibers/ or exp trigeminal nuclei/ or exp cerebral ventricles/ or exp choroid plexus/ or exp ependyma/ or exp fourth ventricle/ or exp lateral ventricles/ or exp septum pellucidum/ or exp third ventricle/ or exp prosencephalon/ or exp diencephalon/ or exp epithalamus/ or exp habenula/ or exp pineal gland/ or exp hypothalamus/ or exp hypothalamic area, lateral/ or exp hypothalamus, anterior/ or exp anterior hypothalamic nucleus/ or exp organum vasculosum/ or exp paraventricular hypothalamic nucleus/ or exp preoptic area/ or exp suprachiasmatic nucleus/ or exp suprachiasmatic nucleus neurons/ or exp supraoptic nucleus/ or exp hypothalamus, middle/ or exp "arcuate nucleus of hypothalamus"/ or exp dorsomedial hypothalamic nucleus/ or exp hypothalamo-hypophyseal system/ or exp median eminence/ or exp pituitary gland/ or exp pituitary gland, anterior/ or exp corticotrophs/ or exp gonadotrophs/ or exp lactotrophs/ or exp somatotrophs/ or exp thyrotrophs/ or exp pituitary gland, intermediate/ or exp melanotrophs/ or exp pituitary gland, posterior/ or exp tuber cinereum/ or exp ventromedial hypothalamic nucleus/ or exp hypothalamus, posterior/ or exp mammillary bodies/ or exp optic chiasm/ or exp optic tract/ or exp subthalamus/ or exp entopeduncular nucleus/ or exp subthalamic nucleus/ or exp zona incerta/ or exp thalamus/ or exp thalamic nuclei/ or exp anterior thalamic nuclei/ or exp geniculate bodies/ or exp intralaminar thalamic nuclei/ or exp lateral thalamic nuclei/ or exp pulvinar/ or exp mediodorsal thalamic nucleus/ or exp midline thalamic nuclei/ or exp posterior thalamic nuclei/ or exp ventral thalamic nuclei/ or exp telencephalon/ or exp cerebrum/ or exp "diagonal band of broca"/ or exp external capsule/ or exp internal capsule/ or exp olfactory bulb/ or exp "septum of brain"/ or exp septal nuclei/ or exp telencephalic commissures/ or exp anterior cerebellar commissure/ or exp corpus callosum/ or exp meninges/ or exp ganglia/ or exp ganglia, autonomic/ or exp ganglia, parasympathetic/ or exp ganglia, sympathetic/ or exp stellate ganglion/ or exp superior cervical ganglion/ or exp ganglia, sensory/ or exp ganglia, spinal/ or exp geniculate ganglion/ or exp nodose ganglion/ or exp spiral ganglion/ or exp trigeminal ganglion/

8. (Brain* or hippocamp* or encephalon or Blood Brain Barrier or hemato-encephalic barriers or barriers brain-blood or hemato encephalic barrier or barriers hemato-encephalic or barrier hemato-encephalic or hemato-encephalic barrier or truncus cerebrus or truncus cerebri or cerebri truncus or brainstems or cerebrus truncus or Mesencephalon or mesencephalon or mesencephalons or midbrains or midbrain or Cerebral Peduncle or Cerebral Crus or Substantia Nigra or nigras substantia or nigra substantia or substantia nigras or Pars Compacta or Pars Reticulata or Tegmentum Mesencephali or midbrain trigeminal nucleus or nucleus peripeduncular or annulari nucleus or nervi trochlearis nucleus or midbrain tegmentum or mesencephalus tegmentum or tegmental nucleus ventral or mesencephalic tegmentums or midbrain tegmentums or trigeminal nucleus mesencephalic or tegmentums midbrain or trochlearis nucleus nervi or nucleus annularis or trigeminal nucleus midbrain or nucleus annular or mesencephali tegmentum or darkshevichs nucleus or tegmentums mesencephalic or ventral tegmental nucleus or mesencephalic trigeminal nucleus or nervi trochleari nucleus or nucleus darkshevich's or darkschewitsch nucleus or tegmentum of midbrain or nucleus annulari or cajal interstitial nucleus or mesencephalic tegmentum or nuclei accessory oculomotor or trochlear nucleus or annularis nucleus or nucleus mesencephalic trigeminal or nucleus of darkschewitschor peripeduncular nucleus or oculomotor nuclei accessory or tegmentum midbrain or tegmentum mesencephali or nucleus nervi trochlearis or darkshevich nucleus or nucleus tractus mesencephalici nervi trigemini or interstitial nucleus of cajal or Cerebral Aqueduct or ducts mesencephalic or mesencephalic ducts or aqueduct mesencephalic or sylvian aqueducts or duct mesencephalic or sylvius aqueduct or cerebrus aqueductus or aqueductus cerebrus or cerebral aqueduct or aqueduct sylvian or aqueduct of sylvius or mesencephalic duct or cerebral aqueducts or aqueducts sylvian or aqueduct cerebral or sylvian aqueduct or aqueductus cerebri or aqueducts mesencephalic or cerebri aqueductus or mesencephalic aqueduct or Midbrain Reticular Formation or Pedunculopontine Tegmental Nucleus or nucleus tegmentalis pedunculopontinus or nucleus pedunculopontine tegmental or tegmental nucleus pedunculopontine or pedunculopontine tegmental nucleus or Oculomotor Nuclear Complex or Edinger-Westphal Nucleus or Periaqueductal Grey or greys central periaqueductal or griseum centrales or central grey substance of midbrain or periaqueductal greys central or grey matter periaqueductal or grey central periaqueductal or substantia grisea centralis or periaqueductal grey matter or central periaqueductal grey or grisea centralis substantia or periaqueductal grey or centrale mesencephali griseumor centrale mesencephalus griseum or centrale griseum or grey matters periaqueductalor centrales griseum or periaqueductal grey central or substantia grisea centralis mesencephali or mesencephalus griseum central or midbrain central grey or central grey mesencephalic or central periaqueductal greys or central grey midbrain or griseum centrale mesencephali or Raphe Nuclei or nucleus incertus or nucleus superior central ornuclei raphe ornucleus interfascicular or superior central nucleus or raphe nuclei or interfascicular nucleus or raphe nucleus or incertus nucleus or central nucleus superior or rostral linear nucleus of the raphe or caudal linear nucleus of the raphe or rostral linear nucleus of raphe or nucleus rapheor Dorsal Raphe Nucleus or Interpeduncular Nucleus or Midbrain Raphe Nuclei or Red Nucleus or nucleus ruber or red nucleus or nucleus red or Ventral Tegmental Area or tegmentalis ventralis area or tegmentalis ventrali area or area tegmentalis ventralis or ventral tegmental area of tsai or ventral tegmental area or tegmental area ventral or area tegmentalis ventrali).ti,ab,kf

9. (Locus Coeruleus or coeruleus complex locus or complices locus coeruleus or locus caeruleus or complex locus ceruleus or complices locus ceruleus or coeruleus complices locus or ceruleus complex locus or locus ceruleus complex or complex locus coeruleus or locus ceruleus complices or locus ceruleus or nucleus pigmentosus pontis or locus coeruleus complices or pontis nucleus pigmentosus or ceruleus complices locus or locus coeruleus or locus coeruleus complex or Tectum Mesencephali or corpora quadrigemina or inferior colliculus commissures or colliculus commissures superior or colliculus commissures inferior or quadrigeminal plates or superior colliculus commissure or plate quadrigeminal or commissure of superior colliculus or quadrigemina corpora or commissure of inferior colliculus or lamina quadrigemina or inferior colliculus commissure or colliculus commissure inferior or quadrigeminal plate or tectum mesencephalus or mesencephalus tectum or plates quadrigeminal or quadrigemina lamina or colliculus commissure superior or Inferior Colliculi or colliculi inferior or inferior colliculi or inferiors colliculus or posterior colliculus or brachial nucleus of the inferior colliculus or caudal colliculus or colliculus inferiors or colliculus caudal or inferior colliculus or colliculus posterior or colliculus inferior or Subcommissural Organ or subcommissural organs or subcommissural organ or organs subcommissural or organ subcommissural or Superior Colliculi or mammalian optic lobesor optic lobe mammalian or optic tectums or superior colliculi or optic tectum or anterior colliculus or colliculus superior or human optic lobes or superior colliculus or optic lobes human or optic lobes mammalian or optic lobe human or colliculi superior or tectum optic or tectums optic or mammalian optic lobe or human optic lobe or colliculus anterior or Reticular Formation or formations reticular or reticular formation or reticular formations or formation reticular or edunculopontine Tegmental Nucleus or nucleus tegmentalis pedunculopontinus or nucleus pedunculopontine tegmental or tegmental nucleus pedunculopontine or pedunculopontine tegmental nucleus or Respiratory Center or centers respiratory or respiratory centers or center respiratory or respiratory center or hombencephalon or hind brains or brains hind or rhombencephalons or hindbrain or hindbrains or brain hind or rhombencephalon or hind brain or Medulla Oblongata or medulla oblongata or nucleus ambiguous or arcuate nucleus-1 or accessory cuneate nucleus or nucleus external cuneate or cuneate nucleus accessory or nucleus ambiguous or medulla oblongatas or arcuate nucleus of the medulla or cuneate nucleus lateralor nucleus lateral cuneate or ambiguous nucleus or cuneate nucleus external or arcuate nucleus 1 or external cuneate nucleus or ambiguus nucleus or arcuate nucleus-1s or lateral cuneate nucleus or Area Postrema or area postremas or trigger zone chemoreceptor or chemoreceptor trigger zone or chemoreceptor trigger zones or trigger zones chemoreceptor or zone chemoreceptor trigger or postrema area or zones chemoreceptor trigger or area postrema or Olivary Nucleus ornucleus basalis olivary or nucleus olivary or basalis olivary nucleus or nucleus olivary basal or olivary basal nucleus or basal nucleus olivary or olivary nucleus or Raphe nuclei or nucleus incertus or nucleus superior central or nuclei raphe or nucleus interfascicular or superior central nucleus or raphe nuclei or interfascicular nucleus or raphe nucleus or incertus nucleus or central nucleus superior or rostral linear nucleus of the raphe or caudal linear nucleus of the raphe or rostral linear nucleus of raphe or nucleus raphe or Nucleus Raphe Obscurus or Nucleus Raphe Pallidus or Solitary Nucleusor solitary nuclear complices or nucleus of tractus solitaries or complex solitary nuclear or tractus solitarii nuclei or nucleus solitaries or solitarius nucleus tractus or tractus solitarius nucleus or solitarius nuclei tractus or solitary tract nucleus or nucleus solitary tract or solitary nuclear complex or tractus solitarius nuclei or nuclear complices solitary or nuclei tractus solitarii or solitary nucleus ornucleus solitaryor nucleus of the solitary tract or nuclear complex solitary or complices solitary nuclear or nucleus of solitary tract or nucleus tractus solitaries or Trigeminal Nucleus, Spinal or trigeminal nucleus spinal or nucleus spinal trigeminal or spinal trigeminal nucleus or Trigeminal Caudal Nucleus or caudal nucleus trigeminal or nucleus trigeminal caudal or trigeminal caudal nucleus or Metencephalon or Cerebellumor corpus cerebellus or parencephalons or cerebellus corpus or cerebellum or cerebellums or corpus cerebelli or parencephalon or cerebelli corpus or Cerebellar Cortex or cerebelli cortex or cortex cerebellus or cerebellar cortex or cortex cerebelli orcerebellus cortex or cortex cerebellar or Cerebellar Vermis or Purkinje Cells or purkinje cells or cells purkinje or Cerebellar Nuclei or nucleus dentatus or Cerebellopontine Angle or central nucleus or central nucleus or interposed nucleus anterior or nucleus globosus or medial cerebellar nucleus or emboliformis nucleus or nuclei cerebellar or intracerebellar nuclei or nucleus fastigii or nucleus fastigial or fastigii nucleus or central nuclei or nuclei central or deep cerebellar nucleus or intracerebellar nucleus or nucleus fastigial cerebellar or nucleus anterior interposed or nucleus intracerebellar oranterior interposed nucleus or nucleus anterior interpositus or nucleus medial cerebellar or nuclei intracerebellar or nucleus dentate or dentate nucleus or interpositus nucleus anterior or globosus nucleus orcerebellar nucleus deep or nucleus central or nucleus cerebellar or cerebellar nuclei deep or nucleus dentate cerebellar or anterior interpositus nucleus or cerebellar nucleus medial or cerebellar nuclei or fastigial cerebellar nucleus or Pons or pons or varolii ponsor pontes or pons varolius or varolius pons or pons varolii or ponte or Barrington's Nucleusor Cochlear Nucleus or cochlear nucleus or nuclei cochlear or cochlear nuclei or nucleus cochlear or Kolliker-Fuse Nucleus or Middle Cerebellar Peduncle or Pontine Tegmentum or Abducens Nucleus or Facial Nucleus or Parabrachial Nucleus or Nucleus Raphe Magnus or Superior Olivary Complex or Trapezoid Body or Trigeminal Motor Nucleus or Vestibular Nuclei or schwalbes nucleus or nucleus schwalbe or vestibular nuclei or vestibular nucleus medial or nuclei vestibular or schwalbe's nucleus or nucleus schwalbe's or medial vestibular nucleus or schwalbe nucleus or nucleus medial vestibular or Vestibular Nucleus, Lateral or deiters nucleus or deiter's nucleus or nucleus of deiters or lateral vestibular nucleus or nucleus lateral vestibular or vestibularis laterali nucleus or nucleus vestibularis laterali or vestibular nucleus lateral or vestibularis magnocellulari nucleus or vestibularis magnocellularis nucleus or deiter nucleus or nucleus vestibularis magnocellularis or nucleus vestibularis magnocellulari or nucleus deiter or nucleus vestibularis lateralis or vestibularis lateralis nucleus or nucleus deiter's or Tectospinal Fibers or Trigeminal Nuclei or trigeminal nucleus or trigeminal nuclear complices or trigeminal nuclear complex or nuclei trigeminal or trigeminal nuclei or nucleus trigeminal or nuclear complices trigeminal or nuclear complex trigeminal or Grey Matter or grey matter or grey matters cerebellar or grey matter cerebellar or matters grey or matter cerebellar grey or grey matter cerebellar or cerebellar grey matters or grey matters or matter cerebellar grey or cerebellar grey matter or matters grey or cerebellar grey matters or matters cerebellar grey or grey matters cerebellar or matters cerebellar grey or grey matter or cerebellar grey matter or matter grey or matter grey or White Matter or white matter cerebellar or matter cerebellar white or matter white or matters cerebellar white or white matters cerebellar or cerebellar white matters or cerebellar white matter or matters white or white matter or white matters or Cerebral Ventricles or cerebral ventricle or cerebral ventriclesor monro foramen or ventricles cerebral or foramen of monro or cerebral ventricular system or ventricle cerebral or Choroid Plexus or choroideus plexus or plexus choroideusor choroid plexus or chorioid plexus or plexus chorioid or plexus choroid or Ependyma or ependymal or ependymas or Fourth Ventricle or ventricolo quarto or ventricles fourth or ventricle fourth or 4th ventricle or quarto ventricolos or ventricle 4th or ventricles 4th or fourth ventricle or ventricolos quarto or fourth ventricles or 4th ventricles or quarto ventricolo or Lateral Ventricles or lateral ventricle orsubventricular zones or lateral ventricles or ventricle lateral or zone subventricular or ventricles lateral or subventricular zone or zones subventricular or Septum Pellucidum or septum supracommissural or pelusidum septum or septum pellucidum or lucidums septum or supracommissural septum or pellucidum septum or septum pelusidums or septum pelusidum or pelusidums septum or septum lucidums or supracommissural septums or septums supracommissural or lucidum septum or septum lucidum or Third Ventricle or 3rd ventricle or ventricles third or ventricles 3rd or third ventricle or ventricle 3rd or 3rd ventricles or third ventricles or ventricle third or Limbic System or limbic system or system limbic or systems limbic or limbic systems or Amygdala or amygdaloid bodies or corpus amygdaloideums or nucleus intercalated amygdaloid or corpus amygdaloideum or amygdaloid body or complex amygdaloid nuclear or amygdaloid nuclear complices or amygdaloid nucleusor intercalata massa or amygdaloideums corpus or intercalatas massa or amygdaloid nucleus intercalated or nuclear complices amygdaloid or archistriatums or amygdala or massa intercalates or nucleus amygdaloid or amygdaloideum corpus or amygdalae nucleus or nuclear complex amygdaloid or archistriatum or nucleus amygdalae or amygdaloid nuclear complex or Basolateral Nuclear Complex or Central Amygdaloid Nucleus or Corticomedial Nuclear Complex or Periamygdaloid Cortex or epithalamus or Habenula or commissure habenular or habenula complex or habenulas or complices habenula or nucleus habenularis or habenular commissures or complex habenula or habenula complices or nucleus habenular or nucleus habenulari or commissures habenular or habenula or habenularums commissura or commissura habenularum or habenularis nucleus or habenular nuclei or commissura habenularums or nuclei habenular or habenulari nucleus or habenular nucleus or Pineal Gland or pineales corpus or body pineal or glands pineal or pineal glands or pineal body or cerebri epiphysis or corpus pineales or gland pineal or pineale corpus or bodies pineal or corpus pineale or pineal gland or pineal bodies or epiphysis cerebri).ti,ab,kf

10. (Hippocampus or hippocampal formation or propers hippocampus or hippocampus propers or formations hippocampal or horn ammon's or schaffer collateral or ammon horn or hippocampus or horn ammon or cornu ammonis or hippocampus proper or proper hippocampus or collaterals schaffer or formation hippocampal or hippocampal formations or subiculum or subiculums or ammon's horn or CA1 Region, Hippocampal or regio superior of hippocampus or field hippocampus ca1 or ca1 stratum radiatum or stratum radiatum ca1 or hippocampal sector ca1 or hippocampus ca1 field or hippocampus regio superior or ca1 stratum radiatums or sector ca1 hippocampal or ca1 field hippocampus or radiatums ca1 stratum or stratum radiatums ca1 or ca1 hippocampal sector or ca1 pyramidal cell area or ca1 region hippocampal or ca1 pyramidal cell layer or ca1 stratum pyramidale or stratum pyramidale ca1 or cornu ammonis 1 area or radiatum ca1 stratum or CA2 Region, Hippocampal or ca2 stratum pyramidale or radiatums ca2 stratum or cornu ammonis 2 area or ca2 field hippocampus or stratum pyramidale ca2 or stratum radiatum ca2 or ca2 stratum radiatums or radiatum ca2 stratum or sector ca2 hippocampal or region hippocampal ca2 or ca2 field of hippocampus or stratum radiatums ca2 or ca2 region hippocampal or hippocampal sector ca2 or hippocampal ca2 region or hippocampus ca2 field or ca2 pyramidal cell layer or field hippocampus ca2 or ca2 pyramidal cell area or CA3 Region, Hippocampal or stratum lucidum ca3 or ca3 stratum lucidum or stratum lucidums ca3 or lucidum ca3 stratum or ca3 region hippocampal or ca3 pyramidal cell area or hippocampus ca3 field or ca3 hippocampal sector or sector ca3 hippocampal or ca3 stratum radiatum or ca3 stratum lucidums or hippocampal ca3 regions or cornu ammonis 3 area or ca3 field of hippocampus or radiatum ca3 stratum or field hippocampus ca3 or stratum radiatums ca3 or ca3 pyramidal cell layer or lucidums ca3 stratum or region hippocampal ca3 or radiatums ca3 stratum or ca3 stratum pyramidale or ca3 field hippocampus or Dentate Gyrus or ca4 region hippocampal or dentate fascia or cornu ammonis 4 area or hilus gyri dentate or ca4 field of hippocampal formation or ca4 hippocampal sector or gyrus dentate or sector ca4 hippocampal or hippocampal ca4 region or area dentata or region hippocampal ca4 or dentata area or field hippocampal ca4 or gyrus dentatus or hilus of the fascia dentata or hilus of dentate gyrus or dentate gyrus or area dentatas or dentata fascia or hippocampal sector ca4 or hippocampal ca4 field or ca4 of lorente de no or Mossy Fibers, Hippocampal or hippocampal mossy fiber or mossy fibers hippocampal or hippocampal mossy fibers or mossy fiber hippocampal or Fornix, Brain or hippocampal commissure or hippocampal commissures or commissures dorsal hippocampal or fornix commissures or fornices or brain fimbrias or fornical commissures or fornical commissure or fornix or hippocampal commissures dorsal or commissures hippocampal or fornix-fimbria or hippocampal commissure dorsal or fimbria or fornix fimbria or fimbria of hippocampus or brain fornices or dorsal hippocampal commissure or commissure fornical or commissure dorsal hippocampal or commissure of fornix or commissures fornical or commissure hippocampal or fornix commissure or fimbria-fornix or fimbria fornix or fimbria brain or hippocampus fimbrias or hippocampus fimbria or brain fimbria or Hypothalamus or preoptico-hypothalamic areas or preoptico hypothalamic area or lamina terminalis or hypothalamus or areas preoptico-hypothalamic or area preoptico-hypothalamic or preoptico-hypothalamic area or Hypothalamic Area, Lateral or area hypothalamica laterali or hypothalamica laterali area or hypothalami area lateralis or lateralis area hypothalamica or hypothalamus area lateralis or laterali area hypothalamica or areas lateral hypothalamic or lateralis hypothalami area or lateral hypothalamic areas or accessory nucleus of the ventral horn or lateral tuberal nuclei or tuberal nucleus lateral or lateral hypothalamus or area hypothalamica lateralis or hypothalamus lateral or tuberomammillary nucleus or hypothalamic area lateral or nucleus tuberomammillary or nuclei lateral tuberal or nucleus lateral hypothalamic or lateralis hypothalamus area or area lateral hypothalamic or hypothalamic nucleus lateral or area lateralis hypothalamus or nucleus lateral tuberal or Hypothalamus, Anterior or commissures anterior hypothalamic or anterior hypothalamic decussation of ganser or hypothalamic commissures anterior or anterior hypothalamic commissures or commissure anterior hypothalamic or periventricular nucleus anteroventral or nucleus anteroventral periventricular or anterior hypothalamic commissure or hypothalamic commissure anterior or hypothalamus anterior or hypothalamus supraoptic or anteroventral periventricular nucleus or anterior hypothalamus or supraoptic hypothalamus or Anterior Hypothalamic Nucleus or areas anterior hypothalamic or hypothalamic area anterior or nucleus anterior hypothalamic or anterior hypothalamic nucleus or hypothalami nucleus anterior or hypothalamic areas anterior or anterior hypothalami nucleus or anterior hypothalamic area or area anterior hypothalamic or nucleus anterior hypothalamus or hypothalamus nucleus anterior or anterior hypothalamic areas or anterior hypothalamus nucleus or nucleus anterior hypothalami or hypothalamic nucleus anterior).ab,ti,kf

11. (Organum Vasculosum or Paraventricular Hypothalamic Nucleus or hypothalamic paraventricular nucleus or paraventricular hypothalamic nucleus or nucleus paraventricular hypothalamic or nucleus hypothalamic paraventricular or nucleus paraventricular or paraventricular nucleus or hypothalamic nucleus paraventricular or paraventricular nucleus hypothalamic or Preoptic Area or area medial preoptic or preoptic area medial or preoptic nucleus or nuclei preoptic or lateral preoptic area or preoptic areas lateral or area preoptic or areas medial preoptic or area lateral preoptic or preoptic areas medial or lateral preoptic areas or preoptica area or nucleus preoptic or medial preoptic areas or areas lateral preoptic or area preoptica or areas preoptic or preoptic nuclei or medial preoptic area or preoptic area or preoptic areas or Suprachiasmatic Nucleus or nucleus suprachiasmatic or suprachiasmatic nucleus or Supraoptic Nucleus or hypothalamus supraoptic nucleus or supraoptic group accessory or accessory supraoptic groups or supraoptic nucleus of hypothalamus or supraopticus nucleus or groups accessory supraoptic or nucleus supraoptic or group accessory supraoptic or accessory supraoptic group or nucleus supraopticus or supraoptic groups accessory or supraoptic nucleus or Hypothalamus, Middle or regions intermediate hypothalamic or hypothalamic region intermediate or region intermediate hypothalamic or middle hypothalamus or hypothalamus medial or hypothalamic regions intermediate or intermediate hypothalamic regions or intermediate hypothalamic region or hypothalamus middle or medial hypothalamus or Arcuate Nucleus of Hypothalamus or nucleus arcuate or arcuate nucleus or hypothalamus arcuate nucleus or nucleus infundibular or infundibular nucleus or arcuate nucleus of hypothalamus or Dorsomedial Hypothalamic Nucleus or nucleus arcuate or arcuate nucleus or hypothalamus arcuate nucleus or nucleus infundibular or infundibular nucleus or arcuate nucleus of hypothalamus or Hypothalamo-Hypophyseal System or hypothalamic pituitary unit or hypothalamo hypophyseal system or hypothalamo-hypophyseal system or hypothalamic-pituitary unit or Median Eminence or eminentia medianas or median eminence or eminences medial or eminence medial or medial eminences or medianas eminentia or eminentia mediana or mediana eminentia or eminence median or medial eminence or Pituitary Gland or hypophyseal infundibulum or infundibular hypothalamus or pituitary glands or infundibulum or stalk infundibular or hypothalamus infundibular or infundibulums or pituitary stalks or pituitary gland or hypophysis or pituitary stalk or infundibular stem or stalks infundibular or glands pituitary or hypophysis cerebri or hypophyseal stalks or cerebri hypophysis orstalk hypophyseal or infundibular stalk or infundibular stalks or hypophysis cerebrus or hypophyseal stalk or Pituitary Gland, Anterior or lobus anteriors or anterior lobe of pituitary or anterior lobus or pituitary pars distalis or anterior pituitary glands or anteriors lobus or lobus anterior or pituitary gland anterior or adenohypophyses or pituitary glands anterior or adenohypophysis or pituitary anterior lobe or anterior pituitary gland or pars distalis of pituitary or Corticotrophs or Gonadotrophs or lh producing cells or lh-secreting cells or fsh cells or gonadotrophs or lh cell or fsh-secreting cellsor fsh secreting cells or fsh-producing cells or fsh-producing cell or fsh cell or lh-producing cells or fsh producing cells or lh secreting cells or fsh-secreting cell or gonadotroph or lh-producing cell or lh-secreting cell or lh cell or Lactotrophs or pituitary prolactin-secreting cells or lactotrophs or pituitary prolactin cell or prolactin-secreting cell pituitary or prolactin-secreting cells pituitary or lactotroph or prolactin cell pituitary or prolactin cells pituitary or pituitary prolactin cells or pituitary prolactin-secreting cell or pituitary prolactin secreting cells or Somatotrophs or gh cell pituitary or somatotrophs or gh cells pituitary or pituitary growth hormone-secreting cells or pituitary gh cell or pituitary growth hormone secreting cells or pituitary gh cells or somatotroph or Thyrotrophs or Pituitary Gland, Intermediate or Melanotrophs or Pituitary Gland, Posterior or lobes neural or posterior pituitary glands or neural lobe or pituitary pars nervosa or infundibular processes or infundibular process or process infundibular or neurohypophysis or lobe neural or gland posterior pituitary or pituitary posterior lobe or pars nervosa of pituitary or posterior lobe of pituitary or neural lobes or nervosus lobus or lobus nervosus or pituitary gland posterior or processes infundibular or Tuber Cinereum or cinereums tuber or cinereum tuber or tuber cinereum or tuber cinereums or Ventromedial Hypothalamic Nucleus or nucleus ventromedial hypothalamic or hypothalamic nucleus ventromedial or ventromedial hypothalamic nucleus or Hypothalamus, Posterior or posteriors area hypothalamica or area hypothalamica posterior or mammillary regions or region mammillary or nucleus posterior periventricular or hypothalamic regions posterior or hypothalamus posteriors or mammillary region or posterior area hypothalamica or posterior hypothalamic regions or supramammillary commissures or region posterior hypothalamic or supramammillary commissure or regions posterior hypothalamic or posterior hypothalamus or commissures supramammillary or premammillary nucleus or hypothalamic region posterior or posterior hypothalamic region or commissure supramammillary or hypothalamus posterior or hypothalamica posteriors area or periventricular nucleus posterior or nucleus premammillary or Mammillary Bodies or mammillary bodies ormammillary body or body mammillary or mamillary bodies or body mamillary or bodies mamillary or bodies mammillary or mamillary body or Limbic Lobe or Gyrus Cinguli or gyrus cingular or anterior cingulate gyrus or cingulate gyri posterior or cortex anterior cingulate or posterior cingulate gyrus or cinguli anteriors gyrus or mesial region superior or gyrus cingulate or cingulate cortex anterior or cingulate cortex or superior mesial regions or regions cingulate or cortex posterior cingulate or anterior cingulate cortices or posterior cingulates or cingulate bodies or cingulates anterior or cortices anterior cingulate or posterior cingulate cortices or mesial regions superior or posterior cingulate cortex or regions posterior cingulate or cingulate posterior or posterior cingulate region or region posterior cingulate or body cingulate or cortex cingulate or posterior cingulate regions or cingulate gyrus anterior or cingulate gyrus or cingulate gyrus posterior or cingular gyrus or bodies cingulate or cingulate area or anterior cingulates or area cingulate or cingulate regions or regions superior mesial or ingulates posterior or areas cingulate or cingulate cortices anterior or anterior gyrus cinguli or gyri posterior cingulate or gyrus anterior cingulate or gyrus cinguli anteriors or cinguli anterior gyrus or superior mesial region or anterior cingulate or gyrus cinguli anterior or cingulate anterior or region cingulate or cingulate areas).ti,ab,kf.

12. (Parahippocampal Gyrus or gyrus parahippocampal or gyri parahippocampal or parahippocampal gyri posterior or hippocampal gyrus or gyri posterior parahippocampal or posterior parahippocampal gyrus or gyrus parahippocampalis or parahippocampal gyrus uncus or presubiculums or posterior parahippocampal gyri or gyrus posterior parahippocampal or parahippocampal gyrus posterior or uncus of parahippocampal gyrus or gyri hippocampal or parahippocampal gyrus or presubiculum or gyrus hippocampi or uncus parahippocampal gyrus or gyrus uncus parahippocampal or gyrus hippocampal or parahippocampal gyri or Entorhinal Cortex or area entorhinali or areas entorhinal or entorhinalis area or entorhinal area or area entorhinal or entorhinal cortices or area entorhinalis or cortices entorhinal or entorhinali area or olfactory cortices secondary or secondary olfactory cortex or cortex secondary olfactory or cortices secondary olfactory or entorhinal cortex or olfactory cortex secondary or secondary olfactory cortices or cortex entorhinal or entorhinal areas or Olfactory Pathways or olfactory pathways or pathways olfactory or olfactory pathway or pathway olfactory or Perforant Pathway or pathway perforant or pathways perforant or perforant paths or perforant pathways or perforant pathway or fasciculus perforating or paths perforant or perforant path or perforating fasciculus or path perforant or Septum of Brain or paraterminal body or brain septums or brain septum or septum of brain or paraterminal bodies or area septal or bodies paraterminal or body paraterminal or septal area or region septal or septal region or Septal Nuclei or nucleus of the stria terminalis or septi lateralis nucleus or septal nuclear complices or nucleus of anterior commissure or terminali nucleus striae or laterali nucleus septalis or nucleus lateralis septi or nucleus lateralis septus or nuclear complices septal or septofimbrial nucleus or diagonal band nucleus or nucleus septofimbrial or nucleus septi lateralis or laterali nucleus septi or nucleus triangular septal or medial septal nucleus or nucleus of diagonal band or nucleus septalis lateralis or nucleus striae terminali or nuclear complex septal or septum nucleus lateral or lateral septal nucleus or lateralis nucleus septalis or septal nucleus lateral or septalis laterali nucleus or nuclei septal or anterior commissure nucleus or septus nucleus lateralis or septi laterali nucleus or nucleus medial septum or nucleus septalis laterali or triangularis septus nucleus or lateralis nucleus septi or complex septal nuclear or nucleus striae terminalis or nucleus of stria terminalis or septalis lateralis nucleus or dorsal septal nucleus or nucleus triangularis septus or nucleus lateral septumor nucleus triangularis septi or nucleus lateral septal or septal nucleus triangular or terminalis nucleus striae or septi nucleus lateralis or septi nucleus triangularis or triangular septal nucleus or septus nucleus triangularis or nucleus medial septal or complices septal nuclear or lateralis septus nucleus or medial septum nucleus or lateralis septi nucleus or Substantia Innominata or innominata substantia or substantia innominata or Prosencephalon or prosencephalon or forebrains or forebrain or Diencephalon or diencephalon or interbrain or interbrains or Optic Chiasm or chiasmas optic or optic chiasms or decussation optic or chiasma optic or optic chiasm or optic chiasma or optic decussation or opticums chiasma or optic decussations or chiasma opticum or decussations optic or opticum chiasma or optic chiasmas or chiasma opticums or chiasms optic or chiasm optic or Optic Tract or Subthalamus or subthalamus or fasciculus thalamic or field h nucleus or campi forelus nucleus or fasciculus lenticular or field h1 forel's or campi foreli nucleus or enticular fasciculus or forels field h2 or forel field h2 or thalamicus fasciculus or fasciculus thalamicus or thalamic fasciculus or forelus nucleus campi or nucleus of ansa lenticularis or foreli nucleus campi or nucleus campi forelus or nucleus of field h or forels field h1 or forel's field h2 or field h1 of forel or forel field h1 or Entopeduncular Nucleus or Subthalamic Nucleus or nucleus of luys or luys subthalamic nucleus or corpus luysi or luys body or subthalamic nucleus of luys or subthalamicus nucleus or luys nucleus or nucleus subthalamic or luysi corpus or body of luys or nucleus subthalamicus or subthalamic nucleus or Zona Incerta or Thalamus or thalamencephalon or thalamencephalons or thalamus or Thalamic Nuclei or nuclei thalamic or thalamic nuclei or Anterior Thalamic Nuclei or nucleus anterodorsal thalamic or anterior nuclear group or nucleus anteromedial thalamic or nucleus anteroventral thalamic or thalamus anterior nucleus or anterior thalamic nucleus or nucleus anteroventral or anteroventral nucleus or thalamic nucleus anterodorsal or nuclei anterior thalamic or thalamic nuclei anterior or anteromedial nucleus or anteromedial thalamic nucleusor thalamus anterior or nucleus anteromedial or anterodorsal nucleus or anterior thalamus or anterior thalamic nuclei or anterodorsal thalamic nucleus or nucleus anterodorsal or thalamic nucleus anteroventral or Geniculate Bodies or nucleus geniculate or medial geniculate nucleus or geniculate complex medial or geniculatum mediales corpus or bodies geniculate or nucleus lateral geniculate or mediales corpus geniculatum or geniculate bodies medial or mediale corpus geniculatum or geniculate body or geniculatum mediale corpus or geniculate nucleus lateral or geniculate bodies or geniculate bodies lateral or metathalamus or corpus geniculatum mediale or geniculate body lateral or complex medial geniculate or nucleus geniculatus lateralis pars dorsalis or geniculate body medial or geniculate complices medial or geniculate nucleus or complices medial geniculate or medial geniculate body or medial geniculate bodies or geniculate nucleus medial or Intralaminar Thalamic Nuclei or nucleus paracentrali or centrum medianum or paracentrali nucleus or centromedian thalamic nucleus or central lateral nucleus or thalamic nucleus parafascicular or central lateral thalamic nucleus or parafascicular thalamic nucleus or thalamic nucleus intralaminar or nucleus central dorsal or parafascicular nucleus of the thalamus or centromedian nucleus or intralaminar nuclei rostral or intralaminar nuclear group or thalamic nucleus centromedian or parafascicularis nucleus or nucleus central lateral or thalamic nuclei intralaminar or central dorsal thalamic nucleus or interlaminar nuclei of thalamus or rostral intralaminar nuclei or thalamus nucleus parafascicularis or centrum medianums nucleus or medianum centrum or thalamic nucleus paracentral or thalamus reticulate nucleus or nucleus paracentral or nucleus central medial or paracentral thalamic nucleus or median nucleus centre or nuclei intralaminar thalamic or nuclei rostral intralaminar or central medial nucleus or nucleus centrum medianums or nucleus centre median or medianum nucleus centrum or nucleus paracentral thalamic or nucleus centromedian thalamic or nucleus parafascicularis thalamus or nucleus intralaminar thalamic or nucleus centrum medianum or nucleus parafascicularis thalami or parafascicularis thalami nucleus or parafascicularis thalamus nucleus or reticulate nuclei of thalamus or nucleus parafasciculari or centrum medianums or centrum medianum nucleus or paracentralis nucleus or lateral nucleus central or parafascicular nucleus or central medial thalamic nucleus or nucleus centromedian or Lateral Thalamic Nuclei or medial pulvinar nucleus or Pulvinar or anterior pulvinar nucleus).ab,kf,ti.

13. (pulvinar nucleus or nucleus anterior pulvinar or lateral pulvinar nucleus or pulvinar nucleus oral or oral pulvinar nucleus or pulvinar nucleus inferior or pulvinars or pulvinari nucleus or nucleus oral pulvinar or nucleus pulvinar or pulvinar thalami or nucleus pulvinari or nucleus lateral pulvinar or thalami pulvinar or nucleus inferior pulvinar or pulvinaris nucleus or nucleus pulvinaris or pulvinar thalamus or inferior pulvinar nucleus or pulvinar nucleus lateral or Mediodorsal Thalamic Nucleus or medialis dorsali nucleus or medial dorsal thalamic nucleus or mediodorsal nucleus or dorsomedialis thalamus nucleus or nucleus dorsomedial thalamic or nuclei medial thalamic or dorsali nucleus medialis or nucleus mediodorsal or nucleus dorsomedialis thalamus or nucleus medialis dorsali or nucleus mediodorsal thalamic or thalami nucleus dorsomedialis or thalamus nucleus dorsomedialis or thalamic nucleus medial or dorsomedialis thalami nucleus or thalamic nuclei medial or nucleus medial thalamic or mediodorsal thalamic nucleus or nucleus dorsomedial or nucleus medialis dorsalis or thalamic nucleus mediodorsal or dorsal medial nucleus or medialis dorsalis nucleus or nucleus dorsomedialis thalami or medial thalamic nucleus or Midline Thalamic Nuclei or parataenial nucleus or nucleus reunien or rhomboid nucleusor nucleus subfascular or nuclear group midline or paratenial nucleus or rhomboidal nucleus or rhomboid thalamic nucleus or nucleus rhomboid thalamic or reuniens nucleus or subfascular nucleus or thalamus nucleus reuniens or thalami nucleus reuniens or nucleus paraventricular thalamic or reunien nucleus or reuniens thalami nucleusor paraventricular nucleus of thalamus or paraventricular thalamic nucleus or midline thalamic nucleus or thalamic nuclei midline or paratenial thalamic nucleus or thalamic nucleus rhomboid or periventricular nuclei of thalamus or thalamic nucleus reuniens or reuniens thalamus nucleus or thalamus midline nucleus or nucleus paratenial or thalamus paraventricular nucleus or midline thalamic nuclei or thalamic nucleus subfascular or nucleus reuniens thalamus or thalamic nucleus paratenial or nucleus reuniens or nucleus rhomboid or thalamic nucleus paraventricular or midline nuclear group or Posterior Thalamic Nuclei or supergeniculate nucleus or posterior nuclear complicesor nucleus supergeniculateor posterior thalamic nuclei or suprageniculate thalamic nucleus or submedial nucleus or limitans nucleus or thalamic nuclei posterior or nucleus limitan or thalamic nucleus suprageniculate or nucleus submedial or nuclear complices posterior or complices posterior nuclear or posterior nucleus of thalamus or nucleus limitans or nucleus suprageniculate thalamic or posterior thalamic nucleus or posterior thalamic nuclear group or posterior nuclear complex or nuclear complex posterior or thalamus posterior nucleus or Ventral Thalamic Nuclei or posterior nucleus ventral or ventrolateral thalamic nucleus or intermedius nucleus ventralis or ventral posterior nucleus or ventralis intermedius nucleus or ventrobasal complex or ventralis posteromediali nucleus or nuclear mass ventral or group ventral nuclear or ventralis posterolateralis nucleus or nucleus ventral anterior or ventral nuclear groups or laterali nucleus ventralis or nucleus ventral posterolateral or ventralis posterior nucleus or masses ventral nuclear or nucleus ventralis posterolaterali or ventral lateral nucleus or nucleus ventralis intermedius or ventral anterior thalamic nucleus or thalamic nucleus ventral or posterolaterali nucleus ventralis or ventral posteromedial thalamic nucleus or nucleus ventrolateralis thalamus or ventrobasal complices or nucleus ventralis posteromedialis or nuclei ventral thalamic or nucleus ventrolateralis thalami or mass ventral nuclearor ventrolateralis thalami nucleus or ventrolateralis thalamus nucleus or posterolateral nucleus ventral or nuclear group ventral or arcuate nucleus 3 or nucleus ventralis posteriors or ventral posterior thalamic nucleus or ventral posterior medial nucleus or ventral posteroinferior nucleus or posteroinferior nucleus ventral or posteriors nucleus ventralis or arcuate nucleus-3 or thalamus nucleus ventrolateralis or nucleus ventralis or posteromediali or complex ventrobasal or ventral lateral thalamic nucleus or ventral thalamic nuclei or ventral lateral thalamic nuclei or ventral posteromedial nucleus or posteromedialis nucleus ventralis or ventral anterior nucleus or ventral posterolateral nucleus or nuclear masses ventral or ventral posterior inferior thalamic nucleus or thalamic nucleus ventrolateral or ventral nuclear group or thalamus ventrolateral or thalami nucleus ventrolateralis or posteromediali nucleus ventralis or posterolateralis nucleus ventralis or posterior nucleus ventralis or nucleus ventral posteromedial or nucleus ventralis laterali or ventral posterolateral thalamic nucleus or nucleus ventral thalamic or ventralis lateralis nucleus or ventral nuclear mass or ventralis posteriors nucleus or ventralis laterali nucleus or nucleus ventral posterior or ventral thalamic nucleus or ventrolateral thalamus or nucleus ventralis lateralis).ab,ti,kf.

14. (Telencephalon or telencephalon or endbrain or endbrains or Cerebrum or cerebrum or cerebral hemisphere left or cerebral hemisphere right or cerebral hemispheres or right cerebral hemisphere or cerebral hemisphere or left cerebral hemisphere or Basal Ganglia or ganglia basal or nuclei basal or basal ganglia or ganglion basal or basal nuclei or claustrum or Corpus Striatum or lenticular nucleus or nucleus lentiform or lentiformis nucleus or lentiform nucleus or corpus striatum or nucleus lenticular or nucleus lentiformis or lentiform nuclei or striatum corpus or nuclei lentiform or Globus Pallidus or pallidum or paleostriatum or globus pallidus or pallidums or Neostriatum or Caudate Nucleus or nucleus caudatus or caudate nucleus or caudatus nucleusor nucleus caudate or caudatus or High Vocal Center or Putamen or putamens or nucleus putamens or putamens nucleus or putamen nucleus or nucleus putamen or putamen or Ventral Striatum or Nucleus Accumbens or nucleus accumbens or accumbens septus nucleus or accumbens septi nucleusor nucleus accumbens septi or septi nucleus accumbens or accumbens nucleus or septus nucleus accumbens or nucleus accumbens septus or Olfactory Tubercleor Islands of Calleja or Basal Nucleus of Meynert or nucleus basalis of meynert or meynert basal nucleus or nucleus basalis magnocellularis or basal nucleus of meynert or meynert nucleus basalisor Cerebral Cortex or plates cortical or insular cortex or cerebral cortices or archipalliums or paleocortex or allocortices or periallocortices or plate cortical or cerebri cortex or cortices cerebral or paleocortices or cortices insular or insular cortices or cortex insular or periallocortex or archipallium or cortical plates or cortex cerebral or cortex cerebri or reil insula or cortex cerebrus or cortical plate or Frontal Lobe or gyrus anterior centralor central gyrus anterior or lobe frontalor frontal lobeor cortex frontal or gyrus precentralis or frontal eye fieldor supplementary eye field or gyrus precentrali or frontali lobusor precentrali gyrus or frontal lobes or frontal cortex or field supplementary eye or lobes frontal or eye field supplementary or lobus frontali or supplementary eye fields or frontalis lobus or gyrus precentral or eye fields supplementary or eye fields frontal or anterior central gyrus or fields frontal eye or lobus frontalis or Motor Cortex or motor area or primary motor cortex or motor area precentral or strip motor or somatomotor areas or strips motor or motor cortices primary or premotor areas or motor area secondary or cortex precentral motor or motor area somatic or supplementary motor areas or area primary motoror area premotor or secondary motor area or motor cortices secondary or area motor or secondary motor areas or area somatomotor or motor areas or motor cortex secondary or precentral motor areas or cortices secondary motor or area supplementary motor or motor areas supplementary or area precentral motor or cortices primary motor or precentral motor cortices or areas somatic motor or area somatic motor or areas motor or motor cortex precentral or motor areas precentral or motor strips or cortex primary motor or somatomotor area or premotor area or precentral motor cortex or primary motor area or somatic motor area or motor areas somatic or areas premotor or areas somatomotor or areas precentral motor or areas supplementary motor or motor cortex primary or cortex secondary or motor primary motor cortices or motor cortex or motor cortices precentral or motor area supplementary or cortices precentral motor or somatic motor areas or cortex motor or areas secondary motor or Prefrontal Cortex or orbital gyrus).ab,kf,ti.

15. (gyrus orbital or sulcus olfactoryor convolutions superior frontal or orbitofrontal cortices lateral or gyrus frontalis superior or rectal gyrusor cortices ventromedial prefrontal or orbital cortices or cortex orbital or prefrontal cortices ventromedial or inferiors gyrus frontalis or orbital gyri or orbital area or convolution superior frontal or frontalis superiors gyrus or inferior frontal gyrus or gyri orbitofrontal or orbitofrontal regions or frontalis inferiors gyrus or frontal sulcus or prefrontal cortex ventromedial or straight gyrus or cortex lateral orbitofrontal or gyrus frontalis inferior or sulci olfactory or orbital areas or orbitofrontal gyri or area orbital or orbitofrontal region or cortices lateral orbitofrontal or lateral orbitofrontal cortex or superior frontal convolution or cortex orbitofrontal or medial frontal gyrus or gyrus orbitofrontal or gyrus straight or superior frontal gyrus or frontal gyrus medial or ventromedial prefrontal cortex or gyrus rectal or subcallosal area or olfactory sulcus or prefrontal cortex or superior frontal convolutions or sulcus frontal or olfactory sulci or region orbitofrontal or superiors gyrus frontalis or superior gyrus frontalis or gyrus superior frontal ororbitofrontal cortex or frontal gyrus inferior or gyrus frontalis inferiors or cortex ventromedial prefrontal or marginal gyrus or rectus gyrus or orbital cortex or gyrus medial frontal or orbitali gyrus or orbitofrontal gyrus or inferior gyrus frontalis or frontal gyrus superior or gyri orbital or areas orbital or cortex prefrontal or cortices orbital or gyrus rectus or frontalis superior gyrus or lateral orbitofrontal cortices or orbitofrontal cortices or gyrus frontalis superiors or orbitofrontal cortex lateral or gyrus marginal or Broca Area or Neocortex or neocortical molecular layer or neocortices cerebral or isocortex or cerebral neocortices or neopalliumsor corticalis substantiaor multiform layer neocortical or neocortical multiform layer or layer neocortical molecular or cortices neopallial or neopallial cortex or neocortical internal pyramidal layer or molecular layer neocortical or neopallial corticesor cortex neopallial or layers neocortical multiform or neocortex cerebralor molecular layers neocortical or neocortical internal granular layer or neocortical multiform layers or cerebral neocortex or neocortical external pyramidal layer or neocortical molecular layers or isocortices or external granular layer or substantia corticali or corticali substantia or layer neocortical multiformor multiform layers neocortical or Occipital Lobe or occipital cortex or cuneus or gyrus annectant or sulcus lunate or gyrus lingual or gyrus occipitalor calcarine fissures or regions occipital or occipitotemporal gyrus medial or sulcus calcarine or lunate sulcus or occipital lobe or gyrus medial occipitotemporal or cuneate lobule or region occipital or fissures calcarine or calcarinus sulcus or sulcus calcarinus or lobe occipital or lobes occipital or occipital region or gyrus lingualis or occipital gyrus or annectant gyrus or occipital regions or lobules cuneate or occipital sulcus or cortices occipital or calcarine sulcus or fissure calcarine or lingual gyrus or lobule cuneate or cortex cuneus or linguali gyrus or Visual Cortex or primary visual cortices or visual cortex primaries or primaries visual cortex or cortices extrastriate or visual cortices primary or extrastriate cortices or cortex primaries visual or cortex primary visual or striate cortex or cortices primary visual or visual cortex primary or visual cortex or cortex striate or cortex extrastriate or cortex visual or extrastriate cortex or primary visual cortex or Olfactory Cortex or Basal Forebrain or Piriform Cortex or sulcus intraparietal or regions parietal or lobes parietal or paracentral lobules posterior or gyrus supramarginal or precuneus cortices or parietal cortex or gyrus angulari or praecuneus or gyrus angularis or gyrus supramarginali or parietal regions or gyrus prelunate or lobules parietal or lobe parietal or parietal cortices posterior or gyrus supramarginalis or angulari gyrus or supramarginali gyrus or marginal sulcus or posterior parietal cortex or prelunate gyrus or posterior parietal cortices or intraparietal sulcus or angularis gyrus or region parietal or parietal lobules or precuneus or cortex parietal or gyrus angular or precuneus cortex or lobule parietal or parietal lobule or cortices precuneus or posterior paracentral lobule or lobules posterior paracentral or sulcus marginal or posterior paracentral lobules or Parietal Lobe or sulcus intraparietal or regions parietal or lobes parietal or paracentral lobules posterior or gyrus supramarginal or precuneus cortices or parietal cortex or gyrus angulari or praecuneus or gyrus angularis or gyrus supramarginali or parietal regions or gyrus prelunate or lobules parietal or lobe parietal or parietal cortices posterior or gyrus supramarginalis or angulari gyrus or supramarginali gyrus or marginal sulcus or posterior parietal cortex or prelunate gyrus or posterior parietal cortices or intraparietal sulcus or angularis gyrus or region parietal or parietal lobules or precuneus or cortex parietal or gyrus angular or precuneus cortex or lobule parietal or parietal lobule or cortices precuneus or posterior paracentral lobule or lobules posterior paracentral or sulcus marginal or posterior paracentral lobules or omatosensory Cortex or postcentral gyrus or somatosensory cortices primary or somatosensory cortex primary or cortex anterior parietal or cortices anterior parietal or cortices primary somatosensory or anterior parietal cortices or cortex secondary sensory or areas primary somatosensory or primary somatosensory cortices or cortex si or gyrus post central or secondary somatosensory areas or post central gyrus or parietal cortices anterior or gyrus postcentrali or secondary somatosensory cortex or somatosensory cortex or areas secondary somatosensory or somatosensory cortex secondary or area primary somatosensory or postcentralis gyrus or cortices secondary sensory or secondary sensory cortex or primary somatosensory cortex or somatosensory areas secondary or si cortex or primary somatosensory areas or secondary somatosensory cortices or gyrus postcentralis or area secondary somatosensory or primary somatosensory area or cortex primary somatosensory or secondary somatosensory area or secondary sensory cortices or cortices secondary somatosensory or primary somatic sensory area or postcentrali gyrus or gyrus postcentral or Wernicke Area or Sensorimotor Cortex or Auditory Cortex or gyrus transverse temporal or auditory areas temporal or temporal auditory areas or areas auditory or cortex primary auditory or transverse temporal gyrus or cortex auditory or auditory areas or transverse temporal gyri or convolutions heschl's or auditory cortex or auditory area or auditory cortex primary or auditory cortices primary or areas temporal auditory or area auditory or gyri transverse temporal or primary auditory cortices or heschl gyri or heschl convolutions or temporal gyri transverse or auditory area temporal or temporal auditory area or heschls gyri or primary auditory cortex or Temporal Lobe or temporal operculums or superior temporal gyrus or occipito-temporal gyrus lateral or gyrus fusiform or gyrus lateral occipito-temporal or gyrus temporalis superior or occipitotemporal gyrus or horns temporal or inferior horn of lateral ventricle or temporal sulcus or regions temporal or operculums temporal or cortex temporal or lobes temporal or planum polares or temporalis superior gyrus or gyrus superior temporal or horn temporal or lobe temporal or temporal horn or temporal cortices or gyrus lateral occipitotemporal or temporalis superiors gyrus or region temporal or fusiformi gyrus or temporal region or temporal horns or lateral occipito-temporal gyrus or temporal cortex or gyrus temporal or temporal operculum or temporal regions or operculum temporal or cortices temporal or temporal horn of the lateral ventricle or polare planum or fusiformis gyrus or sulcus temporal or polares planum or planum polare or temporal lobe or Diagonal Band of Broca or diagonal band of broca or broca diagonal band or External Capsule or olfactory tracts or olfactory tract lateral or main olfactory bulbs or bulbs main olfactory or bulb olfactory or glomerulus olfactory or lateral olfactory tracts or olfactory tract or bulb main olfactory or olfactory bulbs or olfactory bulb main or accessory olfactory bulb or accessory olfactory bulbs or tracts olfactory or bulbs accessory olfactory or olfactory glomerulus or bulbs olfactory or olfactory bulb accessory or tract olfactory or bulb accessory olfactory or tract lateral olfactory or olfactorius bulbus or olfactory bulb or lateral olfactory tract or Olfactory Bulb or olfactory tracts or olfactory tract lateral or main olfactory bulbs or bulbs main olfactory or bulb olfactory or glomerulus olfactory or lateral olfactory tracts or olfactory tract or bulb main olfactory or olfactory bulbs or olfactory bulb main or accessory olfactory bulb or accessory olfactory bulbs or tracts olfactory or bulbs accessory olfactory or olfactory glomerulus or bulbs olfactory or olfactory bulb accessory or tract olfactory or bulb accessory olfactory or tract lateral olfactory or olfactorius bulbus or olfactory bulb or lateral olfactory tract or Telencephalic Commissures or Anterior Cerebellar Commissure or Corpus Callosum or corpus callosums or commissures neocortical or neocortical commissures or corpus callosum or callosums corpus or interhemispheric commissure or interhemispheric commissures or neocortical commissure or callosum corpus or commissures interhemispheric or commissure interhemispheric or commissure neocortical or Internal Capsule or internal capsules or interna capsula or capsules internal or capsule internal or capsula internas or internal capsule or capsula interna or internas capsula or Myelencephalon).ab,kf,ti.

16. 1 or 2 or 3 or 4 or 5 or 6 or 7 or 8 or 9 or 10 or 11 or 12 or 13 or 14 or 15

17. exp Alzheimer disease/ or exp Plaque, Amyloid/ or exp Amyloid beta-Peptides/ or exp Amyloid/ or exp Amyloid Neuropathies/ or exp Neurofibrillary Tangles/ or exp tau proteins

18. alzheimer*.ti,ab,kf

19. ase.ti,ab,kf.

20. ((mild adj2 cognitive adj2 impairment) or MCI).ti,ab,kf.

21. (cognitive adj2 decline).ti,ab,kf.

22. AD.ti,ab,kf.

23. ((sporadic ADJ2 Alzheimer*) or (sporadic ADJ2 AD) or (early onset ADJ2 Alzheimer*) or (early onset ADJ2 AD) or familial Alzheimer* or (familial ADJ2 Alzheimer*) or (familial ADJ1 AD) or early onset AD or late onset AD or (late onset ADJ2 Alzheimer*) or (late onset ADJ2 Alzheimer disease) or (late onset ADJ2 AD) or EOAD or LOAD or autopsy confirmed Alzheimer* or (autopsy ADj2 Alzheimer*)).ti,ab,kf

24. ((dementia ADJ4 Alzheimer*) or (senile dementia ADJ4 Alzheimer*) or (dementia ADJ4 AD) or (senile dementia ADJ4 AD) or Alzheimer sclerosis or Alzheimer syndrome or (focal onset ADJ2 Alzheimer*) or (focal onset ADJ2 AD) or (presenile dementia) or (presenile dementia ADJ4 Alzheimer) or (presenile dementia ADJ4 AD) or beta amyloid plaque* or beta-amyloid plaque* or amyloid beta or beta amyloid or beta-amyloid or Abeta or amyloid deposit* or amyloid plaque* or neuritic plaque* or senile plaque* or Alzheimer* disease associated protein* or AD-AP or ADAP or AD-AP or (beta protein ADJ3 Alzheimer*) or (amyloid beta ADJ3 Alzheimer*) or (amyloid-beta ADJ3 Alzheimer*) or amyloid fibril protein* or amyloid beta peptide* or amyloid beta protein* or amyloid-beta peptide* or amyloid-beta protein* or neurofibrillary tangle*or tau protein* or taupath* or NFT or (tangle* ADJ3 Alzheimer*) or tau or neurofibrillary pathology* or Alzheimer disease-associated neurofibrillary pathology or braak staging or (braak ADJ3 Alzheimer*) or CERAD or (CERAD ADJ3 Alzheimer*)).ti,ab,kf.

25. 17 or 18 or 19 or 20 or 21 or 22 or 23 or 24

26. exp phospholipases/ or exp lysophospholipase/ or phospholipases a/ or exp type c phospholipases/ or exp phospholipase d/ or exp phospholipases a1/ or exp phospholipases a2/ or exp 1-alkyl-2-acetylglycerophosphocholine esterase/ or exp phospholipases a2, calcium-independent/ or exp group vi phospholipases a2/ or exp peroxiredoxin vi/ or exp phospholipases a2, cytosolic/ or exp group iv phospholipases a2/ or exp phospholipases a2, secretory/ or exp group i phospholipases a2/ or exp group ia phospholipases a2/ or exp group ib phospholipases a2/ or exp group ii phospholipases a2/ or exp group iii phospholipases a2/ or exp group v phospholipases a2/ or exp group x phospholipases a2/

27. exp Arachidonate 15-Lipoxygenase/ or exp Arachidonate 12-Lipoxygenase/ or exp Lipoxygenase/ or exp Arachidonate 5-Lipoxygenase/ or exp lipoxygenases/ or exp arachidonate lipoxygenases/ or exp endocannabinoids/

28. exp prostaglandin-endoperoxide synthases/ or exp cyclooxygenase 1/ or exp cyclooxygenase 2/ or exp prostaglandins/ or exp prostaglandin endoperoxides/ or exp prostaglandins a/ or exp prostaglandins b/ or exp prostaglandins d/ or exp prostaglandins e/ or exp prostaglandins f/ or exp prostaglandins i/ or exp prostaglandins, synthetic/

29. exp Oxylipins/ or exp lipid metabolism/ or exp fatty acids/ or exp decanoic acids/ or exp eicosanoic acids/ or exp eicosanoids/ or exp arachidonic acids/ or exp arachidonic acid/ or exp hydroxyeicosatetraenoic acids/ or exp isoprostanes/ or exp leukotrienes/ or exp thromboxanes/ or exp eicosapentaenoic acid/ or exp 5,8,11,14-eicosatetraynoic acid/ or exp 8,11,14-eicosatrienoic acid/ or exp lipoxins/ or exp prostaglandins/

30. ((Arachidonic Acid ADJ 2 metabolite*) or ARA metabolite* or AA metabolite* or Arachidonic Acid or ARA or AA or eicosaHydroxyeicosatetraenoic acid* or eicosa-Hydroxyeicosatetraenoic acid* or Hydroxyeicosatetraenoic acid* or HETE or 5-HETE or 5HETE or 5 HETE or 12-HETE or 12HETE or 12 HETE or 8-HETE or 8HETE or 8 HETE or 9-HETE or 9HETE or 9 HETE or 15-HETE or 15HETE or 15 HETE or 20-HETE or 20HETE or 20 HETE or 19-HETE or 19HETE or 19 HETE or 5-Hydroxyeicosatetraenoic acid* or 15-Hydroxyeicosatetraenoic acid* or 12-Hydroxyeicosatetraenoic acid* or 9-Hydroxyeicosatetraenoic acid* or 8-Hydroxyeicosatetraenoic acid*  20-Hydroxyeicosatetraenoic acid or docosanoid* or Eicosanoid* or 19-Hydroxyeicosatetraenoic acid*).ab,ti,kf.

31. (Leukotriene* or LTA4 or LTB4 or LTC4 or LTD4 or LTE4 or LTX4 or LTA5 or LTB5 or LTC5 or LTD5 or LTE5).ab,ti,kf.

32. (Eoxin* or EXA4 or EXC4 or EXD4 or EXE4 or Prostanoid* or Prostaglandin* or PG* or PGG2 or PGH2 or PGE2 or PGD2 or PGF2* or PGA* or PGA1 or PGA2 or PGB2 or Prostacyclin* or Dihomo-2-series prostanoid* or prostaglandin D1 or prostaglandin D2 or prostaglandin D3 or PGD1 or PGD3 or PGD* or dihomo-prostaglandin* or dihomo-PGD2 or dihomo-PGE2 or dihomo-PGI2 or PGI2 or PGI3 or PGH2 or PGE1 or PGE3 or PGE* or Cyclopentenone prostaglandin* or delta12-PGJ* or 15-deoxy-delta 12,14-PGJ2 or dihomo-gamma-linolenic acid* or PGF3* or 15-keto-PGE* or 6-keto PGF1* or PGI3 or Thromboxane* or TXA2 or TXB2 or TXA1 or TXA3 or txb3).ab,ti,kf.

33. ((EPA ADJ2 Oxylipin*) or (eicosapentaenoic acid ADJ2 oxylipin*) or Resolvin* or resolvin D1 or resolvin D2 or D series resolvin* or RVD* or RvE* or 18S-RvE1 or hydroxyeicosatrienoic acid* or 5-hydroxyeicosatrienoic acid* or 12-hydroxyeicosatrienoic acid* or 15-hydroxyeicosatrienoic acid* or 5-HETrE or 15-HETrE or 12-HETrE or 5-oxo-ETrE or 15-deoxy- PGJ2 or 15-deoxy-Prostaglandin*).ab,ti,kf.

34. (Oxoeicosanoid* or Hydroperoxyeicosatetraenoic acid* or Hydroperoxy-eicosatetraenoic acid* or eicosatetraenoic acid* or hPETE* or 5-Hydroperoxyeicosatetraenoic acid* or 5-Hydroperoxy-eicosatetraenoic acid* or 5-HpETE or 5HPETE or 5 HPETE or 5-oxo-ETE or 8-hpete or 12-hpete or 15-hpete or 8hpete or 12hpete or 15hpete or 8 hpete or 12 hpete or 15 hpete or 12-oxo-ETE or 15-oxo-ETE or 5-hydroxy-eicosatetraenoic acid* or 12-hydroxy-eicosatetraenoic acid* or 8-hydroxy-eicosatetraenoic acid* or 15-hydroxy-eicosatetraenoic acid* or oxo-eicosatetraenoic acid* or oxo-ETE* or 5-oxo-eicosatetraenoic acid* or 15-oxo-eicosatetraenoic acid* or 12-oxo-eicosatetraenoic acid* or dihydroxy derivatives or 5,15-dihydroxy-eicosatetraenoic acid or 5,15-DiHETE or 8,15-dihydroxy-eicosatetraenoic acid or 8,15-DiHETE or 12,20-dihydroxy-eicosatetraenoic acid or 12,20-DiHETE or 5-LOX, 5-lypoxygenase activating protein or FLAP or Hepoxilin* or HxA3 or HxB3 or 12-HpETE or 8,9-dihydroxy-eicosatetraenoic acid or 8,9-DiHETE or 14,15-dihydroxy-eicosatetraenoic acid or 14,15-DiHETE or 17,18-dihydroxy-eicosatetraenoic acid or 7,18-DiHETE).ab,ti,kf.

35. (Lipoxin* or LXA* or LXB* or (hydroperoxy ADJ1 acid*) or Epi-lipoxin* or 15-Epi-lipoxin* or Epilipoxin* or 15- Epilipoxin* or 15-epi-LxA4 or AT-LxA4 or 15-epi-LxB4 or AT-LxB4 or Epoxyeicosatrienoic acid* or Epoxy-eicosatrienoic acid* or epoxyeicosatrienoate or epoxy-eicosatrienoate or EET* or 5,6-EET or 8,9-EET or 11,12-EET or 14,15-EET or 5,6-EEQ or 8,9-EEQ or 11,12-EEQ or 14,15-EEQ or 15,16-EEQ or Isoprostane* or isoPs or PG-like ADJ1 compound* or PG-like ADJ1 product* F_2_-isoprostane* or F_2_-IsoPs or F_4_-neuroprotane* or F_4_-NPs D2-isoPs or E2-isoPs or A2-isoPs or J2-isoPs or 5,6-epoxyisoprostane E2 or 5,6-epoxyisoprostane A2 or Isofuran* or dihydroxyeicosatrienoic or DHET* or soluble hydroxylase or 5-oxo-eicosatetraenoic acid or 5-oxo-ete).ab,ti,kf.

36. ((Linoleic acid ADJ2 Oxylipin*) or oxylipin* or OXLAM* or oxidized Linoleic Acid metabolites or  oxidized LA metabolites or ARA oxylipin* or (Arachadinoic Acid ADJ2 Oxylipin*) or AA oxylipin* or DHA oxylipin or (Docosahexaenoic acid ADJ2 oxylipin*) or COX oxylipin* or LOX oxylipin* or CYP oxylipin* or protectin* or NPD1 or neuroprotectin* or 17-epi-PD1 or maresin* or mar1 or mar2 or 7-epi Maresin* or 7-epi-mar1 or mar-l1 or mar-l2 or marl1 or marl2 or Oxylipin* or DHA oxylipin* or eicosapentaenoic acid- derived RvE1 or EPA-derived RvE1 or hydroxydocosahexaenoic acid* or HDoHE or protectin D1 or PD1 or linoleic acid-derived 9-hydroxyoctadecadienoic acid* or LNA-derived 9-hydroxyoctadecadienoic acid* or linoleic acid-derived 13-hydroxyoctadecadienoic acid* or LNA-derived 13-hydroxyoctadecadienoic acid* or hydroxyoctadecadienoic acid* or HODE or 9-HODE or 13-HODE or 9,10-dihydroxy-octadecenoic or 13-oxo-octadecadienoic acid* or 9-oxo-octadecadienoic acid* or 13-oxo-ODE or 9-oxo-ODE or 9-HpODE or 9-hydroperoxy-10E,12Z-octadecadienoic acid or 13-HpODE or 13-hydroperoxy-10E,12Z-octadecadienoic acid).ab,ti,kf

37. (Hydroxy Fatty acid*or hydroxyl FA or 11-HETE or 11HETE or 11 HETE or 11-oxo-ETE or 13-HDoHE or 13HDoHE or 11-hydroxy-eicosatetraenoic acid* or 13-hydroxy-docosahexaenoic acid* or 9-hydroxy-octadecadienoic acid* or 17-hydroxy Docosahexaenoic Acid* or 17-HDoHE or 17-HDHA or hydroperoxyl-DHA or hydorperoxyl- docosahexaenoic acid or hp-DHA or hpDHA or 17-hydroperoxy-docosahexaenoic acid or 17-hydroperoxydocosahexaenoic acid or hydroxydha or 14-hpdha or 17s-hpdha or 14s-hpdha or hydroperoxy-docosahexaenoic acid or hydroxydocosahexaenoic acid or HDHA or 17-HDHA or 13-oxo-dha or 17-oxo-dha).ab,ti,kf.

38. (epoxy-eicosatrienoic acid* or EpETrE or epoxyeicosatetraenoic acid* or 5,6-epoxy-eicosatrienoic acid* or 5,6-EpETrE or 5,6-epoxyeicosatetraenoic acid* or 8,9- epoxy-eicosatrienoic acid* or 8,9-EpETrE or 8,9-epoxyeicosatetraenoic acid* or 11,12-epoxy-eicosatrienoic acid* or 11,12-EpETrE or 11,12-epoxyeicosatetraenoic acid* or 14,15-epoxy-eicosatrienoic acid* or 14,15-EpETrE or 14,15-epoxyeicosatetraenoic acid* or epoxy-eicosatetraenoic acid* or EpETE or EpDPE or EDP or HETE or hydroxyeicosapentaenoic acid* or hydroxy-eicosapentaenoic acid* or hydroxy eicosapentaenoic acid* or HEPE* or 5-hepe or 12-hepe or 15-hepe or 20-hepe or 5hepe or 12hepe or 15hepe or 20hepe or 8-HEPE or 8HEPE or 8 HEPE or 9-HEPE or 9HEPE or 9 HEPE or 18-HEPE or 18HEPE or 18 HEPE or HDoHE or dihydroxy-eicosatrienoic acid* or dihydroxyeicosatrienoic acid* or DiHETrE or 5,6-dihydroxy-eicosatrienoic acid* or 5,6-dihydroxyeicosatrienoic acid* or 8,9-dihydroxy-eicosatrienoic acid* or 8,9-dihydroxyeicosatrienoic acid* or 11,12-dihydroxy-eicosatrienoic acid* or 11,12-dihydroxyeicosatrienoic acid* or 14,15-dihydroxy-eicosatrienoic acid* or 14,15-dihydroxyeicosatrienoic acid* or 8,9-DiHETrE or 5,6-DiHETrE or 11,12-DiHETrE or 15,16-DiHETrE or DiHETE or dihydroxy-docosapentaenoic acid* or dihydroxydocosapentaenoic acid* or 5-oxo- eicosapentaenoic acid* or 5-oxo-EPE or oxo-eicosapentaenoic acid* or oxo-EPE or hydroperoxy-eicosapentaenoic acid* or HpEPE* or 12-hydroperoxy-eicosapentaenoic acid* or 15-hydroperoxy-eicosapentaenoic acid* or 12-HpEPE or 15-HpEPE).ab,ti,kf.

39. (dihydroxy-octadecatrienoic acid* or dihydroxyoctadecatrienoic acid* or dihydroxy octadecatrienoic acid* or diHOTrE or hydroxy-octadecatrienoic acid* or hydroxyoctadecatrienoic acid* or hydroxy octadecatrienoic acid* or HOTrE or Hydroperoxy-octadecatrienoic acid* or Hydroperoxyoctadecatrienoic acid* or hydroperoxy octadecatrienoic acid* or HpOTrE or oxo-octadecatrienoic acid* or oxooctadecatrienoic acid* or oxo octadecatrienoic acid* or oxo-OTrE or dihydroxy-octadecadienoic acid* or dihydroxyoctadecadienoic acid* or dihydroxy octadecadienoic acid*).ab,ti,kf

40. (specialized pro-resolving mediator* or specialized proresolving mediator* or lipid mediator* or specialised pro-resolving mediator* or specialised proresolving mediator*).ab,ti,kf

41. (Endocannabinoid* or ethanolamide* or n-acylethanolamide* or n acylethanolamide* or fatty acid ethanolamide* or cannabinoid* or endocannabinoid-like or Endocannabinoid system* or cannabinoid receptor* or CB1 or CB2 or Arachidonoylethanolamine* or 2-Arachidonoylglycerol or 2-Arachidonyl glyceryl ether* or O-arachidonoyl-ethanolamine* or N-Arachidonoyl dopamine* or ethanolamine* or aminoethanol* or n-acylethanolamine* or n acylethanolamine* or fatty acid ethanolamine* or Palmitoyl ethanolamide* or Palmitoylethanolamide* or Palmitoylethanolamine* or n-palmitoylethanolamine* or n palmitoylethanolamine* or palmidrol* or Hydroxyethylpalmitamide* or Heptadecanoyl ethanolamide* or Heptadecanoylethanolamide * or Heptadecanoylethanolamine* or n-heptadecanoylethanolamine* or n heptadecanoylethanolamine* or Heptadecanoyl ethanolamine* or Stearoyl ethanolamide* or Stearoylethanolamide* or Stearoylethanolamine* or n-stearoylethanolamine* or n Stearoyl ethanolamine* or Stearoyl ethanolamine* or Oleoyl ethanolamide* or Oleoylethanolamide* or Oleoylethanolamine* or n-oleoylethanolamine* or n Oleoylethanolamine* or Oleoylethanolamine* or Alpha-linolenoyl ethanolamide* or alpha-linolenoylethanolamine* or n-alpha-linolenoyl ethanolamine* or Alpha-linolenoyl ethanolamine* or n alpha-linolenoyl ethanolamine* or alpha-linolenoyl ethanolamine* or Linoleoyl ethanolamide* or Linoleoylethanolamide* or Linoleoylethanolamine* or n-Linoleoylethanolamine* or n Linoleoylethanolamine* or Linoleoyl ethanolamine* or Anandamide* or n-arachidonoylethanolamine* or arachidonoyl ethanolamide* or arachidonoylethanolamide* or Eicosadienoyl ethanolamide* or Eicosadienoylethanolamine* or n arachidonoylethanolamine* or Arachidonoyl ethanolamine* or arachidonoyl glycerol* or Docosatetraenoyl ethanolamide* or Docosatetraenoylethanolamide* or Docosatetraenoylethanolamine* or n-docosatetraenoylethanolamine* or n docosatetraenoylethanolamine* or Docosatetraenoyl ethanolamine* or Eicosapentanoyl ethanolamide* or Eicosapentanoylethanolamide* or Eicosapentanoylethanolamine* or n-Eicosapentanoylethanolamine* or n Eicosapentanoylethanolamine* or Eicosapentanoyl ethanolamine* or Docosapentaenoyl ethanolamide* or Docosapentaenoylethanolamide* or Docosapentaenoylethanolamine* or n-docosapentaenoylethanolamine* or n docosapentaenoylethanolamine* or docosapentaenoyl ethanolamine* or Docosahexanoylethanolamide* or Docosahexanoyl ethanolamide* or Docosahexanoylethanolamine* or n-docosahexanoylethanolamine* or n docosahexanoylethanolamine* or docosahexanoyl ethanolamine* or synaptamide or synaptamine* or dihomo-gama-linolenoyl ethanolamide* or linolenoyl ethanolamide* or inolenoyl ethanolamimine* or dihomo-gama-linolenoyl ethanolamine* or dihomo gamma linolenoyl ethanolamide* or fatty acid amide hydrolase* or faah*).ab,ti,kf.

42. (Phospholipase A2 or PLA2 or sPLA2 or cPLA2 or ipla2 or lp-PLA2 or pla2g4a or pla2g6 or Phosphoinositide phospholipase C or Phospholipase C or PLC or Phospholipase* or Lecithinase* or Lypoxygenase*or LOX*or Arachidonate 5-lipoxygenase* or ALOX5 or 5-lipoxygenase* or 5-LOX or 5-LO or 5LOX or 5LO or 5 LOX or Arachidonate 12-lipoxygenase* or ALOX12 or 12-lipoxygenase or 12-LOX or 12-LO or 12LOX or 12LO or 12 LOX or 15-LOX or 15LOX or 15 LOX or 15-LO or 15LO or Arachidonate 15-lipoxygenase-1 or ALOX15 or 15-lipoxygenase-1 or erythrocyte type 15-lipoxygenase* or 15-lipoxygenase* or reticulocyte type 15-lipoxygenase* or 15-lipoxygenase, reticulocyte type or 15-LO-1 or 15-LOX-1 or 15LO1 or 15LOX1 or Arachidonate 15-lipoxygenase* or Arachidonate 15-lipoxygenase type II or ALOX15B or 15-lipoxygenase-2 15-LOX-2 or 15-LOX-2 or Arachidonate 12-lipoxygenase* or 12R type ALOX12B or 12R-lipoxygenase or 12R-LOX or 12R-LO or platelet-type lipoxygenase 12 or ALOX12 or ALOX15 or ALOX5 or leukocyte-type arachidonate 15-lipoxygenase or Cytochrome p450 or CYP* or cyclooxygenase* or COX2 or COX-2 or COX1 or COX-1 or COX* or prostaglandin-endoperoxide synthase* or Prostaglandin E Synthase 2 or Prostaglandin-E Synthase or PTGS1 or PTGES2 or PGES* or mPGES or cPGES or PTGES* or (Prostaglandin ADJ3 Synthase*) or (5-lypoxygenase activating ADJ1 protein) or FLAP or epoxygenase* or CYP epoxygenase* or Cytochrome P450 omega hydroxylase* or cytochrome P450 omega-hydroxylase* or CYP450 omega hydroxylase* or CYP450 omega-hydroxylase* or CYP omega hydroxylase* or CYP omega-hydroxylase* or fatty acid omega hydroxylase* or cytochrome P450 monooxygenase* or fatty acid monooxygenase* or docosahexaenoic acid omega-hydroxylase* or docosahexaenoate,NADPH:O2 oxidoreductase* or P450 omega-hydroxylase* or thromboxane A synthase* or thromboxane-A synthase* or TBXAS1 or acyltransferase lysolecithin-lysolecithin or l2, lysophospholipase or lecithinase b or lysolecithin lysolecithin acyltransferase* or lysolecithin-lysolecithin acyltransferase or lysophospholipase* or lysophospholipase a or lysophospholipase a1 or lysophospholipase c or lysophospholipase l2 or phospholipase b or phospholipase c or phospholipase*, type c or type c phospholipase* or arachidonate lipoxygenase* or arachidonic acid lipoxygenase* or lipoxygenase* arachidonic acid or lipoxygenase* or Elovanoid*).ti,ab,kf.

43. 26 or 27 or 28 or 29 or 30 or 31 or 32 or 33 or 34 or 35 or 36 or 37 or 38 or 39 or 40 or 41 or 42

44. 16 and 25 and 43

**APPENDIX 2 – SEARCH WITH EMBASE APPENDIX**

1. brain*.ab,ti,kw.

2. cereb*.ab,ti,kw.

3. central nervous system.ab,ti,kw.

4. CNS.ab,ti,kw.

5. ((cerebr* adj3 tissue*) or (brain adj3 tissue*) or (cerebral adj3 sample*) or (brain adj3 sample*)).ab,ti,kw.

6. exp central nervous system/ or exp brain/ or exp brain cell/ or exp brain membrane/ or exp brain nerve cell/ or exp brain nucleus/ or exp brain region/ or exp brain stem/ or exp forebrain/ or exp gray matter/ or exp mossy fiber/ or exp white matter/ or exp brain tissue/ or exp brain histology/ or exp brain ganglion/ or exp brain fornix/ or exp brain third ventricle/ or exp brain cortex/ or exp brain biopsy/ or exp brain cortex slice/ or exp brain cortex layer/ or exp lipid brain level/ or exp brain fourth ventricle/

7. exp blood-brain barrier/ or exp amygdala neuron/ or exp cerebellum cell/ or exp gracile nucleus cell/ or exp pituicyte/ or exp superior olivary nucleus nerve cell/ or exp suprachiasmatic nucleus neuron/ or exp trigeminus nucleus nerve cell/ or exp vagus nucleus nerve cell/ or exp diencephalon/ or exp anteroventral third ventricle region/ or exp brain interventricular foramen/ or exp epithalamus/ or exp hypothalamus/ or exp medial preoptic area/ or exp median eminence/ or exp optic chiasm/ or exp stria terminalis/ or exp subcommissural organ/ or exp subthalamus/ or exp thalamus/ or exp telencephalon**/** or exp allocortex/ or exp archicortex/ or exp basal forebrain/ or exp brain commissure/ or exp brain cortex/ or exp brain fornix/ or exp corpus callosum/ or exp "diagonal band of broca"/ or exp hemisphere/ or exp lateral brain ventricle/ or exp meynert basal nucleus/ or exp mitral cell/ or exp olfactory cortex/ or exp paleocortex/ or exp peduncular ansa/ or exp rhinencephalon/ or exp septal area/ or exp septum nucleus/ or exp septum pellucidum/ or exp stria terminalis/ or exp subcallosal gyrus/ or exp subcortex/ or exp substantia innominata/ or exp subventricular zone/ or exp cerebellar peduncle/ or exp cuneate nucleus/ or exp geniculate nucleus/ or exp gracile nucleus/ or exp lateral reticular nucleus/ or exp locus ceruleus/ or exp magnocellular nucleus/ or exp median raphe nucleus/ or exp medulla oblongata/ or exp mesencephalon/ or exp parabrachial nucleus/ or exp pons/ or exp respiration center/ or exp reticular formation/ or exp trigeminal nucleus/ or exp vagus nerve nucleus/ or exp cerebellar mossy fiber/ or exp hippocampal mossy fiber/ or exp arcuate fasciculus/ or exp capsula interna/ or exp "corona radiata (brain)"/ or exp external capsule/ or exp extreme capsule/ or exp inferior longitudinal fasciculus/ or exp occipitofrontal fasciculus/ or exp superior longitudinal fasciculus/ or exp uncinate fasciculus/ or exp dopaminergic system/ or exp gabaergic system/ or exp meninx

8. (Brain* or hippocamp* or encephalon or Blood Brain Barrier or hemato-encephalic barriers or barriers brain-blood or hemato encephalic barrier or barriers hemato-encephalic or barrier hemato-encephalic or hemato-encephalic barrier or truncus cerebrus or truncus cerebri or cerebri truncus or brainstems or cerebrus truncus or Mesencephalon or mesencephalon or mesencephalons or midbrains or midbrain or Cerebral Peduncle or Cerebral Crus or Substantia Nigra or nigras substantia or nigra substantia or substantia nigras or Pars Compacta or Pars Reticulata or Tegmentum Mesencephali or midbrain trigeminal nucleus or nucleus peripeduncular or annulari nucleus or nervi trochlearis nucleus or midbrain tegmentum or mesencephalus tegmentum or tegmental nucleus ventral or mesencephalic tegmentums or midbrain tegmentums or trigeminal nucleus mesencephalic or tegmentums midbrain or trochlearis nucleus nervi or nucleus annularis or trigeminal nucleus midbrain or nucleus annular or mesencephali tegmentum or darkshevichs nucleus or tegmentums mesencephalic or ventral tegmental nucleus or mesencephalic trigeminal nucleus or nervi trochleari nucleus or nucleus darkshevich's or darkschewitsch nucleus or tegmentum of midbrain or nucleus annulari or cajal interstitial nucleus or mesencephalic tegmentum or nuclei accessory oculomotor or trochlear nucleus or annularis nucleus or nucleus mesencephalic trigeminal or nucleus of darkschewitschor peripeduncular nucleus or oculomotor nuclei accessory or tegmentum midbrain or tegmentum mesencephali or nucleus nervi trochlearis or darkshevich nucleus or nucleus tractus mesencephalici nervi trigemini or interstitial nucleus of cajal or Cerebral Aqueduct or ducts mesencephalic or mesencephalic ducts or aqueduct mesencephalic or sylvian aqueducts or duct mesencephalic or sylvius aqueduct or cerebrus aqueductus or aqueductus cerebrus or cerebral aqueduct or aqueduct sylvian or aqueduct of sylvius or mesencephalic duct or cerebral aqueducts or aqueducts sylvian or aqueduct cerebral or sylvian aqueduct or aqueductus cerebri or aqueducts mesencephalic or cerebri aqueductus or mesencephalic aqueduct or Midbrain Reticular Formation or Pedunculopontine Tegmental Nucleus or nucleus tegmentalis pedunculopontinus or nucleus pedunculopontine tegmental or tegmental nucleus pedunculopontine or pedunculopontine tegmental nucleus or Oculomotor Nuclear Complex or Edinger-Westphal Nucleus or Periaqueductal Grey or greys central periaqueductal or griseum centrales or central grey substance of midbrain or periaqueductal greys central or grey matter periaqueductal or grey central periaqueductal or substantia grisea centralis or periaqueductal grey matter or central periaqueductal grey or grisea centralis substantia or periaqueductal grey or centrale mesencephali griseumor centrale mesencephalus griseum or centrale griseum or grey matters periaqueductalor centrales griseum or periaqueductal grey central or substantia grisea centralis mesencephali or mesencephalus griseum central or midbrain central grey or central grey mesencephalic or central periaqueductal greys or central grey midbrain or griseum centrale mesencephali or Raphe Nuclei or nucleus incertus or nucleus superior central ornuclei raphe ornucleus interfascicular or superior central nucleus or raphe nuclei or interfascicular nucleus or raphe nucleus or incertus nucleus or central nucleus superior or rostral linear nucleus of the raphe or caudal linear nucleus of the raphe or rostral linear nucleus of raphe or nucleus rapheor Dorsal Raphe Nucleus or Interpeduncular Nucleus or Midbrain Raphe Nuclei or Red Nucleus or nucleus ruber or red nucleus or nucleus red or Ventral Tegmental Area or tegmentalis ventralis area or tegmentalis ventrali area or area tegmentalis ventralis or ventral tegmental area of tsai or ventral tegmental area or tegmental area ventral or area tegmentalis ventrali).ti,ab,kw.

9. (Locus Coeruleus or coeruleus complex locus or complices locus coeruleus or locus caeruleus or complex locus ceruleus or complices locus ceruleus or coeruleus complices locus or ceruleus complex locus or locus ceruleus complex or complex locus coeruleus or locus ceruleus complices or locus ceruleus or nucleus pigmentosus pontis or locus coeruleus complices or pontis nucleus pigmentosus or ceruleus complices locus or locus coeruleus or locus coeruleus complex or Tectum Mesencephali or corpora quadrigemina or inferior colliculus commissures or colliculus commissures superior or colliculus commissures inferior or quadrigeminal plates or superior colliculus commissure or plate quadrigeminal or commissure of superior colliculus or quadrigemina corpora or commissure of inferior colliculus or lamina quadrigemina or inferior colliculus commissure or colliculus commissure inferior or quadrigeminal plate or tectum mesencephalus or mesencephalus tectum or plates quadrigeminal or quadrigemina lamina or colliculus commissure superior or Inferior Colliculi or colliculi inferior or inferior colliculi or inferiors colliculus or posterior colliculus or brachial nucleus of the inferior colliculus or caudal colliculus or colliculus inferiors or colliculus caudal or inferior colliculus or colliculus posterior or colliculus inferior or Subcommissural Organ or subcommissural organs or subcommissural organ or organs subcommissural or organ subcommissural or Superior Colliculi or mammalian optic lobesor optic lobe mammalian or optic tectums or superior colliculi or optic tectum or anterior colliculus or colliculus superior or human optic lobes or superior colliculus or optic lobes human or optic lobes mammalian or optic lobe human or colliculi superior or tectum optic or tectums optic or mammalian optic lobe or human optic lobe or colliculus anterior or Reticular Formation or formations reticular or reticular formation or reticular formations or formation reticular or edunculopontine Tegmental Nucleus or nucleus tegmentalis pedunculopontinus or nucleus pedunculopontine tegmental or tegmental nucleus pedunculopontine or pedunculopontine tegmental nucleus or Respiratory Center or centers respiratory or respiratory centers or center respiratory or respiratory center or hombencephalon or hind brains or brains hind or rhombencephalons or hindbrain or hindbrains or brain hind or rhombencephalon or hind brain or Medulla Oblongata or medulla oblongata or nucleus ambiguous or arcuate nucleus-1 or accessory cuneate nucleus or nucleus external cuneate or cuneate nucleus accessory or nucleus ambiguous or medulla oblongatas or arcuate nucleus of the medulla or cuneate nucleus lateralor nucleus lateral cuneate or ambiguous nucleus or cuneate nucleus external or arcuate nucleus 1 or external cuneate nucleus or ambiguus nucleus or arcuate nucleus-1s or lateral cuneate nucleus or Area Postrema or area postremas or trigger zone chemoreceptor or chemoreceptor trigger zone or chemoreceptor trigger zones or trigger zones chemoreceptor or zone chemoreceptor trigger or postrema area or zones chemoreceptor trigger or area postrema or Olivary Nucleus ornucleus basalis olivary or nucleus olivary or basalis olivary nucleus or nucleus olivary basal or olivary basal nucleus or basal nucleus olivary or olivary nucleus or Raphe nuclei or nucleus incertus or nucleus superior central or nuclei raphe or nucleus interfascicular or superior central nucleus or raphe nuclei or interfascicular nucleus or raphe nucleus or incertus nucleus or central nucleus superior or rostral linear nucleus of the raphe or caudal linear nucleus of the raphe or rostral linear nucleus of raphe or nucleus raphe or Nucleus Raphe Obscurus or Nucleus Raphe Pallidus or Solitary Nucleusor solitary nuclear complices or nucleus of tractus solitaries or complex solitary nuclear or tractus solitarii nuclei or nucleus solitaries or solitarius nucleus tractus or tractus solitarius nucleus or solitarius nuclei tractus or solitary tract nucleus or nucleus solitary tract or solitary nuclear complex or tractus solitarius nuclei or nuclear complices solitary or nuclei tractus solitarii or solitary nucleus ornucleus solitaryor nucleus of the solitary tract or nuclear complex solitary or complices solitary nuclear or nucleus of solitary tract or nucleus tractus solitaries or Trigeminal Nucleus, Spinal or trigeminal nucleus spinal or nucleus spinal trigeminal or spinal trigeminal nucleus or Trigeminal Caudal Nucleus or caudal nucleus trigeminal or nucleus trigeminal caudal or trigeminal caudal nucleus or Metencephalon or Cerebellumor corpus cerebellus or parencephalons or cerebellus corpus or cerebellum or cerebellums or corpus cerebelli or parencephalon or cerebelli corpus or Cerebellar Cortex or cerebelli cortex or cortex cerebellus or cerebellar cortex or cortex cerebelli orcerebellus cortex or cortex cerebellar or Cerebellar Vermis or Purkinje Cells or purkinje cells or cells purkinje or Cerebellar Nuclei or nucleus dentatus or Cerebellopontine Angle or central nucleus or central nucleus or interposed nucleus anterior or nucleus globosus or medial cerebellar nucleus or emboliformis nucleus or nuclei cerebellar or intracerebellar nuclei or nucleus fastigii or nucleus fastigial or fastigii nucleus or central nuclei or nuclei central or deep cerebellar nucleus or intracerebellar nucleus or nucleus fastigial cerebellar or nucleus anterior interposed or nucleus intracerebellar oranterior interposed nucleus or nucleus anterior interpositus or nucleus medial cerebellar or nuclei intracerebellar or nucleus dentate or dentate nucleus or interpositus nucleus anterior or globosus nucleus orcerebellar nucleus deep or nucleus central or nucleus cerebellar or cerebellar nuclei deep or nucleus dentate cerebellar or anterior interpositus nucleus or cerebellar nucleus medial or cerebellar nuclei or fastigial cerebellar nucleus or Pons or pons or varolii ponsor pontes or pons varolius or varolius pons or pons varolii or ponte or Barrington's Nucleusor Cochlear Nucleus or cochlear nucleus or nuclei cochlear or cochlear nuclei or nucleus cochlear or Kolliker-Fuse Nucleus or Middle Cerebellar Peduncle or Pontine Tegmentum or Abducens Nucleus or Facial Nucleus or Parabrachial Nucleus or Nucleus Raphe Magnus or Superior Olivary Complex or Trapezoid Body or Trigeminal Motor Nucleus or Vestibular Nuclei or schwalbes nucleus or nucleus schwalbe or vestibular nuclei or vestibular nucleus medial or nuclei vestibular or schwalbe's nucleus or nucleus schwalbe's or medial vestibular nucleus or schwalbe nucleus or nucleus medial vestibular or Vestibular Nucleus, Lateral or deiters nucleus or deiter's nucleus or nucleus of deiters or lateral vestibular nucleus or nucleus lateral vestibular or vestibularis laterali nucleus or nucleus vestibularis laterali or vestibular nucleus lateral or vestibularis magnocellulari nucleus or vestibularis magnocellularis nucleus or deiter nucleus or nucleus vestibularis magnocellularis or nucleus vestibularis magnocellulari or nucleus deiter or nucleus vestibularis lateralis or vestibularis lateralis nucleus or nucleus deiter's or Tectospinal Fibers or Trigeminal Nuclei or trigeminal nucleus or trigeminal nuclear complices or trigeminal nuclear complex or nuclei trigeminal or trigeminal nuclei or nucleus trigeminal or nuclear complices trigeminal or nuclear complex trigeminal or Grey Matter or grey matter or grey matters cerebellar or grey matter cerebellar or matters grey or matter cerebellar grey or grey matter cerebellar or cerebellar grey matters or grey matters or matter cerebellar grey or cerebellar grey matter or matters grey or cerebellar grey matters or matters cerebellar grey or grey matters cerebellar or matters cerebellar grey or grey matter or cerebellar grey matter or matter grey or matter grey or White Matter or white matter cerebellar or matter cerebellar white or matter white or matters cerebellar white or white matters cerebellar or cerebellar white matters or cerebellar white matter or matters white or white matter or white matters or Cerebral Ventricles or cerebral ventricle or cerebral ventriclesor monro foramen or ventricles cerebral or foramen of monro or cerebral ventricular system or ventricle cerebral or Choroid Plexus or choroideus plexus or plexus choroideusor choroid plexus or chorioid plexus or plexus chorioid or plexus choroid or Ependyma or ependymal or ependymas or Fourth Ventricle or ventricolo quarto or ventricles fourth or ventricle fourth or 4th ventricle or quarto ventricolos or ventricle 4th or ventricles 4th or fourth ventricle or ventricolos quarto or fourth ventricles or 4th ventricles or quarto ventricolo or Lateral Ventricles or lateral ventricle orsubventricular zones or lateral ventricles or ventricle lateral or zone subventricular or ventricles lateral or subventricular zone or zones subventricular or Septum Pellucidum or septum supracommissural or pelusidum septum or septum pellucidum or lucidums septum or supracommissural septum or pellucidum septum or septum pelusidums or septum pelusidum or pelusidums septum or septum lucidums or supracommissural septums or septums supracommissural or lucidum septum or septum lucidum or Third Ventricle or 3rd ventricle or ventricles third or ventricles 3rd or third ventricle or ventricle 3rd or 3rd ventricles or third ventricles or ventricle third or Limbic System or limbic system or system limbic or systems limbic or limbic systems or Amygdala or amygdaloid bodies or corpus amygdaloideums or nucleus intercalated amygdaloid or corpus amygdaloideum or amygdaloid body or complex amygdaloid nuclear or amygdaloid nuclear complices or amygdaloid nucleusor intercalata massa or amygdaloideums corpus or intercalatas massa or amygdaloid nucleus intercalated or nuclear complices amygdaloid or archistriatums or amygdala or massa intercalates or nucleus amygdaloid or amygdaloideum corpus or amygdalae nucleus or nuclear complex amygdaloid or archistriatum or nucleus amygdalae or amygdaloid nuclear complex or Basolateral Nuclear Complex or Central Amygdaloid Nucleus or Corticomedial Nuclear Complex or Periamygdaloid Cortex or epithalamus or Habenula or commissure habenular or habenula complex or habenulas or complices habenula or nucleus habenularis or habenular commissures or complex habenula or habenula complices or nucleus habenular or nucleus habenulari or commissures habenular or habenula or habenularums commissura or commissura habenularum or habenularis nucleus or habenular nuclei or commissura habenularums or nuclei habenular or habenulari nucleus or habenular nucleus or Pineal Gland or pineales corpus or body pineal or glands pineal or pineal glands or pineal body or cerebri epiphysis or corpus pineales or gland pineal or pineale corpus or bodies pineal or corpus pineale or pineal gland or pineal bodies or epiphysis cerebri).ti,ab,kw.

10. (Hippocampus or hippocampal formation or propers hippocampus or hippocampus propers or formations hippocampal or horn ammon's or schaffer collateral or ammon horn or hippocampus or horn ammon or cornu ammonis or hippocampus proper or proper hippocampus or collaterals schaffer or formation hippocampal or hippocampal formations or subiculum or subiculums or ammon's horn or CA1 Region, Hippocampal or regio superior of hippocampus or field hippocampus ca1 or ca1 stratum radiatum or stratum radiatum ca1 or hippocampal sector ca1 or hippocampus ca1 field or hippocampus regio superior or ca1 stratum radiatums or sector ca1 hippocampal or ca1 field hippocampus or radiatums ca1 stratum or stratum radiatums ca1 or ca1 hippocampal sector or ca1 pyramidal cell area or ca1 region hippocampal or ca1 pyramidal cell layer or ca1 stratum pyramidale or stratum pyramidale ca1 or cornu ammonis 1 area or radiatum ca1 stratum or CA2 Region, Hippocampal or ca2 stratum pyramidale or radiatums ca2 stratum or cornu ammonis 2 area or ca2 field hippocampus or stratum pyramidale ca2 or stratum radiatum ca2 or ca2 stratum radiatums or radiatum ca2 stratum or sector ca2 hippocampal or region hippocampal ca2 or ca2 field of hippocampus or stratum radiatums ca2 or ca2 region hippocampal or hippocampal sector ca2 or hippocampal ca2 region or hippocampus ca2 field or ca2 pyramidal cell layer or field hippocampus ca2 or ca2 pyramidal cell area or CA3 Region, Hippocampal or stratum lucidum ca3 or ca3 stratum lucidum or stratum lucidums ca3 or lucidum ca3 stratum or ca3 region hippocampal or ca3 pyramidal cell area or hippocampus ca3 field or ca3 hippocampal sector or sector ca3 hippocampal or ca3 stratum radiatum or ca3 stratum lucidums or hippocampal ca3 regions or cornu ammonis 3 area or ca3 field of hippocampus or radiatum ca3 stratum or field hippocampus ca3 or stratum radiatums ca3 or ca3 pyramidal cell layer or lucidums ca3 stratum or region hippocampal ca3 or radiatums ca3 stratum or ca3 stratum pyramidale or ca3 field hippocampus or Dentate Gyrus or ca4 region hippocampal or dentate fascia or cornu ammonis 4 area or hilus gyri dentate or ca4 field of hippocampal formation or ca4 hippocampal sector or gyrus dentate or sector ca4 hippocampal or hippocampal ca4 region or area dentata or region hippocampal ca4 or dentata area or field hippocampal ca4 or gyrus dentatus or hilus of the fascia dentata or hilus of dentate gyrus or dentate gyrus or area dentatas or dentata fascia or hippocampal sector ca4 or hippocampal ca4 field or ca4 of lorente de no or Mossy Fibers, Hippocampal or hippocampal mossy fiber or mossy fibers hippocampal or hippocampal mossy fibers or mossy fiber hippocampal or Fornix, Brain or hippocampal commissure or hippocampal commissures or commissures dorsal hippocampal or fornix commissures or fornices or brain fimbrias or fornical commissures or fornical commissure or fornix or hippocampal commissures dorsal or commissures hippocampal or fornix-fimbria or hippocampal commissure dorsal or fimbria or fornix fimbria or fimbria of hippocampus or brain fornices or dorsal hippocampal commissure or commissure fornical or commissure dorsal hippocampal or commissure of fornix or commissures fornical or commissure hippocampal or fornix commissure or fimbria-fornix or fimbria fornix or fimbria brain or hippocampus fimbrias or hippocampus fimbria or brain fimbria or Hypothalamus or preoptico-hypothalamic areas or preoptico hypothalamic area or lamina terminalis or hypothalamus or areas preoptico-hypothalamic or area preoptico-hypothalamic or preoptico-hypothalamic area or Hypothalamic Area, Lateral or area hypothalamica laterali or hypothalamica laterali area or hypothalami area lateralis or lateralis area hypothalamica or hypothalamus area lateralis or laterali area hypothalamica or areas lateral hypothalamic or lateralis hypothalami area or lateral hypothalamic areas or accessory nucleus of the ventral horn or lateral tuberal nuclei or tuberal nucleus lateral or lateral hypothalamus or area hypothalamica lateralis or hypothalamus lateral or tuberomammillary nucleus or hypothalamic area lateral or nucleus tuberomammillary or nuclei lateral tuberal or nucleus lateral hypothalamic or lateralis hypothalamus area or area lateral hypothalamic or hypothalamic nucleus lateral or area lateralis hypothalamus or nucleus lateral tuberal or Hypothalamus, Anterior or commissures anterior hypothalamic or anterior hypothalamic decussation of ganser or hypothalamic commissures anterior or anterior hypothalamic commissures or commissure anterior hypothalamic or periventricular nucleus anteroventral or nucleus anteroventral periventricular or anterior hypothalamic commissure or hypothalamic commissure anterior or hypothalamus anterior or hypothalamus supraoptic or anteroventral periventricular nucleus or anterior hypothalamus or supraoptic hypothalamus or Anterior Hypothalamic Nucleus or areas anterior hypothalamic or hypothalamic area anterior or nucleus anterior hypothalamic or anterior hypothalamic nucleus or hypothalami nucleus anterior or hypothalamic areas anterior or anterior hypothalami nucleus or anterior hypothalamic area or area anterior hypothalamic or nucleus anterior hypothalamus or hypothalamus nucleus anterior or anterior hypothalamic areas or anterior hypothalamus nucleus or nucleus anterior hypothalami or hypothalamic nucleus anterior).ab,ti,kw.

11. (Organum Vasculosum or Paraventricular Hypothalamic Nucleus or hypothalamic paraventricular nucleus or paraventricular hypothalamic nucleus or nucleus paraventricular hypothalamic or nucleus hypothalamic paraventricular or nucleus paraventricular or paraventricular nucleus or hypothalamic nucleus paraventricular or paraventricular nucleus hypothalamic or Preoptic Area or area medial preoptic or preoptic area medial or preoptic nucleus or nuclei preoptic or lateral preoptic area or preoptic areas lateral or area preoptic or areas medial preoptic or area lateral preoptic or preoptic areas medial or lateral preoptic areas or preoptica area or nucleus preoptic or medial preoptic areas or areas lateral preoptic or area preoptica or areas preoptic or preoptic nuclei or medial preoptic area or preoptic area or preoptic areas or Suprachiasmatic Nucleus or nucleus suprachiasmatic or suprachiasmatic nucleus or Supraoptic Nucleus or hypothalamus supraoptic nucleus or supraoptic group accessory or accessory supraoptic groups or supraoptic nucleus of hypothalamus or supraopticus nucleus or groups accessory supraoptic or nucleus supraoptic or group accessory supraoptic or accessory supraoptic group or nucleus supraopticus or supraoptic groups accessory or supraoptic nucleus or Hypothalamus, Middle or regions intermediate hypothalamic or hypothalamic region intermediate or region intermediate hypothalamic or middle hypothalamus or hypothalamus medial or hypothalamic regions intermediate or intermediate hypothalamic regions or intermediate hypothalamic region or hypothalamus middle or medial hypothalamus or Arcuate Nucleus of Hypothalamus or nucleus arcuate or arcuate nucleus or hypothalamus arcuate nucleus or nucleus infundibular or infundibular nucleus or arcuate nucleus of hypothalamus or Dorsomedial Hypothalamic Nucleus or nucleus arcuate or arcuate nucleus or hypothalamus arcuate nucleus or nucleus infundibular or infundibular nucleus or arcuate nucleus of hypothalamus or Hypothalamo-Hypophyseal System or hypothalamic pituitary unit or hypothalamo hypophyseal system or hypothalamo-hypophyseal system or hypothalamic-pituitary unit or Median Eminence or eminentia medianas or median eminence or eminences medial or eminence medial or medial eminences or medianas eminentia or eminentia mediana or mediana eminentia or eminence median or medial eminence or Pituitary Gland or hypophyseal infundibulum or infundibular hypothalamus or pituitary glands or infundibulum or stalk infundibular or hypothalamus infundibular or infundibulums or pituitary stalks or pituitary gland or hypophysis or pituitary stalk or infundibular stem or stalks infundibular or glands pituitary or hypophysis cerebri or hypophyseal stalks or cerebri hypophysis orstalk hypophyseal or infundibular stalk or infundibular stalks or hypophysis cerebrus or hypophyseal stalk or Pituitary Gland, Anterior or lobus anteriors or anterior lobe of pituitary or anterior lobus or pituitary pars distalis or anterior pituitary glands or anteriors lobus or lobus anterior or pituitary gland anterior or adenohypophyses or pituitary glands anterior or adenohypophysis or pituitary anterior lobe or anterior pituitary gland or pars distalis of pituitary or Corticotrophs or Gonadotrophs or lh producing cells or lh-secreting cells or fsh cells or gonadotrophs or lh cell or fsh-secreting cellsor fsh secreting cells or fsh-producing cells or fsh-producing cell or fsh cell or lh-producing cells or fsh producing cells or lh secreting cells or fsh-secreting cell or gonadotroph or lh-producing cell or lh-secreting cell or lh cell or Lactotrophs or pituitary prolactin-secreting cells or lactotrophs or pituitary prolactin cell or prolactin-secreting cell pituitary or prolactin-secreting cells pituitary or lactotroph or prolactin cell pituitary or prolactin cells pituitary or pituitary prolactin cells or pituitary prolactin-secreting cell or pituitary prolactin secreting cells or Somatotrophs or gh cell pituitary or somatotrophs or gh cells pituitary or pituitary growth hormone-secreting cells or pituitary gh cell or pituitary growth hormone secreting cells or pituitary gh cells or somatotroph or Thyrotrophs or Pituitary Gland, Intermediate or Melanotrophs or Pituitary Gland, Posterior or lobes neural or posterior pituitary glands or neural lobe or pituitary pars nervosa or infundibular processes or infundibular process or process infundibular or neurohypophysis or lobe neural or gland posterior pituitary or pituitary posterior lobe or pars nervosa of pituitary or posterior lobe of pituitary or neural lobes or nervosus lobus or lobus nervosus or pituitary gland posterior or processes infundibular or Tuber Cinereum or cinereums tuber or cinereum tuber or tuber cinereum or tuber cinereums or Ventromedial Hypothalamic Nucleus or nucleus ventromedial hypothalamic or hypothalamic nucleus ventromedial or ventromedial hypothalamic nucleus or Hypothalamus, Posterior or posteriors area hypothalamica or area hypothalamica posterior or mammillary regions or region mammillary or nucleus posterior periventricular or hypothalamic regions posterior or hypothalamus posteriors or mammillary region or posterior area hypothalamica or posterior hypothalamic regions or supramammillary commissures or region posterior hypothalamic or supramammillary commissure or regions posterior hypothalamic or posterior hypothalamus or commissures supramammillary or premammillary nucleus or hypothalamic region posterior or posterior hypothalamic region or commissure supramammillary or hypothalamus posterior or hypothalamica posteriors area or periventricular nucleus posterior or nucleus premammillary or Mammillary Bodies or mammillary bodies ormammillary body or body mammillary or mamillary bodies or body mamillary or bodies mamillary or bodies mammillary or mamillary body or Limbic Lobe or Gyrus Cinguli or gyrus cingular or anterior cingulate gyrus or cingulate gyri posterior or cortex anterior cingulate or posterior cingulate gyrus or cinguli anteriors gyrus or mesial region superior or gyrus cingulate or cingulate cortex anterior or cingulate cortex or superior mesial regions or regions cingulate or cortex posterior cingulate or anterior cingulate cortices or posterior cingulates or cingulate bodies or cingulates anterior or cortices anterior cingulate or posterior cingulate cortices or mesial regions superior or posterior cingulate cortex or regions posterior cingulate or cingulate posterior or posterior cingulate region or region posterior cingulate or body cingulate or cortex cingulate or posterior cingulate regions or cingulate gyrus anterior or cingulate gyrus or cingulate gyrus posterior or cingular gyrus or bodies cingulate or cingulate area or anterior cingulates or area cingulate or cingulate regions or regions superior mesial or ingulates posterior or areas cingulate or cingulate cortices anterior or anterior gyrus cinguli or gyri posterior cingulate or gyrus anterior cingulate or gyrus cinguli anteriors or cinguli anterior gyrus or superior mesial region or anterior cingulate or gyrus cinguli anterior or cingulate anterior or region cingulate or cingulate areas).ti,ab,kw.

12. (Parahippocampal Gyrus or gyrus parahippocampal or gyri parahippocampal or parahippocampal gyri posterior or hippocampal gyrus or gyri posterior parahippocampal or posterior parahippocampal gyrus or gyrus parahippocampalis or parahippocampal gyrus uncus or presubiculums or posterior parahippocampal gyri or gyrus posterior parahippocampal or parahippocampal gyrus posterior or uncus of parahippocampal gyrus or gyri hippocampal or parahippocampal gyrus or presubiculum or gyrus hippocampi or uncus parahippocampal gyrus or gyrus uncus parahippocampal or gyrus hippocampal or parahippocampal gyri or Entorhinal Cortex or area entorhinali or areas entorhinal or entorhinalis area or entorhinal area or area entorhinal or entorhinal cortices or area entorhinalis or cortices entorhinal or entorhinali area or olfactory cortices secondary or secondary olfactory cortex or cortex secondary olfactory or cortices secondary olfactory or entorhinal cortex or olfactory cortex secondary or secondary olfactory cortices or cortex entorhinal or entorhinal areas or Olfactory Pathways or olfactory pathways or pathways olfactory or olfactory pathway or pathway olfactory or Perforant Pathway or pathway perforant or pathways perforant or perforant paths or perforant pathways or perforant pathway or fasciculus perforating or paths perforant or perforant path or perforating fasciculus or path perforant or Septum of Brain or paraterminal body or brain septums or brain septum or septum of brain or paraterminal bodies or area septal or bodies paraterminal or body paraterminal or septal area or region septal or septal region or Septal Nuclei or nucleus of the stria terminalis or septi lateralis nucleus or septal nuclear complices or nucleus of anterior commissure or terminali nucleus striae or laterali nucleus septalis or nucleus lateralis septi or nucleus lateralis septus or nuclear complices septal or septofimbrial nucleus or diagonal band nucleus or nucleus septofimbrial or nucleus septi lateralis or laterali nucleus septi or nucleus triangular septal or medial septal nucleus or nucleus of diagonal band or nucleus septalis lateralis or nucleus striae terminali or nuclear complex septal or septum nucleus lateral or lateral septal nucleus or lateralis nucleus septalis or septal nucleus lateral or septalis laterali nucleus or nuclei septal or anterior commissure nucleus or septus nucleus lateralis or septi laterali nucleus or nucleus medial septum or nucleus septalis laterali or triangularis septus nucleus or lateralis nucleus septi or complex septal nuclear or nucleus striae terminalis or nucleus of stria terminalis or septalis lateralis nucleus or dorsal septal nucleus or nucleus triangularis septus or nucleus lateral septumor nucleus triangularis septi or nucleus lateral septal or septal nucleus triangular or terminalis nucleus striae or septi nucleus lateralis or septi nucleus triangularis or triangular septal nucleus or septus nucleus triangularis or nucleus medial septal or complices septal nuclear or lateralis septus nucleus or medial septum nucleus or lateralis septi nucleus or Substantia Innominata or innominata substantia or substantia innominata or Prosencephalon or prosencephalon or forebrains or forebrain or Diencephalon or diencephalon or interbrain or interbrains or Optic Chiasm or chiasmas optic or optic chiasms or decussation optic or chiasma optic or optic chiasm or optic chiasma or optic decussation or opticums chiasma or optic decussations or chiasma opticum or decussations optic or opticum chiasma or optic chiasmas or chiasma opticums or chiasms optic or chiasm optic or Optic Tract or Subthalamus or subthalamus or fasciculus thalamic or field h nucleus or campi forelus nucleus or fasciculus lenticular or field h1 forel's or campi foreli nucleus or enticular fasciculus or forels field h2 or forel field h2 or thalamicus fasciculus or fasciculus thalamicus or thalamic fasciculus or forelus nucleus campi or nucleus of ansa lenticularis or foreli nucleus campi or nucleus campi forelus or nucleus of field h or forels field h1 or forel's field h2 or field h1 of forel or forel field h1 or Entopeduncular Nucleus or Subthalamic Nucleus or nucleus of luys or luys subthalamic nucleus or corpus luysi or luys body or subthalamic nucleus of luys or subthalamicus nucleus or luys nucleus or nucleus subthalamic or luysi corpus or body of luys or nucleus subthalamicus or subthalamic nucleus or Zona Incerta or Thalamus or thalamencephalon or thalamencephalons or thalamus or Thalamic Nuclei or nuclei thalamic or thalamic nuclei or Anterior Thalamic Nuclei or nucleus anterodorsal thalamic or anterior nuclear group or nucleus anteromedial thalamic or nucleus anteroventral thalamic or thalamus anterior nucleus or anterior thalamic nucleus or nucleus anteroventral or anteroventral nucleus or thalamic nucleus anterodorsal or nuclei anterior thalamic or thalamic nuclei anterior or anteromedial nucleus or anteromedial thalamic nucleusor thalamus anterior or nucleus anteromedial or anterodorsal nucleus or anterior thalamus or anterior thalamic nuclei or anterodorsal thalamic nucleus or nucleus anterodorsal or thalamic nucleus anteroventral or Geniculate Bodies or nucleus geniculate or medial geniculate nucleus or geniculate complex medial or geniculatum mediales corpus or bodies geniculate or nucleus lateral geniculate or mediales corpus geniculatum or geniculate bodies medial or mediale corpus geniculatum or geniculate body or geniculatum mediale corpus or geniculate nucleus lateral or geniculate bodies or geniculate bodies lateral or metathalamus or corpus geniculatum mediale or geniculate body lateral or complex medial geniculate or nucleus geniculatus lateralis pars dorsalis or geniculate body medial or geniculate complices medial or geniculate nucleus or complices medial geniculate or medial geniculate body or medial geniculate bodies or geniculate nucleus medial or Intralaminar Thalamic Nuclei or nucleus paracentrali or centrum medianum or paracentrali nucleus or centromedian thalamic nucleus or central lateral nucleus or thalamic nucleus parafascicular or central lateral thalamic nucleus or parafascicular thalamic nucleus or thalamic nucleus intralaminar or nucleus central dorsal or parafascicular nucleus of the thalamus or centromedian nucleus or intralaminar nuclei rostral or intralaminar nuclear group or thalamic nucleus centromedian or parafascicularis nucleus or nucleus central lateral or thalamic nuclei intralaminar or central dorsal thalamic nucleus or interlaminar nuclei of thalamus or rostral intralaminar nuclei or thalamus nucleus parafascicularis or centrum medianums nucleus or medianum centrum or thalamic nucleus paracentral or thalamus reticulate nucleus or nucleus paracentral or nucleus central medial or paracentral thalamic nucleus or median nucleus centre or nuclei intralaminar thalamic or nuclei rostral intralaminar or central medial nucleus or nucleus centrum medianums or nucleus centre median or medianum nucleus centrum or nucleus paracentral thalamic or nucleus centromedian thalamic or nucleus parafascicularis thalamus or nucleus intralaminar thalamic or nucleus centrum medianum or nucleus parafascicularis thalami or parafascicularis thalami nucleus or parafascicularis thalamus nucleus or reticulate nuclei of thalamus or nucleus parafasciculari or centrum medianums or centrum medianum nucleus or paracentralis nucleus or lateral nucleus central or parafascicular nucleus or central medial thalamic nucleus or nucleus centromedian or Lateral Thalamic Nuclei or medial pulvinar nucleus or Pulvinar or anterior pulvinar nucleus).ab,kw,ti.

13. (pulvinar nucleus or nucleus anterior pulvinar or lateral pulvinar nucleus or pulvinar nucleus oral or oral pulvinar nucleus or pulvinar nucleus inferior or pulvinars or pulvinari nucleus or nucleus oral pulvinar or nucleus pulvinar or pulvinar thalami or nucleus pulvinari or nucleus lateral pulvinar or thalami pulvinar or nucleus inferior pulvinar or pulvinaris nucleus or nucleus pulvinaris or pulvinar thalamus or inferior pulvinar nucleus or pulvinar nucleus lateral or Mediodorsal Thalamic Nucleus or medialis dorsali nucleus or medial dorsal thalamic nucleus or mediodorsal nucleus or dorsomedialis thalamus nucleus or nucleus dorsomedial thalamic or nuclei medial thalamic or dorsali nucleus medialis or nucleus mediodorsal or nucleus dorsomedialis thalamus or nucleus medialis dorsali or nucleus mediodorsal thalamic or thalami nucleus dorsomedialis or thalamus nucleus dorsomedialis or thalamic nucleus medial or dorsomedialis thalami nucleus or thalamic nuclei medial or nucleus medial thalamic or mediodorsal thalamic nucleus or nucleus dorsomedial or nucleus medialis dorsalis or thalamic nucleus mediodorsal or dorsal medial nucleus or medialis dorsalis nucleus or nucleus dorsomedialis thalami or medial thalamic nucleus or Midline Thalamic Nuclei or parataenial nucleus or nucleus reunien or rhomboid nucleusor nucleus subfascular or nuclear group midline or paratenial nucleus or rhomboidal nucleus or rhomboid thalamic nucleus or nucleus rhomboid thalamic or reuniens nucleus or subfascular nucleus or thalamus nucleus reuniens or thalami nucleus reuniens or nucleus paraventricular thalamic or reunien nucleus or reuniens thalami nucleusor paraventricular nucleus of thalamus or paraventricular thalamic nucleus or midline thalamic nucleus or thalamic nuclei midline or paratenial thalamic nucleus or thalamic nucleus rhomboid or periventricular nuclei of thalamus or thalamic nucleus reuniens or reuniens thalamus nucleus or thalamus midline nucleus or nucleus paratenial or thalamus paraventricular nucleus or midline thalamic nuclei or thalamic nucleus subfascular or nucleus reuniens thalamus or thalamic nucleus paratenial or nucleus reuniens or nucleus rhomboid or thalamic nucleus paraventricular or midline nuclear group or Posterior Thalamic Nuclei or supergeniculate nucleus or posterior nuclear complicesor nucleus supergeniculateor posterior thalamic nuclei or suprageniculate thalamic nucleus or submedial nucleus or limitans nucleus or thalamic nuclei posterior or nucleus limitan or thalamic nucleus suprageniculate or nucleus submedial or nuclear complices posterior or complices posterior nuclear or posterior nucleus of thalamus or nucleus limitans or nucleus suprageniculate thalamic or posterior thalamic nucleus or posterior thalamic nuclear group or posterior nuclear complex or nuclear complex posterior or thalamus posterior nucleus or Ventral Thalamic Nuclei or posterior nucleus ventral or ventrolateral thalamic nucleus or intermedius nucleus ventralis or ventral posterior nucleus or ventralis intermedius nucleus or ventrobasal complex or ventralis posteromediali nucleus or nuclear mass ventral or group ventral nuclear or ventralis posterolateralis nucleus or nucleus ventral anterior or ventral nuclear groups or laterali nucleus ventralis or nucleus ventral posterolateral or ventralis posterior nucleus or masses ventral nuclear or nucleus ventralis posterolaterali or ventral lateral nucleus or nucleus ventralis intermedius or ventral anterior thalamic nucleus or thalamic nucleus ventral or posterolaterali nucleus ventralis or ventral posteromedial thalamic nucleus or nucleus ventrolateralis thalamus or ventrobasal complices or nucleus ventralis posteromedialis or nuclei ventral thalamic or nucleus ventrolateralis thalami or mass ventral nuclearor ventrolateralis thalami nucleus or ventrolateralis thalamus nucleus or posterolateral nucleus ventral or nuclear group ventral or arcuate nucleus 3 or nucleus ventralis posteriors or ventral posterior thalamic nucleus or ventral posterior medial nucleus or ventral posteroinferior nucleus or posteroinferior nucleus ventral or posteriors nucleus ventralis or arcuate nucleus-3 or thalamus nucleus ventrolateralis or nucleus ventralis or posteromediali or complex ventrobasal or ventral lateral thalamic nucleus or ventral thalamic nuclei or ventral lateral thalamic nuclei or ventral posteromedial nucleus or posteromedialis nucleus ventralis or ventral anterior nucleus or ventral posterolateral nucleus or nuclear masses ventral or ventral posterior inferior thalamic nucleus or thalamic nucleus ventrolateral or ventral nuclear group or thalamus ventrolateral or thalami nucleus ventrolateralis or posteromediali nucleus ventralis or posterolateralis nucleus ventralis or posterior nucleus ventralis or nucleus ventral posteromedial or nucleus ventralis laterali or ventral posterolateral thalamic nucleus or nucleus ventral thalamic or ventralis lateralis nucleus or ventral nuclear mass or ventralis posteriors nucleus or ventralis laterali nucleus or nucleus ventral posterior or ventral thalamic nucleus or ventrolateral thalamus or nucleus ventralis lateralis).ab,ti,kw.

14. (Telencephalon or telencephalon or endbrain or endbrains or Cerebrum or cerebrum or cerebral hemisphere left or cerebral hemisphere right or cerebral hemispheres or right cerebral hemisphere or cerebral hemisphere or left cerebral hemisphere or Basal Ganglia or ganglia basal or nuclei basal or basal ganglia or ganglion basal or basal nuclei or claustrum or Corpus Striatum or lenticular nucleus or nucleus lentiform or lentiformis nucleus or lentiform nucleus or corpus striatum or nucleus lenticular or nucleus lentiformis or lentiform nuclei or striatum corpus or nuclei lentiform or Globus Pallidus or pallidum or paleostriatum or globus pallidus or pallidums or Neostriatum or Caudate Nucleus or nucleus caudatus or caudate nucleus or caudatus nucleusor nucleus caudate or caudatus or High Vocal Center or Putamen or putamens or nucleus putamens or putamens nucleus or putamen nucleus or nucleus putamen or putamen or Ventral Striatum or Nucleus Accumbens or nucleus accumbens or accumbens septus nucleus or accumbens septi nucleusor nucleus accumbens septi or septi nucleus accumbens or accumbens nucleus or septus nucleus accumbens or nucleus accumbens septus or Olfactory Tubercleor Islands of Calleja or Basal Nucleus of Meynert or nucleus basalis of meynert or meynert basal nucleus or nucleus basalis magnocellularis or basal nucleus of meynert or meynert nucleus basalisor Cerebral Cortex or plates cortical or insular cortex or cerebral cortices or archipalliums or paleocortex or allocortices or periallocortices or plate cortical or cerebri cortex or cortices cerebral or paleocortices or cortices insular or insular cortices or cortex insular or periallocortex or archipallium or cortical plates or cortex cerebral or cortex cerebri or reil insula or cortex cerebrus or cortical plate or Frontal Lobe or gyrus anterior centralor central gyrus anterior or lobe frontalor frontal lobeor cortex frontal or gyrus precentralis or frontal eye fieldor supplementary eye field or gyrus precentrali or frontali lobusor precentrali gyrus or frontal lobes or frontal cortex or field supplementary eye or lobes frontal or eye field supplementary or lobus frontali or supplementary eye fields or frontalis lobus or gyrus precentral or eye fields supplementary or eye fields frontal or anterior central gyrus or fields frontal eye or lobus frontalis or Motor Cortex or motor area or primary motor cortex or motor area precentral or strip motor or somatomotor areas or strips motor or motor cortices primary or premotor areas or motor area secondary or cortex precentral motor or motor area somatic or supplementary motor areas or area primary motoror area premotor or secondary motor area or motor cortices secondary or area motor or secondary motor areas or area somatomotor or motor areas or motor cortex secondary or precentral motor areas or cortices secondary motor or area supplementary motor or motor areas supplementary or area precentral motor or cortices primary motor or precentral motor cortices or areas somatic motor or area somatic motor or areas motor or motor cortex precentral or motor areas precentral or motor strips or cortex primary motor or somatomotor area or premotor area or precentral motor cortex or primary motor area or somatic motor area or motor areas somatic or areas premotor or areas somatomotor or areas precentral motor or areas supplementary motor or motor cortex primary or cortex secondary or motor primary motor cortices or motor cortex or motor cortices precentral or motor area supplementary or cortices precentral motor or somatic motor areas or cortex motor or areas secondary motor or Prefrontal Cortex or orbital gyrus).ab,kw,ti.

15. (gyrus orbital or sulcus olfactoryor convolutions superior frontal or orbitofrontal cortices lateral or gyrus frontalis superior or rectal gyrusor cortices ventromedial prefrontal or orbital cortices or cortex orbital or prefrontal cortices ventromedial or inferiors gyrus frontalis or orbital gyri or orbital area or convolution superior frontal or frontalis superiors gyrus or inferior frontal gyrus or gyri orbitofrontal or orbitofrontal regions or frontalis inferiors gyrus or frontal sulcus or prefrontal cortex ventromedial or straight gyrus or cortex lateral orbitofrontal or gyrus frontalis inferior or sulci olfactory or orbital areas or orbitofrontal gyri or area orbital or orbitofrontal region or cortices lateral orbitofrontal or lateral orbitofrontal cortex or superior frontal convolution or cortex orbitofrontal or medial frontal gyrus or gyrus orbitofrontal or gyrus straight or superior frontal gyrus or frontal gyrus medial or ventromedial prefrontal cortex or gyrus rectal or subcallosal area or olfactory sulcus or prefrontal cortex or superior frontal convolutions or sulcus frontal or olfactory sulci or region orbitofrontal or superiors gyrus frontalis or superior gyrus frontalis or gyrus superior frontal ororbitofrontal cortex or frontal gyrus inferior or gyrus frontalis inferiors or cortex ventromedial prefrontal or marginal gyrus or rectus gyrus or orbital cortex or gyrus medial frontal or orbitali gyrus or orbitofrontal gyrus or inferior gyrus frontalis or frontal gyrus superior or gyri orbital or areas orbital or cortex prefrontal or cortices orbital or gyrus rectus or frontalis superior gyrus or lateral orbitofrontal cortices or orbitofrontal cortices or gyrus frontalis superiors or orbitofrontal cortex lateral or gyrus marginal or Broca Area or Neocortex or neocortical molecular layer or neocortices cerebral or isocortex or cerebral neocortices or neopalliumsor corticalis substantiaor multiform layer neocortical or neocortical multiform layer or layer neocortical molecular or cortices neopallial or neopallial cortex or neocortical internal pyramidal layer or molecular layer neocortical or neopallial corticesor cortex neopallial or layers neocortical multiform or neocortex cerebralor molecular layers neocortical or neocortical internal granular layer or neocortical multiform layers or cerebral neocortex or neocortical external pyramidal layer or neocortical molecular layers or isocortices or external granular layer or substantia corticali or corticali substantia or layer neocortical multiformor multiform layers neocortical or Occipital Lobe or occipital cortex or cuneus or gyrus annectant or sulcus lunate or gyrus lingual or gyrus occipitalor calcarine fissures or regions occipital or occipitotemporal gyrus medial or sulcus calcarine or lunate sulcus or occipital lobe or gyrus medial occipitotemporal or cuneate lobule or region occipital or fissures calcarine or calcarinus sulcus or sulcus calcarinus or lobe occipital or lobes occipital or occipital region or gyrus lingualis or occipital gyrus or annectant gyrus or occipital regions or lobules cuneate or occipital sulcus or cortices occipital or calcarine sulcus or fissure calcarine or lingual gyrus or lobule cuneate or cortex cuneus or linguali gyrus or Visual Cortex or primary visual cortices or visual cortex primaries or primaries visual cortex or cortices extrastriate or visual cortices primary or extrastriate cortices or cortex primaries visual or cortex primary visual or striate cortex or cortices primary visual or visual cortex primary or visual cortex or cortex striate or cortex extrastriate or cortex visual or extrastriate cortex or primary visual cortex or Olfactory Cortex or Basal Forebrain or Piriform Cortex or sulcus intraparietal or regions parietal or lobes parietal or paracentral lobules posterior or gyrus supramarginal or precuneus cortices or parietal cortex or gyrus angulari or praecuneus or gyrus angularis or gyrus supramarginali or parietal regions or gyrus prelunate or lobules parietal or lobe parietal or parietal cortices posterior or gyrus supramarginalis or angulari gyrus or supramarginali gyrus or marginal sulcus or posterior parietal cortex or prelunate gyrus or posterior parietal cortices or intraparietal sulcus or angularis gyrus or region parietal or parietal lobules or precuneus or cortex parietal or gyrus angular or precuneus cortex or lobule parietal or parietal lobule or cortices precuneus or posterior paracentral lobule or lobules posterior paracentral or sulcus marginal or posterior paracentral lobules or Parietal Lobe or sulcus intraparietal or regions parietal or lobes parietal or paracentral lobules posterior or gyrus supramarginal or precuneus cortices or parietal cortex or gyrus angulari or praecuneus or gyrus angularis or gyrus supramarginali or parietal regions or gyrus prelunate or lobules parietal or lobe parietal or parietal cortices posterior or gyrus supramarginalis or angulari gyrus or supramarginali gyrus or marginal sulcus or posterior parietal cortex or prelunate gyrus or posterior parietal cortices or intraparietal sulcus or angularis gyrus or region parietal or parietal lobules or precuneus or cortex parietal or gyrus angular or precuneus cortex or lobule parietal or parietal lobule or cortices precuneus or posterior paracentral lobule or lobules posterior paracentral or sulcus marginal or posterior paracentral lobules or omatosensory Cortex or postcentral gyrus or somatosensory cortices primary or somatosensory cortex primary or cortex anterior parietal or cortices anterior parietal or cortices primary somatosensory or anterior parietal cortices or cortex secondary sensory or areas primary somatosensory or primary somatosensory cortices or cortex si or gyrus post central or secondary somatosensory areas or post central gyrus or parietal cortices anterior or gyrus postcentrali or secondary somatosensory cortex or somatosensory cortex or areas secondary somatosensory or somatosensory cortex secondary or area primary somatosensory or postcentralis gyrus or cortices secondary sensory or secondary sensory cortex or primary somatosensory cortex or somatosensory areas secondary or si cortex or primary somatosensory areas or secondary somatosensory cortices or gyrus postcentralis or area secondary somatosensory or primary somatosensory area or cortex primary somatosensory or secondary somatosensory area or secondary sensory cortices or cortices secondary somatosensory or primary somatic sensory area or postcentrali gyrus or gyrus postcentral or Wernicke Area or Sensorimotor Cortex or Auditory Cortex or gyrus transverse temporal or auditory areas temporal or temporal auditory areas or areas auditory or cortex primary auditory or transverse temporal gyrus or cortex auditory or auditory areas or transverse temporal gyri or convolutions heschl's or auditory cortex or auditory area or auditory cortex primary or auditory cortices primary or areas temporal auditory or area auditory or gyri transverse temporal or primary auditory cortices or heschl gyri or heschl convolutions or temporal gyri transverse or auditory area temporal or temporal auditory area or heschls gyri or primary auditory cortex or Temporal Lobe or temporal operculums or superior temporal gyrus or occipito-temporal gyrus lateral or gyrus fusiform or gyrus lateral occipito-temporal or gyrus temporalis superior or occipitotemporal gyrus or horns temporal or inferior horn of lateral ventricle or temporal sulcus or regions temporal or operculums temporal or cortex temporal or lobes temporal or planum polares or temporalis superior gyrus or gyrus superior temporal or horn temporal or lobe temporal or temporal horn or temporal cortices or gyrus lateral occipitotemporal or temporalis superiors gyrus or region temporal or fusiformi gyrus or temporal region or temporal horns or lateral occipito-temporal gyrus or temporal cortex or gyrus temporal or temporal operculum or temporal regions or operculum temporal or cortices temporal or temporal horn of the lateral ventricle or polare planum or fusiformis gyrus or sulcus temporal or polares planum or planum polare or temporal lobe or Diagonal Band of Broca or diagonal band of broca or broca diagonal band or External Capsule or olfactory tracts or olfactory tract lateral or main olfactory bulbs or bulbs main olfactory or bulb olfactory or glomerulus olfactory or lateral olfactory tracts or olfactory tract or bulb main olfactory or olfactory bulbs or olfactory bulb main or accessory olfactory bulb or accessory olfactory bulbs or tracts olfactory or bulbs accessory olfactory or olfactory glomerulus or bulbs olfactory or olfactory bulb accessory or tract olfactory or bulb accessory olfactory or tract lateral olfactory or olfactorius bulbus or olfactory bulb or lateral olfactory tract or Olfactory Bulb or olfactory tracts or olfactory tract lateral or main olfactory bulbs or bulbs main olfactory or bulb olfactory or glomerulus olfactory or lateral olfactory tracts or olfactory tract or bulb main olfactory or olfactory bulbs or olfactory bulb main or accessory olfactory bulb or accessory olfactory bulbs or tracts olfactory or bulbs accessory olfactory or olfactory glomerulus or bulbs olfactory or olfactory bulb accessory or tract olfactory or bulb accessory olfactory or tract lateral olfactory or olfactorius bulbus or olfactory bulb or lateral olfactory tract or Telencephalic Commissures or Anterior Cerebellar Commissure or Corpus Callosum or corpus callosums or commissures neocortical or neocortical commissures or corpus callosum or callosums corpus or interhemispheric commissure or interhemispheric commissures or neocortical commissure or callosum corpus or commissures interhemispheric or commissure interhemispheric or commissure neocortical or Internal Capsule or internal capsules or interna capsula or capsules internal or capsule internal or capsula internas or internal capsule or capsula interna or internas capsula or Myelencephalon).ab,kw,ti.

16. 1 or 2 or 3 or 4 or 5 or 6 or 7 or 8 or 9 or 10 or 11 or 12 or 13 or 14 or 15

17. exp Alzheimer disease/ or exp amyloid plaque/ or exp amyloid beta protein/ or exp amyloid/ or exp amyloid neuropathy/ or exp amyloid protein/ or exp tau protein/ or exp neurofibrillary tangle/ or exp senile plaque/ or exp amyloidosis/ or exp amyloid beta protein/ or exp tauopathy/

18. alzheimer*.ti,ab,kw

19. ase.ti,ab,kw.

20. ((mild adj2 cognitive adj2 impairment) or MCI).ti,ab,kw.

21. (cognitive adj2 decline).ti,ab,kw.

22. AD.ti,ab,kw.

23. ((sporadic ADJ2 Alzheimer*) or (sporadic ADJ2 AD) or (early onset ADJ2 Alzheimer*) or (early onset ADJ2 AD) or familial Alzheimer* or (familial ADJ2 Alzheimer*) or (familial ADJ1 AD) or early onset AD or late onset AD or (late onset ADJ2 Alzheimer*) or (late onset ADJ2 Alzheimer disease) or (late onset ADJ2 AD) or EOAD or LOAD or autopsy confirmed Alzheimer* or (autopsy ADj2 Alzheimer*)).ti,ab,kw.

24. ((dementia ADJ4 Alzheimer*) or (senile dementia ADJ4 Alzheimer*) or (dementia ADJ4 AD) or (senile dementia ADJ4 AD) or Alzheimer sclerosis or Alzheimer syndrome or (focal onset ADJ2 Alzheimer*) or (focal onset ADJ2 AD) or (presenile dementia) or (presenile dementia ADJ4 Alzheimer) or (presenile dementia ADJ4 AD) or beta amyloid plaque* or beta-amyloid plaque* or amyloid beta or beta amyloid or beta-amyloid or Abeta or amyloid deposit* or amyloid plaque* or neuritic plaque* or senile plaque* or Alzheimer* disease associated protein* or AD-AP or ADAP or AD-AP or (beta protein ADJ3 Alzheimer*) or (amyloid beta ADJ3 Alzheimer*) or (amyloid-beta ADJ3 Alzheimer*) or amyloid fibril protein* or amyloid beta peptide* or amyloid beta protein* or amyloid-beta peptide* or amyloid-beta protein* or neurofibrillary tangle*or tau protein* or taupath* or NFT or (tangle* ADJ3 Alzheimer*) or tau or neurofibrillary pathology* or Alzheimer disease-associated neurofibrillary pathology or braak staging or (braak ADJ3 Alzheimer*) or CERAD or (CERAD ADJ3 Alzheimer*)).ti,ab,kw.

25. 17 or 18 or 19 or 20 or 21 or 22 or 23 or 24

26. exp phospholipase/ or exp lysophospholipase/ or exp phospholipase A/ or exp phospholipase A1/ or exp phospholipase A2/ or exp 1 alkyl 2 acetylglycerophosphocholine esterase/ or exp cytosolic phospholipase A2/ or exp calcium independent phospholipase A2/ or exp peroxiredoxin 6/ or exp secretory phospholipase A2/ or exp phospholipase A2 group I/ or exp phospholipase a2 group ia/ or exp phospholipase a2 group ib/ or exp phospholipase a2 group ii/ or exp phospholipase a2 group iii/ or exp phospholipase a2 group iv/ or exp phospholipase a2 group v/ or exp phospholipase a2 group vi/ or exp phospholipase a2 group x/ or exp phospholipase C/ or exp phospholipase D/

27. exp lipoxygenase/ or exp arachidonate lipoxygenase/ or exp arachidonate 5 lipoxygenase/ or exp arachidonate 12 lipoxygenase/ or exp arachidonate 15 lipoxygenase/ or exp prostaglandin synthase/ or exp cyclooxygenase 1/ or exp cyclooxygenase 2/ or exp cyclooxygenase 3/ or exp prostaglandin/ or exp prostaglandin endoperoxide/ or exp prostaglandin A/ or exp prostaglandin B/ or exp prostaglandin C/ or exp prostaglandin D/ or exp prostaglandin E/ or exp prostaglandin F/ or exp prostaglandin G/ or exp prostaglandin H/ or exp icosanoid or exp prostaglandin derivative/ or exp endocannabinoid/

28. exp fatty acid/ or exp oxylipin/ or exp lipid metabolism/ or exp decanoic acid derivative/ or exp arachidic acid/ or exp icosanoid/ or exp arachidonic acid derivative/ or exp arachidonic acid/ or exp hydroxyicosatetraenoic acid/ or exp isoprostane derivative/ or exp leukotriene/ or exp thromboxane/ or exp linoleic acid/ or leukotriene B4 or exp docosahexaenoic acid/ or exp docosapentaenoic acid/ or exp icosapentaenoic acid/ or exp 5,8,11,14 icosatetraynoic acid/ or exp diroleuton/ or exp lipoxin/ or exp icosanoid/ or exp 12 hydroxyicosatetraenoic acid/ or exp 14,15 epoxy 5,8,11 icosatrienoic acid/ or exp 15 hydroperoxyarachidonic acid/ or exp 15 hydroxyicosatetraenoic acid/ or exp 2 arachidonoylglycerol/ or exp 20 hydroxyicosatetraenoic acid/ or exp 5 hydroperoxy 6,8,11,14 icosatetraenoic acid/ or exp 5 hydroxyicosatetraenoic acid/ or exp 5,6 dihydroxy 7,9,11,14 icosatetraenoic acid/ or exp 5,6 epoxy 8,11,14 icosatrienoic acid/ or exp 5,8,11 icosatrienoic acid/ or exp 5,8,11 icosatriynoic acid/ or exp anandamide/ or exp epoxyicosatrienoic acid/ or exp hepoxilin a/ or exp hepoxilin a4/ or exp hepoxilin b/ or exp hepoxilin b4/ or exp hydroxyicosatetraenoic acid/ or exp icomucret/ or exp icosabutate/ or exp icosapentaenoic acid/ or exp icosapentaenoic acid ethyl ester/ or exp icosatetraenoic acid/ or exp icosatrienoic acid/ or exp leukotriene/ or exp lipoxene a/ or exp lipoxene b/ or exp lipoxin/ or exp lipoxin a/ or exp lipoxin b/ or exp methanandamide/ or exp "n (3 furylmethyl)arachidonamide"/ or exp "n (4 hydroxy 2 methylphenyl)arachidonamide"/ or exp "n (4 hydroxyphenyl)arachidonamide"/ or exp nicodicosapent/ or exp prostanoid/

29. ((Arachidonic Acid ADJ 2 metabolite*) or ARA metabolite* or AA metabolite* or Arachidonic Acid or ARA or AA or eicosaHydroxyeicosatetraenoic acid* or eicosa-Hydroxyeicosatetraenoic acid* or Hydroxyeicosatetraenoic acid* or HETE or 5-HETE or 5HETE or 5 HETE or 12-HETE or 12HETE or 12 HETE or 8-HETE or 8HETE or 8 HETE or 9-HETE or 9HETE or 9 HETE or 15-HETE or 15HETE or 15 HETE or 20-HETE or 20HETE or 20 HETE or 19-HETE or 19HETE or 19 HETE or 5-Hydroxyeicosatetraenoic acid* or 15-Hydroxyeicosatetraenoic acid* or 12-Hydroxyeicosatetraenoic acid* or 9-Hydroxyeicosatetraenoic acid* or 8-Hydroxyeicosatetraenoic acid*  20-Hydroxyeicosatetraenoic acid or docosanoid* or Eicosanoid* or 19-Hydroxyeicosatetraenoic acid*).ab,ti,kw.

30. (Leukotriene* or LTA4 or LTB4 or LTC4 or LTD4 or LTE4 or LTX4 or LTA5 or LTB5 or LTC5 or LTD5 or LTE5).ab,ti,kw.

31. (Eoxin* or EXA4 or EXC4 or EXD4 or EXE4 or Prostanoid* or Prostaglandin* or PG* or PGG2 or PGH2 or PGE2 or PGD2 or PGF2* or PGA* or PGA1 or PGA2 or PGB2 or Prostacyclin* or Dihomo-2-series prostanoid* or prostaglandin D1 or prostaglandin D2 or prostaglandin D3 or PGD1 or PGD3 or PGD* or dihomo-prostaglandin* or dihomo-PGD2 or dihomo-PGE2 or dihomo-PGI2 or PGI2 or PGI3 or PGH2 or PGE1 or PGE3 or PGE* or Cyclopentenone prostaglandin* or delta12-PGJ* or 15-deoxy-delta 12,14-PGJ2 or dihomo-gamma-linolenic acid* or PGF3* or 15-keto-PGE* or 6-keto PGF1* or PGI3 or Thromboxane* or TXA2 or TXB2 or TXA1 or TXA3 or txb3).ab,ti,kw.

32. ((EPA ADJ2 Oxylipin*) or (eicosapentaenoic acid ADJ2 oxylipin*) or Resolvin* or resolvin D1 or resolvin D2 or D series resolvin* or RVD* or RvE* or 18S-RvE1 or hydroxyeicosatrienoic acid* or 5-hydroxyeicosatrienoic acid* or 12-hydroxyeicosatrienoic acid* or 15-hydroxyeicosatrienoic acid* or 5-HETrE or 15-HETrE or 12-HETrE or 5-oxo-ETrE or 15-deoxy- PGJ2 or 15-deoxy-Prostaglandin*).ab,ti,kw.

33. (Oxoeicosanoid* or Hydroperoxyeicosatetraenoic acid* or Hydroperoxy-eicosatetraenoic acid* or eicosatetraenoic acid* or hPETE* or 5-Hydroperoxyeicosatetraenoic acid* or 5-Hydroperoxy-eicosatetraenoic acid* or 5-HpETE or 5HPETE or 5 HPETE or 5-oxo-ETE or 8-hpete or 12-hpete or 15-hpete or 8hpete or 12hpete or 15hpete or 8 hpete or 12 hpete or 15 hpete or 12-oxo-ETE or 15-oxo-ETE or 5-hydroxy-eicosatetraenoic acid* or 12-hydroxy-eicosatetraenoic acid* or 8-hydroxy-eicosatetraenoic acid* or 15-hydroxy-eicosatetraenoic acid* or oxo-eicosatetraenoic acid* or oxo-ETE* or 5-oxo-eicosatetraenoic acid* or 15-oxo-eicosatetraenoic acid* or 12-oxo-eicosatetraenoic acid* or dihydroxy derivatives or 5,15-dihydroxy-eicosatetraenoic acid or 5,15-DiHETE or 8,15-dihydroxy-eicosatetraenoic acid or 8,15-DiHETE or 12,20-dihydroxy-eicosatetraenoic acid or 12,20-DiHETE or 5-LOX, 5-lypoxygenase activating protein or FLAP or Hepoxilin* or HxA3 or HxB3 or 12-HpETE or 8,9-dihydroxy-eicosatetraenoic acid or 8,9-DiHETE or 14,15-dihydroxy-eicosatetraenoic acid or 14,15-DiHETE or 17,18-dihydroxy-eicosatetraenoic acid or 7,18-DiHETE).ab,ti,kw.

34. (Lipoxin* or LXA* or LXB* or (hydroperoxy ADJ1 acid*) or Epi-lipoxin* or 15-Epi-lipoxin* or Epilipoxin* or 15- Epilipoxin* or 15-epi-LxA4 or AT-LxA4 or 15-epi-LxB4 or AT-LxB4 or Epoxyeicosatrienoic acid* or Epoxy-eicosatrienoic acid* or epoxyeicosatrienoate or epoxy-eicosatrienoate or EET* or 5,6-EET or 8,9-EET or 11,12-EET or 14,15-EET or 5,6-EEQ or 8,9-EEQ or 11,12-EEQ or 14,15-EEQ or 15,16-EEQ or Isoprostane* or isoPs or PG-like ADJ1 compound* or PG-like ADJ1 product* F_2_-isoprostane* or F_2_-IsoPs or F_4_-neuroprotane* or F_4_-NPs D2-isoPs or E2-isoPs or A2-isoPs or J2-isoPs or 5,6-epoxyisoprostane E2 or 5,6-epoxyisoprostane A2 or Isofuran* or dihydroxyeicosatrienoic or DHET* or soluble hydroxylase or 5-oxo-eicosatetraenoic acid or 5-oxo-ete).ab,ti,kw.

35. ((Linoleic acid ADJ2 Oxylipin*) or oxylipin* or OXLAM* or oxidized Linoleic Acid metabolites or  oxidized LA metabolites or ARA oxylipin* or (Arachadinoic Acid ADJ2 Oxylipin*) or AA oxylipin* or DHA oxylipin or (Docosahexaenoic acid ADJ2 oxylipin*) or COX oxylipin* or LOX oxylipin* or CYP oxylipin* or protectin* or NPD1 or neuroprotectin* or 17-epi-PD1 or maresin* or mar1 or mar2 or 7-epi Maresin* or 7-epi-mar1 or mar-l1 or mar-l2 or marl1 or marl2 or Oxylipin* or DHA oxylipin* or eicosapentaenoic acid- derived RvE1 or EPA-derived RvE1 or hydroxydocosahexaenoic acid* or HDoHE or protectin D1 or PD1 or linoleic acid-derived 9-hydroxyoctadecadienoic acid* or LNA-derived 9-hydroxyoctadecadienoic acid* or linoleic acid-derived 13-hydroxyoctadecadienoic acid* or LNA-derived 13-hydroxyoctadecadienoic acid* or hydroxyoctadecadienoic acid* or HODE or 9-HODE or 13-HODE or 9,10-dihydroxy-octadecenoic or 13-oxo-octadecadienoic acid* or 9-oxo-octadecadienoic acid* or 13-oxo-ODE or 9-oxo-ODE or 9-HpODE or 9-hydroperoxy-10E,12Z-octadecadienoic acid or 13-HpODE or 13-hydroperoxy-10E,12Z-octadecadienoic acid).ab,ti,kw.

36. (Hydroxy Fatty acid*or hydroxyl FA or 11-HETE or 11HETE or 11 HETE or 11-oxo-ETE or 13-HDoHE or 13HDoHE or 11-hydroxy-eicosatetraenoic acid* or 13-hydroxy-docosahexaenoic acid* or 9-hydroxy-octadecadienoic acid* or 17-hydroxy Docosahexaenoic Acid* or 17-HDoHE or 17-HDHA or hydroperoxyl-DHA or hydorperoxyl- docosahexaenoic acid or hp-DHA or hpDHA or 17-hydroperoxy-docosahexaenoic acid or 17-hydroperoxydocosahexaenoic acid or hydroxydha or 14-hpdha or 17s-hpdha or 14s-hpdha or hydroperoxy-docosahexaenoic acid or hydroxydocosahexaenoic acid or HDHA or 17-HDHA or 13-oxo-dha or 17-oxo-dha).ab,ti,kw.

37. (epoxy-eicosatrienoic acid* or EpETrE or epoxyeicosatetraenoic acid* or 5,6-epoxy-eicosatrienoic acid* or 5,6-EpETrE or 5,6-epoxyeicosatetraenoic acid* or 8,9- epoxy-eicosatrienoic acid* or 8,9-EpETrE or 8,9-epoxyeicosatetraenoic acid* or 11,12-epoxy-eicosatrienoic acid* or 11,12-EpETrE or 11,12-epoxyeicosatetraenoic acid* or 14,15-epoxy-eicosatrienoic acid* or 14,15-EpETrE or 14,15-epoxyeicosatetraenoic acid* or epoxy-eicosatetraenoic acid* or EpETE or EpDPE or EDP or HETE or hydroxyeicosapentaenoic acid* or hydroxy-eicosapentaenoic acid* or hydroxy eicosapentaenoic acid* or HEPE* or 5-hepe or 12-hepe or 15-hepe or 20-hepe or 5hepe or 12hepe or 15hepe or 20hepe or 8-HEPE or 8HEPE or 8 HEPE or 9-HEPE or 9HEPE or 9 HEPE or 18-HEPE or 18HEPE or 18 HEPE or HDoHE or dihydroxy-eicosatrienoic acid* or dihydroxyeicosatrienoic acid* or DiHETrE or 5,6-dihydroxy-eicosatrienoic acid* or 5,6-dihydroxyeicosatrienoic acid* or 8,9-dihydroxy-eicosatrienoic acid* or 8,9-dihydroxyeicosatrienoic acid* or 11,12-dihydroxy-eicosatrienoic acid* or 11,12-dihydroxyeicosatrienoic acid* or 14,15-dihydroxy-eicosatrienoic acid* or 14,15-dihydroxyeicosatrienoic acid* or 8,9-DiHETrE or 5,6-DiHETrE or 11,12-DiHETrE or 15,16-DiHETrE or DiHETE or dihydroxy-docosapentaenoic acid* or dihydroxydocosapentaenoic acid* or 5-oxo- eicosapentaenoic acid* or 5-oxo-EPE or oxo-eicosapentaenoic acid* or oxo-EPE or hydroperoxy-eicosapentaenoic acid* or HpEPE* or 12-hydroperoxy-eicosapentaenoic acid* or 15-hydroperoxy-eicosapentaenoic acid* or 12-HpEPE or 15-HpEPE).ab,ti,kw.

38. (dihydroxy-octadecatrienoic acid* or dihydroxyoctadecatrienoic acid* or dihydroxy octadecatrienoic acid* or diHOTrE or hydroxy-octadecatrienoic acid* or hydroxyoctadecatrienoic acid* or hydroxy octadecatrienoic acid* or HOTrE or Hydroperoxy-octadecatrienoic acid* or Hydroperoxyoctadecatrienoic acid* or hydroperoxy octadecatrienoic acid* or HpOTrE or oxo-octadecatrienoic acid* or oxooctadecatrienoic acid* or oxo octadecatrienoic acid* or oxo-OTrE or dihydroxy-octadecadienoic acid* or dihydroxyoctadecadienoic acid* or dihydroxy octadecadienoic acid*).ab,ti,kw.

39. (specialized pro-resolving mediator* or specialized proresolving mediator* or lipid mediator* or specialised pro-resolving mediator* or specialised proresolving mediator*).ab,ti,kw.

40. (Endocannabinoid* or ethanolamide* or n-acylethanolamide* or n acylethanolamide* or fatty acid ethanolamide* or cannabinoid* or endocannabinoid-like or Endocannabinoid system* or cannabinoid receptor* or CB1 or CB2 or Arachidonoylethanolamine* or 2-Arachidonoylglycerol or 2-Arachidonyl glyceryl ether* or O-arachidonoyl-ethanolamine* or N-Arachidonoyl dopamine* or ethanolamine* or aminoethanol* or n-acylethanolamine* or n acylethanolamine* or fatty acid ethanolamine* or Palmitoyl ethanolamide* or Palmitoylethanolamide* or Palmitoylethanolamine* or n-palmitoylethanolamine* or n palmitoylethanolamine* or palmidrol* or Hydroxyethylpalmitamide* or Heptadecanoyl ethanolamide* or Heptadecanoylethanolamide * or Heptadecanoylethanolamine* or n-heptadecanoylethanolamine* or n heptadecanoylethanolamine* or Heptadecanoyl ethanolamine* or Stearoyl ethanolamide* or Stearoylethanolamide* or Stearoylethanolamine* or n-stearoylethanolamine* or n Stearoyl ethanolamine* or Stearoyl ethanolamine* or Oleoyl ethanolamide* or Oleoylethanolamide* or Oleoylethanolamine* or n-oleoylethanolamine* or n Oleoylethanolamine* or Oleoylethanolamine* or Alpha-linolenoyl ethanolamide* or alpha-linolenoylethanolamine* or n-alpha-linolenoyl ethanolamine* or Alpha-linolenoyl ethanolamine* or n alpha-linolenoyl ethanolamine* or alpha-linolenoyl ethanolamine* or Linoleoyl ethanolamide* or Linoleoylethanolamide* or Linoleoylethanolamine* or n-Linoleoylethanolamine* or n Linoleoylethanolamine* or Linoleoyl ethanolamine* or Anandamide* or n-arachidonoylethanolamine* or arachidonoyl ethanolamide* or arachidonoylethanolamide* or Eicosadienoyl ethanolamide* or Eicosadienoylethanolamine* or n arachidonoylethanolamine* or Arachidonoyl ethanolamine* or arachidonoyl glycerol* or Docosatetraenoyl ethanolamide* or Docosatetraenoylethanolamide* or Docosatetraenoylethanolamine* or n-docosatetraenoylethanolamine* or n docosatetraenoylethanolamine* or Docosatetraenoyl ethanolamine* or Eicosapentanoyl ethanolamide* or Eicosapentanoylethanolamide* or Eicosapentanoylethanolamine* or n-Eicosapentanoylethanolamine* or n Eicosapentanoylethanolamine* or Eicosapentanoyl ethanolamine* or Docosapentaenoyl ethanolamide* or Docosapentaenoylethanolamide* or Docosapentaenoylethanolamine* or n-docosapentaenoylethanolamine* or n docosapentaenoylethanolamine* or docosapentaenoyl ethanolamine* or Docosahexanoylethanolamide* or Docosahexanoyl ethanolamide* or Docosahexanoylethanolamine* or n-docosahexanoylethanolamine* or n docosahexanoylethanolamine* or docosahexanoyl ethanolamine* or synaptamide or synaptamine* or dihomo-gama-linolenoyl ethanolamide* or linolenoyl ethanolamide* or inolenoyl ethanolamimine* or dihomo-gama-linolenoyl ethanolamine* or dihomo gamma linolenoyl ethanolamide* or fatty acid amide hydrolase* or faah*).ab,ti,kw.

41. (Phospholipase A2 or PLA2 or sPLA2 or cPLA2 or ipla2 or lp-PLA2 or pla2g4a or pla2g6 or Phosphoinositide phospholipase C or Phospholipase C or PLC or Phospholipase* or Lecithinase* or Lypoxygenase*or LOX*or Arachidonate 5-lipoxygenase* or ALOX5 or 5-lipoxygenase* or 5-LOX or 5-LO or 5LOX or 5LO or 5 LOX or Arachidonate 12-lipoxygenase* or ALOX12 or 12-lipoxygenase or 12-LOX or 12-LO or 12LOX or 12LO or 12 LOX or 15-LOX or 15LOX or 15 LOX or 15-LO or 15LO or Arachidonate 15-lipoxygenase-1 or ALOX15 or 15-lipoxygenase-1 or erythrocyte type 15-lipoxygenase* or 15-lipoxygenase* or reticulocyte type 15-lipoxygenase* or 15-lipoxygenase, reticulocyte type or 15-LO-1 or 15-LOX-1 or 15LO1 or 15LOX1 or Arachidonate 15-lipoxygenase* or Arachidonate 15-lipoxygenase type II or ALOX15B or 15-lipoxygenase-2 15-LOX-2 or 15-LOX-2 or Arachidonate 12-lipoxygenase* or 12R type ALOX12B or 12R-lipoxygenase or 12R-LOX or 12R-LO or platelet-type lipoxygenase 12 or ALOX12 or ALOX15 or ALOX5 or leukocyte-type arachidonate 15-lipoxygenase or Cytochrome p450 or CYP* or cyclooxygenase* or COX2 or COX-2 or COX1 or COX-1 or COX* or prostaglandin-endoperoxide synthase* or Prostaglandin E Synthase 2 or Prostaglandin-E Synthase or PTGS1 or PTGES2 or PGES* or mPGES or cPGES or PTGES* or (Prostaglandin ADJ3 Synthase*) or (5-lypoxygenase activating ADJ1 protein) or FLAP or epoxygenase* or CYP epoxygenase* or Cytochrome P450 omega hydroxylase* or cytochrome P450 omega-hydroxylase* or CYP450 omega hydroxylase* or CYP450 omega-hydroxylase* or CYP omega hydroxylase* or CYP omega-hydroxylase* or fatty acid omega hydroxylase* or cytochrome P450 monooxygenase* or fatty acid monooxygenase* or docosahexaenoic acid omega-hydroxylase* or docosahexaenoate,NADPH:O2 oxidoreductase* or P450 omega-hydroxylase* or thromboxane A synthase* or thromboxane-A synthase* or TBXAS1 or acyltransferase lysolecithin-lysolecithin or l2, lysophospholipase or lecithinase b or lysolecithin lysolecithin acyltransferase* or lysolecithin-lysolecithin acyltransferase or lysophospholipase* or lysophospholipase a or lysophospholipase a1 or lysophospholipase c or lysophospholipase l2 or phospholipase b or phospholipase c or phospholipase*, type c or type c phospholipase* or arachidonate lipoxygenase* or arachidonic acid lipoxygenase* or lipoxygenase* arachidonic acid or lipoxygenase* or Elovanoid*).ti,ab,kw.

42. 26 or 27 or 28 or 29 or 30 or 31 or 32 or 33 or 34 or 35 or 36 or 37 or 38 or 39 or 40 or 41

43. 16 and 25 and 42

**APPENDIX 3 – SEARCH WITH PSYCINFO APPENDIX**

1. brain*.ab,ti.

2. cereb*.ab,ti.

3. central nervous system.ab,ti.

4. CNS.ab,ti.

5. ((cerebr* adj3 tissue*) or (brain adj3 tissue*) or (cerebral adj3 sample*) or (brain adj3 sample*)).ab,ti.

6. exp central nervous system/ or exp brain/ or exp blood brain barrier/ or exp gray matter or exp white matter/ or exp forebrain/ or exp brain stem/ or exp fornix/ or exp cerebral cortex/

7. exp mesencephalon/ or exp cerebral peduncle/ or exp cerebral crus/ or exp substantia nigra/ or exp pars compacta/ or exp pars reticulata/ or exp tegmentum mesencephali/ or exp cerebral aqueduct/ or exp midbrain reticular formation/ or exp pedunculopontine tegmental nucleus/ or exp oculomotor nuclear complex/ or exp edinger-westphal nucleus/ or exp periaqueductal gray/ or exp raphe nuclei/ or exp dorsal raphe nucleus/ or exp interpeduncular nucleus/ or exp midbrain raphe nuclei/ or exp red nucleus/ or exp ventral tegmental area/ or exp locus coeruleus/ or exp tectum mesencephali/ or exp inferior colliculi/ or exp posterior cerebellar commissure/ or exp pretectal region/ or exp subcommissural organ/ or exp superior colliculi/ or exp reticular formation/ or exp respiratory center/ or exp rhombencephalon/ or exp metencephalon/ or exp cerebellum/ or exp cerebellar cortex/ or exp cerebellar vermis/ or exp purkinje cells/ or exp cerebellar nuclei/ or exp cerebellopontine angle/ or exp pons/ or exp barrington's nucleus/ or exp cochlear nucleus/ or exp kolliker-fuse nucleus/ or exp middle cerebellar peduncle/ or exp pontine tegmentum/ or exp abducens nucleus/ or exp facial nucleus/ or exp parabrachial nucleus/ or exp nucleus raphe magnus/ or exp superior olivary complex/ or exp trapezoid body/ or exp trigeminal motor nucleus/ or exp trigeminal nucleus, spinal/ or exp vestibular nuclei/ or exp vestibular nucleus, lateral/ or exp myelencephalon/ or exp medulla oblongata/ or exp area postrema/ or exp olivary nucleus/ or exp nucleus raphe obscurus/ or exp nucleus raphe pallidus/ or exp solitary nucleus/ or exp trigeminal caudal nucleus/ or exp tectospinal fibers/ or exp trigeminal nuclei/ or exp cerebral ventricles/ or exp choroid plexus/ or exp ependyma/ or exp fourth ventricle/ or exp lateral ventricles/ or exp septum pellucidum/ or exp third ventricle/ or exp prosencephalon/ or exp diencephalon/ or exp epithalamus/ or exp habenula/ or exp pineal gland/ or exp hypothalamus/ or exp hypothalamic area, lateral/ or exp hypothalamus, anterior/ or exp anterior hypothalamic nucleus/ or exp organum vasculosum/ or exp paraventricular hypothalamic nucleus/ or exp preoptic area/ or exp suprachiasmatic nucleus/ or exp suprachiasmatic nucleus neurons/ or exp supraoptic nucleus/ or exp hypothalamus, middle/ or exp "arcuate nucleus of hypothalamus"/ or exp dorsomedial hypothalamic nucleus/ or exp hypothalamo-hypophyseal system/ or exp median eminence/ or exp pituitary gland/ or exp pituitary gland, anterior/ or exp corticotrophs/ or exp gonadotrophs/ or exp lactotrophs/ or exp somatotrophs/ or exp thyrotrophs/ or exp pituitary gland, intermediate/ or exp melanotrophs/ or exp pituitary gland, posterior/ or exp tuber cinereum/ or exp ventromedial hypothalamic nucleus/ or exp hypothalamus, posterior/ or exp mammillary bodies/ or exp optic chiasm/ or exp optic tract/ or exp subthalamus/ or exp entopeduncular nucleus/ or exp subthalamic nucleus/ or exp zona incerta/ or exp thalamus/ or exp thalamic nuclei/ or exp anterior thalamic nuclei/ or exp geniculate bodies/ or exp intralaminar thalamic nuclei/ or exp lateral thalamic nuclei/ or exp pulvinar/ or exp mediodorsal thalamic nucleus/ or exp midline thalamic nuclei/ or exp posterior thalamic nuclei/ or exp ventral thalamic nuclei/ or exp telencephalon/ or exp cerebrum/ or exp "diagonal band of broca"/ or exp external capsule/ or exp internal capsule/ or exp olfactory bulb/ or exp "septum of brain"/ or exp septal nuclei/ or exp telencephalic commissures/ or exp anterior cerebellar commissure/ or exp corpus callosum/ or exp meninges/ or exp ganglia/ or exp ganglia, autonomic/ or exp ganglia, parasympathetic/ or exp ganglia, sympathetic/ or exp stellate ganglion/ or exp superior cervical ganglion/ or exp ganglia, sensory/ or exp ganglia, spinal/ or exp geniculate ganglion/ or exp nodose ganglion/ or exp spiral ganglion/ or exp trigeminal ganglion/

8. (Brain* or hippocamp* or encephalon or Blood Brain Barrier or hemato-encephalic barriers or barriers brain-blood or hemato encephalic barrier or barriers hemato-encephalic or barrier hemato-encephalic or hemato-encephalic barrier or truncus cerebrus or truncus cerebri or cerebri truncus or brainstems or cerebrus truncus or Mesencephalon or mesencephalon or mesencephalons or midbrains or midbrain or Cerebral Peduncle or Cerebral Crus or Substantia Nigra or nigras substantia or nigra substantia or substantia nigras or Pars Compacta or Pars Reticulata or Tegmentum Mesencephali or midbrain trigeminal nucleus or nucleus peripeduncular or annulari nucleus or nervi trochlearis nucleus or midbrain tegmentum or mesencephalus tegmentum or tegmental nucleus ventral or mesencephalic tegmentums or midbrain tegmentums or trigeminal nucleus mesencephalic or tegmentums midbrain or trochlearis nucleus nervi or nucleus annularis or trigeminal nucleus midbrain or nucleus annular or mesencephali tegmentum or darkshevichs nucleus or tegmentums mesencephalic or ventral tegmental nucleus or mesencephalic trigeminal nucleus or nervi trochleari nucleus or nucleus darkshevich's or darkschewitsch nucleus or tegmentum of midbrain or nucleus annulari or cajal interstitial nucleus or mesencephalic tegmentum or nuclei accessory oculomotor or trochlear nucleus or annularis nucleus or nucleus mesencephalic trigeminal or nucleus of darkschewitschor peripeduncular nucleus or oculomotor nuclei accessory or tegmentum midbrain or tegmentum mesencephali or nucleus nervi trochlearis or darkshevich nucleus or nucleus tractus mesencephalici nervi trigemini or interstitial nucleus of cajal or Cerebral Aqueduct or ducts mesencephalic or mesencephalic ducts or aqueduct mesencephalic or sylvian aqueducts or duct mesencephalic or sylvius aqueduct or cerebrus aqueductus or aqueductus cerebrus or cerebral aqueduct or aqueduct sylvian or aqueduct of sylvius or mesencephalic duct or cerebral aqueducts or aqueducts sylvian or aqueduct cerebral or sylvian aqueduct or aqueductus cerebri or aqueducts mesencephalic or cerebri aqueductus or mesencephalic aqueduct or Midbrain Reticular Formation or Pedunculopontine Tegmental Nucleus or nucleus tegmentalis pedunculopontinus or nucleus pedunculopontine tegmental or tegmental nucleus pedunculopontine or pedunculopontine tegmental nucleus or Oculomotor Nuclear Complex or Edinger-Westphal Nucleus or Periaqueductal Grey or greys central periaqueductal or griseum centrales or central grey substance of midbrain or periaqueductal greys central or grey matter periaqueductal or grey central periaqueductal or substantia grisea centralis or periaqueductal grey matter or central periaqueductal grey or grisea centralis substantia or periaqueductal grey or centrale mesencephali griseumor centrale mesencephalus griseum or centrale griseum or grey matters periaqueductalor centrales griseum or periaqueductal grey central or substantia grisea centralis mesencephali or mesencephalus griseum central or midbrain central grey or central grey mesencephalic or central periaqueductal greys or central grey midbrain or griseum centrale mesencephali or Raphe Nuclei or nucleus incertus or nucleus superior central ornuclei raphe ornucleus interfascicular or superior central nucleus or raphe nuclei or interfascicular nucleus or raphe nucleus or incertus nucleus or central nucleus superior or rostral linear nucleus of the raphe or caudal linear nucleus of the raphe or rostral linear nucleus of raphe or nucleus rapheor Dorsal Raphe Nucleus or Interpeduncular Nucleus or Midbrain Raphe Nuclei or Red Nucleus or nucleus ruber or red nucleus or nucleus red or Ventral Tegmental Area or tegmentalis ventralis area or tegmentalis ventrali area or area tegmentalis ventralis or ventral tegmental area of tsai or ventral tegmental area or tegmental area ventral or area tegmentalis ventrali).ti,ab.

9. (Locus Coeruleus or coeruleus complex locus or complices locus coeruleus or locus caeruleus or complex locus ceruleus or complices locus ceruleus or coeruleus complices locus or ceruleus complex locus or locus ceruleus complex or complex locus coeruleus or locus ceruleus complices or locus ceruleus or nucleus pigmentosus pontis or locus coeruleus complices or pontis nucleus pigmentosus or ceruleus complices locus or locus coeruleus or locus coeruleus complex or Tectum Mesencephali or corpora quadrigemina or inferior colliculus commissures or colliculus commissures superior or colliculus commissures inferior or quadrigeminal plates or superior colliculus commissure or plate quadrigeminal or commissure of superior colliculus or quadrigemina corpora or commissure of inferior colliculus or lamina quadrigemina or inferior colliculus commissure or colliculus commissure inferior or quadrigeminal plate or tectum mesencephalus or mesencephalus tectum or plates quadrigeminal or quadrigemina lamina or colliculus commissure superior or Inferior Colliculi or colliculi inferior or inferior colliculi or inferiors colliculus or posterior colliculus or brachial nucleus of the inferior colliculus or caudal colliculus or colliculus inferiors or colliculus caudal or inferior colliculus or colliculus posterior or colliculus inferior or Subcommissural Organ or subcommissural organs or subcommissural organ or organs subcommissural or organ subcommissural or Superior Colliculi or mammalian optic lobesor optic lobe mammalian or optic tectums or superior colliculi or optic tectum or anterior colliculus or colliculus superior or human optic lobes or superior colliculus or optic lobes human or optic lobes mammalian or optic lobe human or colliculi superior or tectum optic or tectums optic or mammalian optic lobe or human optic lobe or colliculus anterior or Reticular Formation or formations reticular or reticular formation or reticular formations or formation reticular or edunculopontine Tegmental Nucleus or nucleus tegmentalis pedunculopontinus or nucleus pedunculopontine tegmental or tegmental nucleus pedunculopontine or pedunculopontine tegmental nucleus or Respiratory Center or centers respiratory or respiratory centers or center respiratory or respiratory center or hombencephalon or hind brains or brains hind or rhombencephalons or hindbrain or hindbrains or brain hind or rhombencephalon or hind brain or Medulla Oblongata or medulla oblongata or nucleus ambiguous or arcuate nucleus-1 or accessory cuneate nucleus or nucleus external cuneate or cuneate nucleus accessory or nucleus ambiguous or medulla oblongatas or arcuate nucleus of the medulla or cuneate nucleus lateralor nucleus lateral cuneate or ambiguous nucleus or cuneate nucleus external or arcuate nucleus 1 or external cuneate nucleus or ambiguus nucleus or arcuate nucleus-1s or lateral cuneate nucleus or Area Postrema or area postremas or trigger zone chemoreceptor or chemoreceptor trigger zone or chemoreceptor trigger zones or trigger zones chemoreceptor or zone chemoreceptor trigger or postrema area or zones chemoreceptor trigger or area postrema or Olivary Nucleus ornucleus basalis olivary or nucleus olivary or basalis olivary nucleus or nucleus olivary basal or olivary basal nucleus or basal nucleus olivary or olivary nucleus or Raphe nuclei or nucleus incertus or nucleus superior central or nuclei raphe or nucleus interfascicular or superior central nucleus or raphe nuclei or interfascicular nucleus or raphe nucleus or incertus nucleus or central nucleus superior or rostral linear nucleus of the raphe or caudal linear nucleus of the raphe or rostral linear nucleus of raphe or nucleus raphe or Nucleus Raphe Obscurus or Nucleus Raphe Pallidus or Solitary Nucleusor solitary nuclear complices or nucleus of tractus solitaries or complex solitary nuclear or tractus solitarii nuclei or nucleus solitaries or solitarius nucleus tractus or tractus solitarius nucleus or solitarius nuclei tractus or solitary tract nucleus or nucleus solitary tract or solitary nuclear complex or tractus solitarius nuclei or nuclear complices solitary or nuclei tractus solitarii or solitary nucleus ornucleus solitaryor nucleus of the solitary tract or nuclear complex solitary or complices solitary nuclear or nucleus of solitary tract or nucleus tractus solitaries or Trigeminal Nucleus, Spinal or trigeminal nucleus spinal or nucleus spinal trigeminal or spinal trigeminal nucleus or Trigeminal Caudal Nucleus or caudal nucleus trigeminal or nucleus trigeminal caudal or trigeminal caudal nucleus or Metencephalon or Cerebellumor corpus cerebellus or parencephalons or cerebellus corpus or cerebellum or cerebellums or corpus cerebelli or parencephalon or cerebelli corpus or Cerebellar Cortex or cerebelli cortex or cortex cerebellus or cerebellar cortex or cortex cerebelli orcerebellus cortex or cortex cerebellar or Cerebellar Vermis or Purkinje Cells or purkinje cells or cells purkinje or Cerebellar Nuclei or nucleus dentatus or Cerebellopontine Angle or central nucleus or central nucleus or interposed nucleus anterior or nucleus globosus or medial cerebellar nucleus or emboliformis nucleus or nuclei cerebellar or intracerebellar nuclei or nucleus fastigii or nucleus fastigial or fastigii nucleus or central nuclei or nuclei central or deep cerebellar nucleus or intracerebellar nucleus or nucleus fastigial cerebellar or nucleus anterior interposed or nucleus intracerebellar oranterior interposed nucleus or nucleus anterior interpositus or nucleus medial cerebellar or nuclei intracerebellar or nucleus dentate or dentate nucleus or interpositus nucleus anterior or globosus nucleus orcerebellar nucleus deep or nucleus central or nucleus cerebellar or cerebellar nuclei deep or nucleus dentate cerebellar or anterior interpositus nucleus or cerebellar nucleus medial or cerebellar nuclei or fastigial cerebellar nucleus or Pons or pons or varolii ponsor pontes or pons varolius or varolius pons or pons varolii or ponte or Barrington's Nucleusor Cochlear Nucleus or cochlear nucleus or nuclei cochlear or cochlear nuclei or nucleus cochlear or Kolliker-Fuse Nucleus or Middle Cerebellar Peduncle or Pontine Tegmentum or Abducens Nucleus or Facial Nucleus or Parabrachial Nucleus or Nucleus Raphe Magnus or Superior Olivary Complex or Trapezoid Body or Trigeminal Motor Nucleus or Vestibular Nuclei or schwalbes nucleus or nucleus schwalbe or vestibular nuclei or vestibular nucleus medial or nuclei vestibular or schwalbe's nucleus or nucleus schwalbe's or medial vestibular nucleus or schwalbe nucleus or nucleus medial vestibular or Vestibular Nucleus, Lateral or deiters nucleus or deiter's nucleus or nucleus of deiters or lateral vestibular nucleus or nucleus lateral vestibular or vestibularis laterali nucleus or nucleus vestibularis laterali or vestibular nucleus lateral or vestibularis magnocellulari nucleus or vestibularis magnocellularis nucleus or deiter nucleus or nucleus vestibularis magnocellularis or nucleus vestibularis magnocellulari or nucleus deiter or nucleus vestibularis lateralis or vestibularis lateralis nucleus or nucleus deiter's or Tectospinal Fibers or Trigeminal Nuclei or trigeminal nucleus or trigeminal nuclear complices or trigeminal nuclear complex or nuclei trigeminal or trigeminal nuclei or nucleus trigeminal or nuclear complices trigeminal or nuclear complex trigeminal or Grey Matter or grey matter or grey matters cerebellar or grey matter cerebellar or matters grey or matter cerebellar grey or grey matter cerebellar or cerebellar grey matters or grey matters or matter cerebellar grey or cerebellar grey matter or matters grey or cerebellar grey matters or matters cerebellar grey or grey matters cerebellar or matters cerebellar grey or grey matter or cerebellar grey matter or matter grey or matter grey or White Matter or white matter cerebellar or matter cerebellar white or matter white or matters cerebellar white or white matters cerebellar or cerebellar white matters or cerebellar white matter or matters white or white matter or white matters or Cerebral Ventricles or cerebral ventricle or cerebral ventriclesor monro foramen or ventricles cerebral or foramen of monro or cerebral ventricular system or ventricle cerebral or Choroid Plexus or choroideus plexus or plexus choroideusor choroid plexus or chorioid plexus or plexus chorioid or plexus choroid or Ependyma or ependymal or ependymas or Fourth Ventricle or ventricolo quarto or ventricles fourth or ventricle fourth or 4th ventricle or quarto ventricolos or ventricle 4th or ventricles 4th or fourth ventricle or ventricolos quarto or fourth ventricles or 4th ventricles or quarto ventricolo or Lateral Ventricles or lateral ventricle orsubventricular zones or lateral ventricles or ventricle lateral or zone subventricular or ventricles lateral or subventricular zone or zones subventricular or Septum Pellucidum or septum supracommissural or pelusidum septum or septum pellucidum or lucidums septum or supracommissural septum or pellucidum septum or septum pelusidums or septum pelusidum or pelusidums septum or septum lucidums or supracommissural septums or septums supracommissural or lucidum septum or septum lucidum or Third Ventricle or 3rd ventricle or ventricles third or ventricles 3rd or third ventricle or ventricle 3rd or 3rd ventricles or third ventricles or ventricle third or Limbic System or limbic system or system limbic or systems limbic or limbic systems or Amygdala or amygdaloid bodies or corpus amygdaloideums or nucleus intercalated amygdaloid or corpus amygdaloideum or amygdaloid body or complex amygdaloid nuclear or amygdaloid nuclear complices or amygdaloid nucleusor intercalata massa or amygdaloideums corpus or intercalatas massa or amygdaloid nucleus intercalated or nuclear complices amygdaloid or archistriatums or amygdala or massa intercalates or nucleus amygdaloid or amygdaloideum corpus or amygdalae nucleus or nuclear complex amygdaloid or archistriatum or nucleus amygdalae or amygdaloid nuclear complex or Basolateral Nuclear Complex or Central Amygdaloid Nucleus or Corticomedial Nuclear Complex or Periamygdaloid Cortex or epithalamus or Habenula or commissure habenular or habenula complex or habenulas or complices habenula or nucleus habenularis or habenular commissures or complex habenula or habenula complices or nucleus habenular or nucleus habenulari or commissures habenular or habenula or habenularums commissura or commissura habenularum or habenularis nucleus or habenular nuclei or commissura habenularums or nuclei habenular or habenulari nucleus or habenular nucleus or Pineal Gland or pineales corpus or body pineal or glands pineal or pineal glands or pineal body or cerebri epiphysis or corpus pineales or gland pineal or pineale corpus or bodies pineal or corpus pineale or pineal gland or pineal bodies or epiphysis cerebri).ti,ab.

10. (Hippocampus or hippocampal formation or propers hippocampus or hippocampus propers or formations hippocampal or horn ammon's or schaffer collateral or ammon horn or hippocampus or horn ammon or cornu ammonis or hippocampus proper or proper hippocampus or collaterals schaffer or formation hippocampal or hippocampal formations or subiculum or subiculums or ammon's horn or CA1 Region, Hippocampal or regio superior of hippocampus or field hippocampus ca1 or ca1 stratum radiatum or stratum radiatum ca1 or hippocampal sector ca1 or hippocampus ca1 field or hippocampus regio superior or ca1 stratum radiatums or sector ca1 hippocampal or ca1 field hippocampus or radiatums ca1 stratum or stratum radiatums ca1 or ca1 hippocampal sector or ca1 pyramidal cell area or ca1 region hippocampal or ca1 pyramidal cell layer or ca1 stratum pyramidale or stratum pyramidale ca1 or cornu ammonis 1 area or radiatum ca1 stratum or CA2 Region, Hippocampal or ca2 stratum pyramidale or radiatums ca2 stratum or cornu ammonis 2 area or ca2 field hippocampus or stratum pyramidale ca2 or stratum radiatum ca2 or ca2 stratum radiatums or radiatum ca2 stratum or sector ca2 hippocampal or region hippocampal ca2 or ca2 field of hippocampus or stratum radiatums ca2 or ca2 region hippocampal or hippocampal sector ca2 or hippocampal ca2 region or hippocampus ca2 field or ca2 pyramidal cell layer or field hippocampus ca2 or ca2 pyramidal cell area or CA3 Region, Hippocampal or stratum lucidum ca3 or ca3 stratum lucidum or stratum lucidums ca3 or lucidum ca3 stratum or ca3 region hippocampal or ca3 pyramidal cell area or hippocampus ca3 field or ca3 hippocampal sector or sector ca3 hippocampal or ca3 stratum radiatum or ca3 stratum lucidums or hippocampal ca3 regions or cornu ammonis 3 area or ca3 field of hippocampus or radiatum ca3 stratum or field hippocampus ca3 or stratum radiatums ca3 or ca3 pyramidal cell layer or lucidums ca3 stratum or region hippocampal ca3 or radiatums ca3 stratum or ca3 stratum pyramidale or ca3 field hippocampus or Dentate Gyrus or ca4 region hippocampal or dentate fascia or cornu ammonis 4 area or hilus gyri dentate or ca4 field of hippocampal formation or ca4 hippocampal sector or gyrus dentate or sector ca4 hippocampal or hippocampal ca4 region or area dentata or region hippocampal ca4 or dentata area or field hippocampal ca4 or gyrus dentatus or hilus of the fascia dentata or hilus of dentate gyrus or dentate gyrus or area dentatas or dentata fascia or hippocampal sector ca4 or hippocampal ca4 field or ca4 of lorente de no or Mossy Fibers, Hippocampal or hippocampal mossy fiber or mossy fibers hippocampal or hippocampal mossy fibers or mossy fiber hippocampal or Fornix, Brain or hippocampal commissure or hippocampal commissures or commissures dorsal hippocampal or fornix commissures or fornices or brain fimbrias or fornical commissures or fornical commissure or fornix or hippocampal commissures dorsal or commissures hippocampal or fornix-fimbria or hippocampal commissure dorsal or fimbria or fornix fimbria or fimbria of hippocampus or brain fornices or dorsal hippocampal commissure or commissure fornical or commissure dorsal hippocampal or commissure of fornix or commissures fornical or commissure hippocampal or fornix commissure or fimbria-fornix or fimbria fornix or fimbria brain or hippocampus fimbrias or hippocampus fimbria or brain fimbria or Hypothalamus or preoptico-hypothalamic areas or preoptico hypothalamic area or lamina terminalis or hypothalamus or areas preoptico-hypothalamic or area preoptico-hypothalamic or preoptico-hypothalamic area or Hypothalamic Area, Lateral or area hypothalamica laterali or hypothalamica laterali area or hypothalami area lateralis or lateralis area hypothalamica or hypothalamus area lateralis or laterali area hypothalamica or areas lateral hypothalamic or lateralis hypothalami area or lateral hypothalamic areas or accessory nucleus of the ventral horn or lateral tuberal nuclei or tuberal nucleus lateral or lateral hypothalamus or area hypothalamica lateralis or hypothalamus lateral or tuberomammillary nucleus or hypothalamic area lateral or nucleus tuberomammillary or nuclei lateral tuberal or nucleus lateral hypothalamic or lateralis hypothalamus area or area lateral hypothalamic or hypothalamic nucleus lateral or area lateralis hypothalamus or nucleus lateral tuberal or Hypothalamus, Anterior or commissures anterior hypothalamic or anterior hypothalamic decussation of ganser or hypothalamic commissures anterior or anterior hypothalamic commissures or commissure anterior hypothalamic or periventricular nucleus anteroventral or nucleus anteroventral periventricular or anterior hypothalamic commissure or hypothalamic commissure anterior or hypothalamus anterior or hypothalamus supraoptic or anteroventral periventricular nucleus or anterior hypothalamus or supraoptic hypothalamus or Anterior Hypothalamic Nucleus or areas anterior hypothalamic or hypothalamic area anterior or nucleus anterior hypothalamic or anterior hypothalamic nucleus or hypothalami nucleus anterior or hypothalamic areas anterior or anterior hypothalami nucleus or anterior hypothalamic area or area anterior hypothalamic or nucleus anterior hypothalamus or hypothalamus nucleus anterior or anterior hypothalamic areas or anterior hypothalamus nucleus or nucleus anterior hypothalami or hypothalamic nucleus anterior).ab,ti.

11. (Organum Vasculosum or Paraventricular Hypothalamic Nucleus or hypothalamic paraventricular nucleus or paraventricular hypothalamic nucleus or nucleus paraventricular hypothalamic or nucleus hypothalamic paraventricular or nucleus paraventricular or paraventricular nucleus or hypothalamic nucleus paraventricular or paraventricular nucleus hypothalamic or Preoptic Area or area medial preoptic or preoptic area medial or preoptic nucleus or nuclei preoptic or lateral preoptic area or preoptic areas lateral or area preoptic or areas medial preoptic or area lateral preoptic or preoptic areas medial or lateral preoptic areas or preoptica area or nucleus preoptic or medial preoptic areas or areas lateral preoptic or area preoptica or areas preoptic or preoptic nuclei or medial preoptic area or preoptic area or preoptic areas or Suprachiasmatic Nucleus or nucleus suprachiasmatic or suprachiasmatic nucleus or Supraoptic Nucleus or hypothalamus supraoptic nucleus or supraoptic group accessory or accessory supraoptic groups or supraoptic nucleus of hypothalamus or supraopticus nucleus or groups accessory supraoptic or nucleus supraoptic or group accessory supraoptic or accessory supraoptic group or nucleus supraopticus or supraoptic groups accessory or supraoptic nucleus or Hypothalamus, Middle or regions intermediate hypothalamic or hypothalamic region intermediate or region intermediate hypothalamic or middle hypothalamus or hypothalamus medial or hypothalamic regions intermediate or intermediate hypothalamic regions or intermediate hypothalamic region or hypothalamus middle or medial hypothalamus or Arcuate Nucleus of Hypothalamus or nucleus arcuate or arcuate nucleus or hypothalamus arcuate nucleus or nucleus infundibular or infundibular nucleus or arcuate nucleus of hypothalamus or Dorsomedial Hypothalamic Nucleus or nucleus arcuate or arcuate nucleus or hypothalamus arcuate nucleus or nucleus infundibular or infundibular nucleus or arcuate nucleus of hypothalamus or Hypothalamo-Hypophyseal System or hypothalamic pituitary unit or hypothalamo hypophyseal system or hypothalamo-hypophyseal system or hypothalamic-pituitary unit or Median Eminence or eminentia medianas or median eminence or eminences medial or eminence medial or medial eminences or medianas eminentia or eminentia mediana or mediana eminentia or eminence median or medial eminence or Pituitary Gland or hypophyseal infundibulum or infundibular hypothalamus or pituitary glands or infundibulum or stalk infundibular or hypothalamus infundibular or infundibulums or pituitary stalks or pituitary gland or hypophysis or pituitary stalk or infundibular stem or stalks infundibular or glands pituitary or hypophysis cerebri or hypophyseal stalks or cerebri hypophysis orstalk hypophyseal or infundibular stalk or infundibular stalks or hypophysis cerebrus or hypophyseal stalk or Pituitary Gland, Anterior or lobus anteriors or anterior lobe of pituitary or anterior lobus or pituitary pars distalis or anterior pituitary glands or anteriors lobus or lobus anterior or pituitary gland anterior or adenohypophyses or pituitary glands anterior or adenohypophysis or pituitary anterior lobe or anterior pituitary gland or pars distalis of pituitary or Corticotrophs or Gonadotrophs or lh producing cells or lh-secreting cells or fsh cells or gonadotrophs or lh cell or fsh-secreting cellsor fsh secreting cells or fsh-producing cells or fsh-producing cell or fsh cell or lh-producing cells or fsh producing cells or lh secreting cells or fsh-secreting cell or gonadotroph or lh-producing cell or lh-secreting cell or lh cell or Lactotrophs or pituitary prolactin-secreting cells or lactotrophs or pituitary prolactin cell or prolactin-secreting cell pituitary or prolactin-secreting cells pituitary or lactotroph or prolactin cell pituitary or prolactin cells pituitary or pituitary prolactin cells or pituitary prolactin-secreting cell or pituitary prolactin secreting cells or Somatotrophs or gh cell pituitary or somatotrophs or gh cells pituitary or pituitary growth hormone-secreting cells or pituitary gh cell or pituitary growth hormone secreting cells or pituitary gh cells or somatotroph or Thyrotrophs or Pituitary Gland, Intermediate or Melanotrophs or Pituitary Gland, Posterior or lobes neural or posterior pituitary glands or neural lobe or pituitary pars nervosa or infundibular processes or infundibular process or process infundibular or neurohypophysis or lobe neural or gland posterior pituitary or pituitary posterior lobe or pars nervosa of pituitary or posterior lobe of pituitary or neural lobes or nervosus lobus or lobus nervosus or pituitary gland posterior or processes infundibular or Tuber Cinereum or cinereums tuber or cinereum tuber or tuber cinereum or tuber cinereums or Ventromedial Hypothalamic Nucleus or nucleus ventromedial hypothalamic or hypothalamic nucleus ventromedial or ventromedial hypothalamic nucleus or Hypothalamus, Posterior or posteriors area hypothalamica or area hypothalamica posterior or mammillary regions or region mammillary or nucleus posterior periventricular or hypothalamic regions posterior or hypothalamus posteriors or mammillary region or posterior area hypothalamica or posterior hypothalamic regions or supramammillary commissures or region posterior hypothalamic or supramammillary commissure or regions posterior hypothalamic or posterior hypothalamus or commissures supramammillary or premammillary nucleus or hypothalamic region posterior or posterior hypothalamic region or commissure supramammillary or hypothalamus posterior or hypothalamica posteriors area or periventricular nucleus posterior or nucleus premammillary or Mammillary Bodies or mammillary bodies ormammillary body or body mammillary or mamillary bodies or body mamillary or bodies mamillary or bodies mammillary or mamillary body or Limbic Lobe or Gyrus Cinguli or gyrus cingular or anterior cingulate gyrus or cingulate gyri posterior or cortex anterior cingulate or posterior cingulate gyrus or cinguli anteriors gyrus or mesial region superior or gyrus cingulate or cingulate cortex anterior or cingulate cortex or superior mesial regions or regions cingulate or cortex posterior cingulate or anterior cingulate cortices or posterior cingulates or cingulate bodies or cingulates anterior or cortices anterior cingulate or posterior cingulate cortices or mesial regions superior or posterior cingulate cortex or regions posterior cingulate or cingulate posterior or posterior cingulate region or region posterior cingulate or body cingulate or cortex cingulate or posterior cingulate regions or cingulate gyrus anterior or cingulate gyrus or cingulate gyrus posterior or cingular gyrus or bodies cingulate or cingulate area or anterior cingulates or area cingulate or cingulate regions or regions superior mesial or ingulates posterior or areas cingulate or cingulate cortices anterior or anterior gyrus cinguli or gyri posterior cingulate or gyrus anterior cingulate or gyrus cinguli anteriors or cinguli anterior gyrus or superior mesial region or anterior cingulate or gyrus cinguli anterior or cingulate anterior or region cingulate or cingulate areas).ti,ab.

12. (Parahippocampal Gyrus or gyrus parahippocampal or gyri parahippocampal or parahippocampal gyri posterior or hippocampal gyrus or gyri posterior parahippocampal or posterior parahippocampal gyrus or gyrus parahippocampalis or parahippocampal gyrus uncus or presubiculums or posterior parahippocampal gyri or gyrus posterior parahippocampal or parahippocampal gyrus posterior or uncus of parahippocampal gyrus or gyri hippocampal or parahippocampal gyrus or presubiculum or gyrus hippocampi or uncus parahippocampal gyrus or gyrus uncus parahippocampal or gyrus hippocampal or parahippocampal gyri or Entorhinal Cortex or area entorhinali or areas entorhinal or entorhinalis area or entorhinal area or area entorhinal or entorhinal cortices or area entorhinalis or cortices entorhinal or entorhinali area or olfactory cortices secondary or secondary olfactory cortex or cortex secondary olfactory or cortices secondary olfactory or entorhinal cortex or olfactory cortex secondary or secondary olfactory cortices or cortex entorhinal or entorhinal areas or Olfactory Pathways or olfactory pathways or pathways olfactory or olfactory pathway or pathway olfactory or Perforant Pathway or pathway perforant or pathways perforant or perforant paths or perforant pathways or perforant pathway or fasciculus perforating or paths perforant or perforant path or perforating fasciculus or path perforant or Septum of Brain or paraterminal body or brain septums or brain septum or septum of brain or paraterminal bodies or area septal or bodies paraterminal or body paraterminal or septal area or region septal or septal region or Septal Nuclei or nucleus of the stria terminalis or septi lateralis nucleus or septal nuclear complices or nucleus of anterior commissure or terminali nucleus striae or laterali nucleus septalis or nucleus lateralis septi or nucleus lateralis septus or nuclear complices septal or septofimbrial nucleus or diagonal band nucleus or nucleus septofimbrial or nucleus septi lateralis or laterali nucleus septi or nucleus triangular septal or medial septal nucleus or nucleus of diagonal band or nucleus septalis lateralis or nucleus striae terminali or nuclear complex septal or septum nucleus lateral or lateral septal nucleus or lateralis nucleus septalis or septal nucleus lateral or septalis laterali nucleus or nuclei septal or anterior commissure nucleus or septus nucleus lateralis or septi laterali nucleus or nucleus medial septum or nucleus septalis laterali or triangularis septus nucleus or lateralis nucleus septi or complex septal nuclear or nucleus striae terminalis or nucleus of stria terminalis or septalis lateralis nucleus or dorsal septal nucleus or nucleus triangularis septus or nucleus lateral septumor nucleus triangularis septi or nucleus lateral septal or septal nucleus triangular or terminalis nucleus striae or septi nucleus lateralis or septi nucleus triangularis or triangular septal nucleus or septus nucleus triangularis or nucleus medial septal or complices septal nuclear or lateralis septus nucleus or medial septum nucleus or lateralis septi nucleus or Substantia Innominata or innominata substantia or substantia innominata or Prosencephalon or prosencephalon or forebrains or forebrain or Diencephalon or diencephalon or interbrain or interbrains or Optic Chiasm or chiasmas optic or optic chiasms or decussation optic or chiasma optic or optic chiasm or optic chiasma or optic decussation or opticums chiasma or optic decussations or chiasma opticum or decussations optic or opticum chiasma or optic chiasmas or chiasma opticums or chiasms optic or chiasm optic or Optic Tract or Subthalamus or subthalamus or fasciculus thalamic or field h nucleus or campi forelus nucleus or fasciculus lenticular or field h1 forel's or campi foreli nucleus or enticular fasciculus or forels field h2 or forel field h2 or thalamicus fasciculus or fasciculus thalamicus or thalamic fasciculus or forelus nucleus campi or nucleus of ansa lenticularis or foreli nucleus campi or nucleus campi forelus or nucleus of field h or forels field h1 or forel's field h2 or field h1 of forel or forel field h1 or Entopeduncular Nucleus or Subthalamic Nucleus or nucleus of luys or luys subthalamic nucleus or corpus luysi or luys body or subthalamic nucleus of luys or subthalamicus nucleus or luys nucleus or nucleus subthalamic or luysi corpus or body of luys or nucleus subthalamicus or subthalamic nucleus or Zona Incerta or Thalamus or thalamencephalon or thalamencephalons or thalamus or Thalamic Nuclei or nuclei thalamic or thalamic nuclei or Anterior Thalamic Nuclei or nucleus anterodorsal thalamic or anterior nuclear group or nucleus anteromedial thalamic or nucleus anteroventral thalamic or thalamus anterior nucleus or anterior thalamic nucleus or nucleus anteroventral or anteroventral nucleus or thalamic nucleus anterodorsal or nuclei anterior thalamic or thalamic nuclei anterior or anteromedial nucleus or anteromedial thalamic nucleusor thalamus anterior or nucleus anteromedial or anterodorsal nucleus or anterior thalamus or anterior thalamic nuclei or anterodorsal thalamic nucleus or nucleus anterodorsal or thalamic nucleus anteroventral or Geniculate Bodies or nucleus geniculate or medial geniculate nucleus or geniculate complex medial or geniculatum mediales corpus or bodies geniculate or nucleus lateral geniculate or mediales corpus geniculatum or geniculate bodies medial or mediale corpus geniculatum or geniculate body or geniculatum mediale corpus or geniculate nucleus lateral or geniculate bodies or geniculate bodies lateral or metathalamus or corpus geniculatum mediale or geniculate body lateral or complex medial geniculate or nucleus geniculatus lateralis pars dorsalis or geniculate body medial or geniculate complices medial or geniculate nucleus or complices medial geniculate or medial geniculate body or medial geniculate bodies or geniculate nucleus medial or Intralaminar Thalamic Nuclei or nucleus paracentrali or centrum medianum or paracentrali nucleus or centromedian thalamic nucleus or central lateral nucleus or thalamic nucleus parafascicular or central lateral thalamic nucleus or parafascicular thalamic nucleus or thalamic nucleus intralaminar or nucleus central dorsal or parafascicular nucleus of the thalamus or centromedian nucleus or intralaminar nuclei rostral or intralaminar nuclear group or thalamic nucleus centromedian or parafascicularis nucleus or nucleus central lateral or thalamic nuclei intralaminar or central dorsal thalamic nucleus or interlaminar nuclei of thalamus or rostral intralaminar nuclei or thalamus nucleus parafascicularis or centrum medianums nucleus or medianum centrum or thalamic nucleus paracentral or thalamus reticulate nucleus or nucleus paracentral or nucleus central medial or paracentral thalamic nucleus or median nucleus centre or nuclei intralaminar thalamic or nuclei rostral intralaminar or central medial nucleus or nucleus centrum medianums or nucleus centre median or medianum nucleus centrum or nucleus paracentral thalamic or nucleus centromedian thalamic or nucleus parafascicularis thalamus or nucleus intralaminar thalamic or nucleus centrum medianum or nucleus parafascicularis thalami or parafascicularis thalami nucleus or parafascicularis thalamus nucleus or reticulate nuclei of thalamus or nucleus parafasciculari or centrum medianums or centrum medianum nucleus or paracentralis nucleus or lateral nucleus central or parafascicular nucleus or central medial thalamic nucleus or nucleus centromedian or Lateral Thalamic Nuclei or medial pulvinar nucleus or Pulvinar or anterior pulvinar nucleus).ab,ti.

13. (pulvinar nucleus or nucleus anterior pulvinar or lateral pulvinar nucleus or pulvinar nucleus oral or oral pulvinar nucleus or pulvinar nucleus inferior or pulvinars or pulvinari nucleus or nucleus oral pulvinar or nucleus pulvinar or pulvinar thalami or nucleus pulvinari or nucleus lateral pulvinar or thalami pulvinar or nucleus inferior pulvinar or pulvinaris nucleus or nucleus pulvinaris or pulvinar thalamus or inferior pulvinar nucleus or pulvinar nucleus lateral or Mediodorsal Thalamic Nucleus or medialis dorsali nucleus or medial dorsal thalamic nucleus or mediodorsal nucleus or dorsomedialis thalamus nucleus or nucleus dorsomedial thalamic or nuclei medial thalamic or dorsali nucleus medialis or nucleus mediodorsal or nucleus dorsomedialis thalamus or nucleus medialis dorsali or nucleus mediodorsal thalamic or thalami nucleus dorsomedialis or thalamus nucleus dorsomedialis or thalamic nucleus medial or dorsomedialis thalami nucleus or thalamic nuclei medial or nucleus medial thalamic or mediodorsal thalamic nucleus or nucleus dorsomedial or nucleus medialis dorsalis or thalamic nucleus mediodorsal or dorsal medial nucleus or medialis dorsalis nucleus or nucleus dorsomedialis thalami or medial thalamic nucleus or Midline Thalamic Nuclei or parataenial nucleus or nucleus reunien or rhomboid nucleusor nucleus subfascular or nuclear group midline or paratenial nucleus or rhomboidal nucleus or rhomboid thalamic nucleus or nucleus rhomboid thalamic or reuniens nucleus or subfascular nucleus or thalamus nucleus reuniens or thalami nucleus reuniens or nucleus paraventricular thalamic or reunien nucleus or reuniens thalami nucleusor paraventricular nucleus of thalamus or paraventricular thalamic nucleus or midline thalamic nucleus or thalamic nuclei midline or paratenial thalamic nucleus or thalamic nucleus rhomboid or periventricular nuclei of thalamus or thalamic nucleus reuniens or reuniens thalamus nucleus or thalamus midline nucleus or nucleus paratenial or thalamus paraventricular nucleus or midline thalamic nuclei or thalamic nucleus subfascular or nucleus reuniens thalamus or thalamic nucleus paratenial or nucleus reuniens or nucleus rhomboid or thalamic nucleus paraventricular or midline nuclear group or Posterior Thalamic Nuclei or supergeniculate nucleus or posterior nuclear complicesor nucleus supergeniculateor posterior thalamic nuclei or suprageniculate thalamic nucleus or submedial nucleus or limitans nucleus or thalamic nuclei posterior or nucleus limitan or thalamic nucleus suprageniculate or nucleus submedial or nuclear complices posterior or complices posterior nuclear or posterior nucleus of thalamus or nucleus limitans or nucleus suprageniculate thalamic or posterior thalamic nucleus or posterior thalamic nuclear group or posterior nuclear complex or nuclear complex posterior or thalamus posterior nucleus or Ventral Thalamic Nuclei or posterior nucleus ventral or ventrolateral thalamic nucleus or intermedius nucleus ventralis or ventral posterior nucleus or ventralis intermedius nucleus or ventrobasal complex or ventralis posteromediali nucleus or nuclear mass ventral or group ventral nuclear or ventralis posterolateralis nucleus or nucleus ventral anterior or ventral nuclear groups or laterali nucleus ventralis or nucleus ventral posterolateral or ventralis posterior nucleus or masses ventral nuclear or nucleus ventralis posterolaterali or ventral lateral nucleus or nucleus ventralis intermedius or ventral anterior thalamic nucleus or thalamic nucleus ventral or posterolaterali nucleus ventralis or ventral posteromedial thalamic nucleus or nucleus ventrolateralis thalamus or ventrobasal complices or nucleus ventralis posteromedialis or nuclei ventral thalamic or nucleus ventrolateralis thalami or mass ventral nuclearor ventrolateralis thalami nucleus or ventrolateralis thalamus nucleus or posterolateral nucleus ventral or nuclear group ventral or arcuate nucleus 3 or nucleus ventralis posteriors or ventral posterior thalamic nucleus or ventral posterior medial nucleus or ventral posteroinferior nucleus or posteroinferior nucleus ventral or posteriors nucleus ventralis or arcuate nucleus-3 or thalamus nucleus ventrolateralis or nucleus ventralis or posteromediali or complex ventrobasal or ventral lateral thalamic nucleus or ventral thalamic nuclei or ventral lateral thalamic nuclei or ventral posteromedial nucleus or posteromedialis nucleus ventralis or ventral anterior nucleus or ventral posterolateral nucleus or nuclear masses ventral or ventral posterior inferior thalamic nucleus or thalamic nucleus ventrolateral or ventral nuclear group or thalamus ventrolateral or thalami nucleus ventrolateralis or posteromediali nucleus ventralis or posterolateralis nucleus ventralis or posterior nucleus ventralis or nucleus ventral posteromedial or nucleus ventralis laterali or ventral posterolateral thalamic nucleus or nucleus ventral thalamic or ventralis lateralis nucleus or ventral nuclear mass or ventralis posteriors nucleus or ventralis laterali nucleus or nucleus ventral posterior or ventral thalamic nucleus or ventrolateral thalamus or nucleus ventralis lateralis).ab,ti.

14. (Telencephalon or telencephalon or endbrain or endbrains or Cerebrum or cerebrum or cerebral hemisphere left or cerebral hemisphere right or cerebral hemispheres or right cerebral hemisphere or cerebral hemisphere or left cerebral hemisphere or Basal Ganglia or ganglia basal or nuclei basal or basal ganglia or ganglion basal or basal nuclei or claustrum or Corpus Striatum or lenticular nucleus or nucleus lentiform or lentiformis nucleus or lentiform nucleus or corpus striatum or nucleus lenticular or nucleus lentiformis or lentiform nuclei or striatum corpus or nuclei lentiform or Globus Pallidus or pallidum or paleostriatum or globus pallidus or pallidums or Neostriatum or Caudate Nucleus or nucleus caudatus or caudate nucleus or caudatus nucleusor nucleus caudate or caudatus or High Vocal Center or Putamen or putamens or nucleus putamens or putamens nucleus or putamen nucleus or nucleus putamen or putamen or Ventral Striatum or Nucleus Accumbens or nucleus accumbens or accumbens septus nucleus or accumbens septi nucleusor nucleus accumbens septi or septi nucleus accumbens or accumbens nucleus or septus nucleus accumbens or nucleus accumbens septus or Olfactory Tubercleor Islands of Calleja or Basal Nucleus of Meynert or nucleus basalis of meynert or meynert basal nucleus or nucleus basalis magnocellularis or basal nucleus of meynert or meynert nucleus basalisor Cerebral Cortex or plates cortical or insular cortex or cerebral cortices or archipalliums or paleocortex or allocortices or periallocortices or plate cortical or cerebri cortex or cortices cerebral or paleocortices or cortices insular or insular cortices or cortex insular or periallocortex or archipallium or cortical plates or cortex cerebral or cortex cerebri or reil insula or cortex cerebrus or cortical plate or Frontal Lobe or gyrus anterior centralor central gyrus anterior or lobe frontalor frontal lobeor cortex frontal or gyrus precentralis or frontal eye fieldor supplementary eye field or gyrus precentrali or frontali lobusor precentrali gyrus or frontal lobes or frontal cortex or field supplementary eye or lobes frontal or eye field supplementary or lobus frontali or supplementary eye fields or frontalis lobus or gyrus precentral or eye fields supplementary or eye fields frontal or anterior central gyrus or fields frontal eye or lobus frontalis or Motor Cortex or motor area or primary motor cortex or motor area precentral or strip motor or somatomotor areas or strips motor or motor cortices primary or premotor areas or motor area secondary or cortex precentral motor or motor area somatic or supplementary motor areas or area primary motoror area premotor or secondary motor area or motor cortices secondary or area motor or secondary motor areas or area somatomotor or motor areas or motor cortex secondary or precentral motor areas or cortices secondary motor or area supplementary motor or motor areas supplementary or area precentral motor or cortices primary motor or precentral motor cortices or areas somatic motor or area somatic motor or areas motor or motor cortex precentral or motor areas precentral or motor strips or cortex primary motor or somatomotor area or premotor area or precentral motor cortex or primary motor area or somatic motor area or motor areas somatic or areas premotor or areas somatomotor or areas precentral motor or areas supplementary motor or motor cortex primary or cortex secondary or motor primary motor cortices or motor cortex or motor cortices precentral or motor area supplementary or cortices precentral motor or somatic motor areas or cortex motor or areas secondary motor or Prefrontal Cortex or orbital gyrus).ab,ti.

15. (gyrus orbital or sulcus olfactoryor convolutions superior frontal or orbitofrontal cortices lateral or gyrus frontalis superior or rectal gyrusor cortices ventromedial prefrontal or orbital cortices or cortex orbital or prefrontal cortices ventromedial or inferiors gyrus frontalis or orbital gyri or orbital area or convolution superior frontal or frontalis superiors gyrus or inferior frontal gyrus or gyri orbitofrontal or orbitofrontal regions or frontalis inferiors gyrus or frontal sulcus or prefrontal cortex ventromedial or straight gyrus or cortex lateral orbitofrontal or gyrus frontalis inferior or sulci olfactory or orbital areas or orbitofrontal gyri or area orbital or orbitofrontal region or cortices lateral orbitofrontal or lateral orbitofrontal cortex or superior frontal convolution or cortex orbitofrontal or medial frontal gyrus or gyrus orbitofrontal or gyrus straight or superior frontal gyrus or frontal gyrus medial or ventromedial prefrontal cortex or gyrus rectal or subcallosal area or olfactory sulcus or prefrontal cortex or superior frontal convolutions or sulcus frontal or olfactory sulci or region orbitofrontal or superiors gyrus frontalis or superior gyrus frontalis or gyrus superior frontal ororbitofrontal cortex or frontal gyrus inferior or gyrus frontalis inferiors or cortex ventromedial prefrontal or marginal gyrus or rectus gyrus or orbital cortex or gyrus medial frontal or orbitali gyrus or orbitofrontal gyrus or inferior gyrus frontalis or frontal gyrus superior or gyri orbital or areas orbital or cortex prefrontal or cortices orbital or gyrus rectus or frontalis superior gyrus or lateral orbitofrontal cortices or orbitofrontal cortices or gyrus frontalis superiors or orbitofrontal cortex lateral or gyrus marginal or Broca Area or Neocortex or neocortical molecular layer or neocortices cerebral or isocortex or cerebral neocortices or neopalliumsor corticalis substantiaor multiform layer neocortical or neocortical multiform layer or layer neocortical molecular or cortices neopallial or neopallial cortex or neocortical internal pyramidal layer or molecular layer neocortical or neopallial corticesor cortex neopallial or layers neocortical multiform or neocortex cerebralor molecular layers neocortical or neocortical internal granular layer or neocortical multiform layers or cerebral neocortex or neocortical external pyramidal layer or neocortical molecular layers or isocortices or external granular layer or substantia corticali or corticali substantia or layer neocortical multiformor multiform layers neocortical or Occipital Lobe or occipital cortex or cuneus or gyrus annectant or sulcus lunate or gyrus lingual or gyrus occipitalor calcarine fissures or regions occipital or occipitotemporal gyrus medial or sulcus calcarine or lunate sulcus or occipital lobe or gyrus medial occipitotemporal or cuneate lobule or region occipital or fissures calcarine or calcarinus sulcus or sulcus calcarinus or lobe occipital or lobes occipital or occipital region or gyrus lingualis or occipital gyrus or annectant gyrus or occipital regions or lobules cuneate or occipital sulcus or cortices occipital or calcarine sulcus or fissure calcarine or lingual gyrus or lobule cuneate or cortex cuneus or linguali gyrus or Visual Cortex or primary visual cortices or visual cortex primaries or primaries visual cortex or cortices extrastriate or visual cortices primary or extrastriate cortices or cortex primaries visual or cortex primary visual or striate cortex or cortices primary visual or visual cortex primary or visual cortex or cortex striate or cortex extrastriate or cortex visual or extrastriate cortex or primary visual cortex or Olfactory Cortex or Basal Forebrain or Piriform Cortex or sulcus intraparietal or regions parietal or lobes parietal or paracentral lobules posterior or gyrus supramarginal or precuneus cortices or parietal cortex or gyrus angulari or praecuneus or gyrus angularis or gyrus supramarginali or parietal regions or gyrus prelunate or lobules parietal or lobe parietal or parietal cortices posterior or gyrus supramarginalis or angulari gyrus or supramarginali gyrus or marginal sulcus or posterior parietal cortex or prelunate gyrus or posterior parietal cortices or intraparietal sulcus or angularis gyrus or region parietal or parietal lobules or precuneus or cortex parietal or gyrus angular or precuneus cortex or lobule parietal or parietal lobule or cortices precuneus or posterior paracentral lobule or lobules posterior paracentral or sulcus marginal or posterior paracentral lobules or Parietal Lobe or sulcus intraparietal or regions parietal or lobes parietal or paracentral lobules posterior or gyrus supramarginal or precuneus cortices or parietal cortex or gyrus angulari or praecuneus or gyrus angularis or gyrus supramarginali or parietal regions or gyrus prelunate or lobules parietal or lobe parietal or parietal cortices posterior or gyrus supramarginalis or angulari gyrus or supramarginali gyrus or marginal sulcus or posterior parietal cortex or prelunate gyrus or posterior parietal cortices or intraparietal sulcus or angularis gyrus or region parietal or parietal lobules or precuneus or cortex parietal or gyrus angular or precuneus cortex or lobule parietal or parietal lobule or cortices precuneus or posterior paracentral lobule or lobules posterior paracentral or sulcus marginal or posterior paracentral lobules or omatosensory Cortex or postcentral gyrus or somatosensory cortices primary or somatosensory cortex primary or cortex anterior parietal or cortices anterior parietal or cortices primary somatosensory or anterior parietal cortices or cortex secondary sensory or areas primary somatosensory or primary somatosensory cortices or cortex si or gyrus post central or secondary somatosensory areas or post central gyrus or parietal cortices anterior or gyrus postcentrali or secondary somatosensory cortex or somatosensory cortex or areas secondary somatosensory or somatosensory cortex secondary or area primary somatosensory or postcentralis gyrus or cortices secondary sensory or secondary sensory cortex or primary somatosensory cortex or somatosensory areas secondary or si cortex or primary somatosensory areas or secondary somatosensory cortices or gyrus postcentralis or area secondary somatosensory or primary somatosensory area or cortex primary somatosensory or secondary somatosensory area or secondary sensory cortices or cortices secondary somatosensory or primary somatic sensory area or postcentrali gyrus or gyrus postcentral or Wernicke Area or Sensorimotor Cortex or Auditory Cortex or gyrus transverse temporal or auditory areas temporal or temporal auditory areas or areas auditory or cortex primary auditory or transverse temporal gyrus or cortex auditory or auditory areas or transverse temporal gyri or convolutions heschl's or auditory cortex or auditory area or auditory cortex primary or auditory cortices primary or areas temporal auditory or area auditory or gyri transverse temporal or primary auditory cortices or heschl gyri or heschl convolutions or temporal gyri transverse or auditory area temporal or temporal auditory area or heschls gyri or primary auditory cortex or Temporal Lobe or temporal operculums or superior temporal gyrus or occipito-temporal gyrus lateral or gyrus fusiform or gyrus lateral occipito-temporal or gyrus temporalis superior or occipitotemporal gyrus or horns temporal or inferior horn of lateral ventricle or temporal sulcus or regions temporal or operculums temporal or cortex temporal or lobes temporal or planum polares or temporalis superior gyrus or gyrus superior temporal or horn temporal or lobe temporal or temporal horn or temporal cortices or gyrus lateral occipitotemporal or temporalis superiors gyrus or region temporal or fusiformi gyrus or temporal region or temporal horns or lateral occipito-temporal gyrus or temporal cortex or gyrus temporal or temporal operculum or temporal regions or operculum temporal or cortices temporal or temporal horn of the lateral ventricle or polare planum or fusiformis gyrus or sulcus temporal or polares planum or planum polare or temporal lobe or Diagonal Band of Broca or diagonal band of broca or broca diagonal band or External Capsule or olfactory tracts or olfactory tract lateral or main olfactory bulbs or bulbs main olfactory or bulb olfactory or glomerulus olfactory or lateral olfactory tracts or olfactory tract or bulb main olfactory or olfactory bulbs or olfactory bulb main or accessory olfactory bulb or accessory olfactory bulbs or tracts olfactory or bulbs accessory olfactory or olfactory glomerulus or bulbs olfactory or olfactory bulb accessory or tract olfactory or bulb accessory olfactory or tract lateral olfactory or olfactorius bulbus or olfactory bulb or lateral olfactory tract or Olfactory Bulb or olfactory tracts or olfactory tract lateral or main olfactory bulbs or bulbs main olfactory or bulb olfactory or glomerulus olfactory or lateral olfactory tracts or olfactory tract or bulb main olfactory or olfactory bulbs or olfactory bulb main or accessory olfactory bulb or accessory olfactory bulbs or tracts olfactory or bulbs accessory olfactory or olfactory glomerulus or bulbs olfactory or olfactory bulb accessory or tract olfactory or bulb accessory olfactory or tract lateral olfactory or olfactorius bulbus or olfactory bulb or lateral olfactory tract or Telencephalic Commissures or Anterior Cerebellar Commissure or Corpus Callosum or corpus callosums or commissures neocortical or neocortical commissures or corpus callosum or callosums corpus or interhemispheric commissure or interhemispheric commissures or neocortical commissure or callosum corpus or commissures interhemispheric or commissure interhemispheric or commissure neocortical or Internal Capsule or internal capsules or interna capsula or capsules internal or capsule internal or capsula internas or internal capsule or capsula interna or internas capsula or Myelencephalon).ab,ti.

16. 1 or 2 or 3 or 4 or 5 or 6 or 7 or 8 or 9 or 10 or 11 or 12 or 13 or 14 or 15

17. exp Alzheimer disease/ or exp tau proteins/ or exp amyloid plaque/ or exp amyloid beta protein/ or exp neurofibrillary tangles/ or exp senile plaques/ or exp amyloid precursor protein/ or exp beta amyloid/

18. alzheimer*.ti,ab.

19. ase.ti,ab.

20. ((mild adj2 cognitive adj2 impairment) or MCI).ti,ab.

21. (cognitive adj2 decline).ti,ab.

22. AD.ti,ab.

23. ((sporadic ADJ2 Alzheimer*) or (sporadic ADJ2 AD) or (early onset ADJ2 Alzheimer*) or (early onset ADJ2 AD) or familial Alzheimer* or (familial ADJ2 Alzheimer*) or (familial ADJ1 AD) or early onset AD or late onset AD or (late onset ADJ2 Alzheimer*) or (late onset ADJ2 Alzheimer disease) or (late onset ADJ2 AD) or EOAD or LOAD or autopsy confirmed Alzheimer* or (autopsy ADj2 Alzheimer*)).ti,ab.

24. ((dementia ADJ4 Alzheimer*) or (senile dementia ADJ4 Alzheimer*) or (dementia ADJ4 AD) or (senile dementia ADJ4 AD) or Alzheimer sclerosis or Alzheimer syndrome or (focal onset ADJ2 Alzheimer*) or (focal onset ADJ2 AD) or (presenile dementia) or (presenile dementia ADJ4 Alzheimer) or (presenile dementia ADJ4 AD) or beta amyloid plaque* or beta-amyloid plaque* or amyloid beta or beta amyloid or beta-amyloid or Abeta or amyloid deposit* or amyloid plaque* or neuritic plaque* or senile plaque* or Alzheimer* disease associated protein* or AD-AP or ADAP or AD-AP or (beta protein ADJ3 Alzheimer*) or (amyloid beta ADJ3 Alzheimer*) or (amyloid-beta ADJ3 Alzheimer*) or amyloid fibril protein* or amyloid beta peptide* or amyloid beta protein* or amyloid-beta peptide* or amyloid-beta protein* or neurofibrillary tangle*or tau protein* or taupath* or NFT or (tangle* ADJ3 Alzheimer*) or tau or neurofibrillary pathology* or Alzheimer disease-associated neurofibrillary pathology or braak staging or (braak ADJ3 Alzheimer*) or CERAD or (CERAD ADJ3 Alzheimer*)).ti,ab.

25. 17 or 18 or 19 or 20 or 21 or 22 or 23 or 24

26. exp fatty acids/ or exp arachidonic acid/ or exp prostaglandins/

27. ((Arachidonic Acid ADJ 2 metabolite*) or ARA metabolite* or AA metabolite* or Arachidonic Acid or ARA or AA or eicosaHydroxyeicosatetraenoic acid* or eicosa-Hydroxyeicosatetraenoic acid* or Hydroxyeicosatetraenoic acid* or HETE or 5-HETE or 5HETE or 5 HETE or 12-HETE or 12HETE or 12 HETE or 8-HETE or 8HETE or 8 HETE or 9-HETE or 9HETE or 9 HETE or 15-HETE or 15HETE or 15 HETE or 20-HETE or 20HETE or 20 HETE or 19-HETE or 19HETE or 19 HETE or 5-Hydroxyeicosatetraenoic acid* or 15-Hydroxyeicosatetraenoic acid* or 12-Hydroxyeicosatetraenoic acid* or 9-Hydroxyeicosatetraenoic acid* or 8-Hydroxyeicosatetraenoic acid*  20-Hydroxyeicosatetraenoic acid or docosanoid* or Eicosanoid* or 19-Hydroxyeicosatetraenoic acid*).ab,ti.

28. (Leukotriene* or LTA4 or LTB4 or LTC4 or LTD4 or LTE4 or LTX4 or LTA5 or LTB5 or LTC5 or LTD5 or LTE5).ab,ti.

29. (Eoxin* or EXA4 or EXC4 or EXD4 or EXE4 or Prostanoid* or Prostaglandin* or PG* or PGG2 or PGH2 or PGE2 or PGD2 or PGF2* or PGA* or PGA1 or PGA2 or PGB2 or Prostacyclin* or Dihomo-2-series prostanoid* or prostaglandin D1 or prostaglandin D2 or prostaglandin D3 or PGD1 or PGD3 or PGD* or dihomo-prostaglandin* or dihomo-PGD2 or dihomo-PGE2 or dihomo-PGI2 or PGI2 or PGI3 or PGH2 or PGE1 or PGE3 or PGE* or Cyclopentenone prostaglandin* or delta12-PGJ* or 15-deoxy-delta 12,14-PGJ2 or dihomo-gamma-linolenic acid* or PGF3* or 15-keto-PGE* or 6-keto PGF1* or PGI3 or Thromboxane* or TXA2 or TXB2 or TXA1 or TXA3 or txb3).ab,ti.

30. ((EPA ADJ2 Oxylipin*) or (eicosapentaenoic acid ADJ2 oxylipin*) or Resolvin* or resolvin D1 or resolvin D2 or D series resolvin* or RVD* or RvE* or 18S-RvE1 or hydroxyeicosatrienoic acid* or 5-hydroxyeicosatrienoic acid* or 12-hydroxyeicosatrienoic acid* or 15-hydroxyeicosatrienoic acid* or 5-HETrE or 15-HETrE or 12-HETrE or 5-oxo-ETrE or 15-deoxy- PGJ2 or 15-deoxy-Prostaglandin*).ab,ti.

31. (Oxoeicosanoid* or Hydroperoxyeicosatetraenoic acid* or Hydroperoxy-eicosatetraenoic acid* or eicosatetraenoic acid* or hPETE* or 5-Hydroperoxyeicosatetraenoic acid* or 5-Hydroperoxy-eicosatetraenoic acid* or 5-HpETE or 5HPETE or 5 HPETE or 5-oxo-ETE or 8-hpete or 12-hpete or 15-hpete or 8hpete or 12hpete or 15hpete or 8 hpete or 12 hpete or 15 hpete or 12-oxo-ETE or 15-oxo-ETE or 5-hydroxy-eicosatetraenoic acid* or 12-hydroxy-eicosatetraenoic acid* or 8-hydroxy-eicosatetraenoic acid* or 15-hydroxy-eicosatetraenoic acid* or oxo-eicosatetraenoic acid* or oxo-ETE* or 5-oxo-eicosatetraenoic acid* or 15-oxo-eicosatetraenoic acid* or 12-oxo-eicosatetraenoic acid* or dihydroxy derivatives or 5,15-dihydroxy-eicosatetraenoic acid or 5,15-DiHETE or 8,15-dihydroxy-eicosatetraenoic acid or 8,15-DiHETE or 12,20-dihydroxy-eicosatetraenoic acid or 12,20-DiHETE or 5-LOX, 5-lypoxygenase activating protein or FLAP or Hepoxilin* or HxA3 or HxB3 or 12-HpETE or 8,9-dihydroxy-eicosatetraenoic acid or 8,9-DiHETE or 14,15-dihydroxy-eicosatetraenoic acid or 14,15-DiHETE or 17,18-dihydroxy-eicosatetraenoic acid or 7,18-DiHETE).ab,ti.

32. (Lipoxin* or LXA* or LXB* or (hydroperoxy ADJ1 acid*) or Epi-lipoxin* or 15-Epi-lipoxin* or Epilipoxin* or 15- Epilipoxin* or 15-epi-LxA4 or AT-LxA4 or 15-epi-LxB4 or AT-LxB4 or Epoxyeicosatrienoic acid* or Epoxy-eicosatrienoic acid* or epoxyeicosatrienoate or epoxy-eicosatrienoate or EET* or 5,6-EET or 8,9-EET or 11,12-EET or 14,15-EET or 5,6-EEQ or 8,9-EEQ or 11,12-EEQ or 14,15-EEQ or 15,16-EEQ or Isoprostane* or isoPs or PG-like ADJ1 compound* or PG-like ADJ1 product* F_2_-isoprostane* or F_2_-IsoPs or F_4_-neuroprotane* or F_4_-NPs D2-isoPs or E2-isoPs or A2-isoPs or J2-isoPs or 5,6-epoxyisoprostane E2 or 5,6-epoxyisoprostane A2 or Isofuran* or dihydroxyeicosatrienoic or DHET* or soluble hydroxylase or 5-oxo-eicosatetraenoic acid or 5-oxo-ete).ab,ti.

33. ((Linoleic acid ADJ2 Oxylipin*) or oxylipin* or OXLAM* or oxidized Linoleic Acid metabolites or  oxidized LA metabolites or ARA oxylipin* or (Arachadinoic Acid ADJ2 Oxylipin*) or AA oxylipin* or DHA oxylipin or (Docosahexaenoic acid ADJ2 oxylipin*) or COX oxylipin* or LOX oxylipin* or CYP oxylipin* or protectin* or NPD1 or neuroprotectin* or 17-epi-PD1 or maresin* or mar1 or mar2 or 7-epi Maresin* or 7-epi-mar1 or mar-l1 or mar-l2 or marl1 or marl2 or Oxylipin* or DHA oxylipin* or eicosapentaenoic acid- derived RvE1 or EPA-derived RvE1 or hydroxydocosahexaenoic acid* or HDoHE or protectin D1 or PD1 or linoleic acid-derived 9-hydroxyoctadecadienoic acid* or LNA-derived 9-hydroxyoctadecadienoic acid* or linoleic acid-derived 13-hydroxyoctadecadienoic acid* or LNA-derived 13-hydroxyoctadecadienoic acid* or hydroxyoctadecadienoic acid* or HODE or 9-HODE or 13-HODE or 9,10-dihydroxy-octadecenoic or 13-oxo-octadecadienoic acid* or 9-oxo-octadecadienoic acid* or 13-oxo-ODE or 9-oxo-ODE or 9-HpODE or 9-hydroperoxy-10E,12Z-octadecadienoic acid or 13-HpODE or 13-hydroperoxy-10E,12Z-octadecadienoic acid).ab,ti.

34. (Hydroxy Fatty acid*or hydroxyl FA or 11-HETE or 11HETE or 11 HETE or 11-oxo-ETE or 13-HDoHE or 13HDoHE or 11-hydroxy-eicosatetraenoic acid* or 13-hydroxy-docosahexaenoic acid* or 9-hydroxy-octadecadienoic acid* or 17-hydroxy Docosahexaenoic Acid* or 17-HDoHE or 17-HDHA or hydroperoxyl-DHA or hydorperoxyl- docosahexaenoic acid or hp-DHA or hpDHA or 17-hydroperoxy-docosahexaenoic acid or 17-hydroperoxydocosahexaenoic acid or hydroxydha or 14-hpdha or 17s-hpdha or 14s-hpdha or hydroperoxy-docosahexaenoic acid or hydroxydocosahexaenoic acid or HDHA or 17-HDHA or 13-oxo-dha or 17-oxo-dha).ab,ti.

35. (epoxy-eicosatrienoic acid* or EpETrE or epoxyeicosatetraenoic acid* or 5,6-epoxy-eicosatrienoic acid* or 5,6-EpETrE or 5,6-epoxyeicosatetraenoic acid* or 8,9- epoxy-eicosatrienoic acid* or 8,9-EpETrE or 8,9-epoxyeicosatetraenoic acid* or 11,12-epoxy-eicosatrienoic acid* or 11,12-EpETrE or 11,12-epoxyeicosatetraenoic acid* or 14,15-epoxy-eicosatrienoic acid* or 14,15-EpETrE or 14,15-epoxyeicosatetraenoic acid* or epoxy-eicosatetraenoic acid* or EpETE or EpDPE or EDP or HETE or hydroxyeicosapentaenoic acid* or hydroxy-eicosapentaenoic acid* or hydroxy eicosapentaenoic acid* or HEPE* or 5-hepe or 12-hepe or 15-hepe or 20-hepe or 5hepe or 12hepe or 15hepe or 20hepe or 8-HEPE or 8HEPE or 8 HEPE or 9-HEPE or 9HEPE or 9 HEPE or 18-HEPE or 18HEPE or 18 HEPE or HDoHE or dihydroxy-eicosatrienoic acid* or dihydroxyeicosatrienoic acid* or DiHETrE or 5,6-dihydroxy-eicosatrienoic acid* or 5,6-dihydroxyeicosatrienoic acid* or 8,9-dihydroxy-eicosatrienoic acid* or 8,9-dihydroxyeicosatrienoic acid* or 11,12-dihydroxy-eicosatrienoic acid* or 11,12-dihydroxyeicosatrienoic acid* or 14,15-dihydroxy-eicosatrienoic acid* or 14,15-dihydroxyeicosatrienoic acid* or 8,9-DiHETrE or 5,6-DiHETrE or 11,12-DiHETrE or 15,16-DiHETrE or DiHETE or dihydroxy-docosapentaenoic acid* or dihydroxydocosapentaenoic acid* or 5-oxo- eicosapentaenoic acid* or 5-oxo-EPE or oxo-eicosapentaenoic acid* or oxo-EPE or hydroperoxy-eicosapentaenoic acid* or HpEPE* or 12-hydroperoxy-eicosapentaenoic acid* or 15-hydroperoxy-eicosapentaenoic acid* or 12-HpEPE or 15-HpEPE).ab,ti.

36. (dihydroxy-octadecatrienoic acid* or dihydroxyoctadecatrienoic acid* or dihydroxy octadecatrienoic acid* or diHOTrE or hydroxy-octadecatrienoic acid* or hydroxyoctadecatrienoic acid* or hydroxy octadecatrienoic acid* or HOTrE or Hydroperoxy-octadecatrienoic acid* or Hydroperoxyoctadecatrienoic acid* or hydroperoxy octadecatrienoic acid* or HpOTrE or oxo-octadecatrienoic acid* or oxooctadecatrienoic acid* or oxo octadecatrienoic acid* or oxo-OTrE or dihydroxy-octadecadienoic acid* or dihydroxyoctadecadienoic acid* or dihydroxy octadecadienoic acid*).ab,ti.

37. (specialized pro-resolving mediator* or specialized proresolving mediator* or lipid mediator* or specialised pro-resolving mediator* or specialised proresolving mediator*).ab,ti.

38. (Endocannabinoid* or ethanolamide* or n-acylethanolamide* or n acylethanolamide* or fatty acid ethanolamide* or cannabinoid* or endocannabinoid-like or Endocannabinoid system* or cannabinoid receptor* or CB1 or CB2 or Arachidonoylethanolamine* or 2-Arachidonoylglycerol or 2-Arachidonyl glyceryl ether* or O-arachidonoyl-ethanolamine* or N-Arachidonoyl dopamine* or ethanolamine* or aminoethanol* or n-acylethanolamine* or n acylethanolamine* or fatty acid ethanolamine* or Palmitoyl ethanolamide* or Palmitoylethanolamide* or Palmitoylethanolamine* or n-palmitoylethanolamine* or n palmitoylethanolamine* or palmidrol* or Hydroxyethylpalmitamide* or Heptadecanoyl ethanolamide* or Heptadecanoylethanolamide * or Heptadecanoylethanolamine* or n-heptadecanoylethanolamine* or n heptadecanoylethanolamine* or Heptadecanoyl ethanolamine* or Stearoyl ethanolamide* or Stearoylethanolamide* or Stearoylethanolamine* or n-stearoylethanolamine* or n Stearoyl ethanolamine* or Stearoyl ethanolamine* or Oleoyl ethanolamide* or Oleoylethanolamide* or Oleoylethanolamine* or n-oleoylethanolamine* or n Oleoylethanolamine* or Oleoylethanolamine* or Alpha-linolenoyl ethanolamide* or alpha-linolenoylethanolamine* or n-alpha-linolenoyl ethanolamine* or Alpha-linolenoyl ethanolamine* or n alpha-linolenoyl ethanolamine* or alpha-linolenoyl ethanolamine* or Linoleoyl ethanolamide* or Linoleoylethanolamide* or Linoleoylethanolamine* or n-Linoleoylethanolamine* or n Linoleoylethanolamine* or Linoleoyl ethanolamine* or Anandamide* or n-arachidonoylethanolamine* or arachidonoyl ethanolamide* or arachidonoylethanolamide* or Eicosadienoyl ethanolamide* or Eicosadienoylethanolamine* or n arachidonoylethanolamine* or Arachidonoyl ethanolamine* or arachidonoyl glycerol* or Docosatetraenoyl ethanolamide* or Docosatetraenoylethanolamide* or Docosatetraenoylethanolamine* or n-docosatetraenoylethanolamine* or n docosatetraenoylethanolamine* or Docosatetraenoyl ethanolamine* or Eicosapentanoyl ethanolamide* or Eicosapentanoylethanolamide* or Eicosapentanoylethanolamine* or n-Eicosapentanoylethanolamine* or n Eicosapentanoylethanolamine* or Eicosapentanoyl ethanolamine* or Docosapentaenoyl ethanolamide* or Docosapentaenoylethanolamide* or Docosapentaenoylethanolamine* or n-docosapentaenoylethanolamine* or n docosapentaenoylethanolamine* or docosapentaenoyl ethanolamine* or Docosahexanoylethanolamide* or Docosahexanoyl ethanolamide* or Docosahexanoylethanolamine* or n-docosahexanoylethanolamine* or n docosahexanoylethanolamine* or docosahexanoyl ethanolamine* or synaptamide or synaptamine* or dihomo-gama-linolenoyl ethanolamide* or linolenoyl ethanolamide* or inolenoyl ethanolamimine* or dihomo-gama-linolenoyl ethanolamine* or dihomo gamma linolenoyl ethanolamide* or fatty acid amide hydrolase* or faah*).ab,ti.

39. (Phospholipase A2 or PLA2 or sPLA2 or cPLA2 or ipla2 or lp-PLA2 or pla2g4a or pla2g6 or Phosphoinositide phospholipase C or Phospholipase C or PLC or Phospholipase* or Lecithinase* or Lypoxygenase*or LOX*or Arachidonate 5-lipoxygenase* or ALOX5 or 5-lipoxygenase* or 5-LOX or 5-LO or 5LOX or 5LO or 5 LOX or Arachidonate 12-lipoxygenase* or ALOX12 or 12-lipoxygenase or 12-LOX or 12-LO or 12LOX or 12LO or 12 LOX or 15-LOX or 15LOX or 15 LOX or 15-LO or 15LO or Arachidonate 15-lipoxygenase-1 or ALOX15 or 15-lipoxygenase-1 or erythrocyte type 15-lipoxygenase* or 15-lipoxygenase* or reticulocyte type 15-lipoxygenase* or 15-lipoxygenase, reticulocyte type or 15-LO-1 or 15-LOX-1 or 15LO1 or 15LOX1 or Arachidonate 15-lipoxygenase* or Arachidonate 15-lipoxygenase type II or ALOX15B or 15-lipoxygenase-2 15-LOX-2 or 15-LOX-2 or Arachidonate 12-lipoxygenase* or 12R type ALOX12B or 12R-lipoxygenase or 12R-LOX or 12R-LO or platelet-type lipoxygenase 12 or ALOX12 or ALOX15 or ALOX5 or leukocyte-type arachidonate 15-lipoxygenase or Cytochrome p450 or CYP* or cyclooxygenase* or COX2 or COX-2 or COX1 or COX-1 or COX* or prostaglandin-endoperoxide synthase* or Prostaglandin E Synthase 2 or Prostaglandin-E Synthase or PTGS1 or PTGES2 or PGES* or mPGES or cPGES or PTGES* or (Prostaglandin ADJ3 Synthase*) or (5-lypoxygenase activating ADJ1 protein) or FLAP or epoxygenase* or CYP epoxygenase* or Cytochrome P450 omega hydroxylase* or cytochrome P450 omega-hydroxylase* or CYP450 omega hydroxylase* or CYP450 omega-hydroxylase* or CYP omega hydroxylase* or CYP omega-hydroxylase* or fatty acid omega hydroxylase* or cytochrome P450 monooxygenase* or fatty acid monooxygenase* or docosahexaenoic acid omega-hydroxylase* or docosahexaenoate,NADPH:O2 oxidoreductase* or P450 omega-hydroxylase* or thromboxane A synthase* or thromboxane-A synthase* or TBXAS1 or acyltransferase lysolecithin-lysolecithin or l2, lysophospholipase or lecithinase b or lysolecithin lysolecithin acyltransferase* or lysolecithin-lysolecithin acyltransferase or lysophospholipase* or lysophospholipase a or lysophospholipase a1 or lysophospholipase c or lysophospholipase l2 or phospholipase b or phospholipase c or phospholipase*, type c or type c phospholipase* or arachidonate lipoxygenase* or arachidonic acid lipoxygenase* or lipoxygenase* arachidonic acid or lipoxygenase* or Elovanoid*).ti,ab.

40. 26 or 27 or 28 or 29 or 30 or 31 or 32 or 33 or 34 or 35 or 36 or 37 or 38 or 39

41. 16 and 25 and 40

**Results found per database**

Extraction of titles and abstract on January 15, 2020

Medline: 6581

Embase: 15217

PsycINFO: 1634

Total: 23432

Total after EndNote Deduplication: 16279

Total after Covidence deduplication: 16236

Extraction of titles and abstract on August 3, 2021

Medline: 7552

Embase: 17310

PsycInfo: 1837

Total: 26699

Total additional articles added to screening text-abstract after Covidence deduplication: 7011

Extraction of titles and abstract on May 19, 2023

Medline (2021-current): 1568

Embase (2021-current): 4040

PsycInfo (2021-current): 316

Total: 5924

Total additional articles added to screening text-abstract after Covidence deduplication: 3675

**Appendix 4.**

| Reference | Number of cases (Male/Female)  Use of anti-inflammatory if reported | Age | Brain Region | Post-mortem  delay | Brain fixation method | Brain Bank | Methods | Outcomes  (Lipid mediators or enzymes) | Significance |
| --- | --- | --- | --- | --- | --- | --- | --- | --- | --- |
