# Supplementary material for: Lipid mediators in post-mortem brain samples from patients with Alzheimer's disease: A systematic review
Source: Brain Behav Immun Health. 2024 Dec 23;43:100938. doi: 10.1016/j.bbih.2024.100938 (PMC11782888; doi:10.1016/j.bbih.2024.100938)
Supplement: Multimedia component 4 [file mmc4.docx]

**Table 1. Table reporting lipid mediator levels in AD and control brains.**

| **Ref.** | **Diagnosis of AD** | **Number of cases (M,F)** | **Age (Years ± SEM) or [age range]** | **Brain region** | **Post-mortem delay (h ± SEM) or [range]** | **Brain Bank** | **Brain fixation** | **Use of anti-inflammatory** | **Lipid mediator & Methods** | **Expression level in AD** | **% of difference AD vs CTRL** |
| --- | --- | --- | --- | --- | --- | --- | --- | --- | --- | --- | --- |
| **(Bhatia et al., 2013)** | National Institute on Aging-Reagan criteria | AD I/II= 8 (5M,3F); AD III/IV = 7 (1M,6F); AD V/VI = 10 (3M,7F); CTRL = 9 (6M,3F) | AD I/II = 82 ± 6 [62-104]; AD III/IV = 84 ± 4 [67-98]; AD V/VI = 83 ± 4 [67-100]; CTRL = 74 ± 4 [64-93] | Hippocampus; Cerebellum | AD I/II = 26 ± 5 [10-46]; AD III/IV = 27 ± 8 [5-64]; AD V/VI = 11 ± 3 [3-32]; CTRL = 24 ± 5 [6-45] | New South Wales Tissue Resource Centre and the Sydney Brain Bank | Frozen tissue | NA | F2-isoprostanes  **GC-MS** | **Hippocampus:**  F2 IsoPs: ⇔ in AD I/II  F2 IsoPs: ⇑ in AD III/IV;  F2 IsoPs: ⇔ in AD V/VI  **Cerebellum:**  F2 IsoPs: ⇔ in AD I/II  F2 IsoPs: ⇔ in AD III/IV  F2 IsoPs: ⇔ in AD V/VI | **Hippocampus:** I/II: 31.1; III/IV: 51.2*; V/VI: 25.9; **Cerebellum:** I/II: 0.6; III/IV: -29.8; V/VI: -23.4 |
| **(Casadesus et al., 2007)** | Histopathologically confirmed | AD = 21; CTRL = 16 | AD = 80.8 [61-96]; CTRL: 13 cases: 72.9 [60-91]; 3 cases: 17, 23, 43 | Hippocampus (CA1/CA2) | AD = 14.3 [3-37]; CTRL = 19.1 [3-48] | NA | Methacarn fixation | NA | 13,14-dihydro 15-keto PGF2α; 8-iso-PGF2α  IHC | **Pyramidal neurons in hippocampus:**  13,14-dihydro 15-keto PGF2α: ⇑;  8-iso-PGF2α: ⇑ | **Pyramidal neurons in hippocampus:**  **8-iso PGF2a:** 80.8* **13,14-dihydro 15-keto PGF2α:** 93.3* |
| **(Ebright et al. 2022)** | Modified Bielschowsky stain | AD (E3/E3) I/II/IV/V = 12 (7M, 5F)  AD (E3/E4) V = 9 (3M, 6F)  CTRL (E3/E3) II/III/IV/V = 12 (6M, 6F)  CTRL (E3/E4) II/III/IV = 9 (4M, 5F) | AD (E3/E3) I/II/IV/V = 92 ± 6  AD (E3/E4) V = 94 ± 5  CTRL (E3/E3) II/III/IV/V = 85 ± 6  CTRL (E3/E4) II/III/IV = 87 ± 5 | Dorsolateral Prefrontal Cortex | AD (E3/E3) I/II/IV/V = 8  AD (E3/E4) V = 8  CTRL (E3/E3) II/III/IV/V = 8  CTRL (E3/E4) II/III/IV = 8 | NA | Frozen | NA | **ARA-derived lipid mediators:** leukotrienes, prostaglandins, thromboxanes  **SPMs:**  lipoxins, resolvins, neuroproectins, maresins  **LCMS/MS** | **AD 3/3 compared to 3/3 (CTRL)**  **5-HETE:** ⇑  **12-HETE:** ⇓  **15-HETE:** ⇑  **LXA4:** ⇑  **LXB4:** ⇑  **5,6 EET:** ⇑  **8,9 EET:** ⇑  **11,12 EET:** ⇑  **14,15 EET:** ⇑  **LTB4:** ⇑  **PGD2:** ⇑  **PGE2a:** ⇑  **PGF2a:** ⇓  **RvD1/2:** ⇑  **RvD3:** ⇑  **RvD4:** ⇑  **NPD-1:** ⇓  **LXA5:** ⇑  **RvE2:** ⇑  **DPA RvD1:** ⇑ | **% of difference compared to 3/3 (CTRL)**  **5-HETE:**  3/4 (CTRL): -39; 3/3 (AD): 60: 3/4 (AD): 275  **12-HETE:**  3/4 (CTRL): -41; 3/3 (AD): -17: 3/4 (AD): 45  **15-HETE:**  3/4 (CTRL): -39; 3/3 (AD): 14: 3/4 (AD): 141  **LXA4:**  3/4 (CTRL): -67; 3/3 (AD): 137: 3/4 (AD): 463  **LXB4:**  3/4 (CTRL): -14; 3/3 (AD): 112: 3/4 (AD): 337  **5,6 EET:**  3/4 (CTRL): -56; 3/3 (AD): 133: 3/4 (AD): 416  **8,9 EET:**  3/4 (CTRL): -53; 3/3 (AD): 179: 3/4 (AD): 640  **11,12 EET:**  3/4 (CTRL): -34; 3/3 (AD): 14: 3/4 (AD): 91  **14,15 EET:**  3/4 (CTRL): -37; 3/3 (AD): 12: 3/4 (AD): 131  **LTB4:**  3/4 (CTRL): -44; 3/3 (AD): 19: 3/4 (AD): 118  **PGD2:**  3/4 (CTRL): -61; 3/3 (AD): 57: 3/4 (AD): 474  **PGE2a**  3/4 (CTRL): -57; 3/3 (AD): 73: 3/4 (AD): 310  **PGF2a:**  3/4 (CTRL): -27; 3/3 (AD): -71*: 3/4 (AD): -66*  **RvD1/2:**  3/4 (CTRL): -62; 3/3 (AD): 77: 3/4 (AD): 156  **RvD3:**  3/4 (CTRL): -75; 3/3 (AD): 69: 3/4 (AD): 189  **RvD4:**  3/4 (CTRL): -74; 3/3 (AD): 53: 3/4 (AD): 108  **NPD-1:**  3/4 (CTRL): -8; 3/3 (AD): -71*: 3/4 (AD): -56*  **LXA5:**  3/4 (CTRL): -81; 3/3 (AD): 135: 3/4 (AD): 639  **RvE2:**  3/4 (CTRL): -25; 3/3 (AD): 56; 3/4 (AD): 154  **DPA RvD1:**  3/4 (CTRL): -25; 3/3 (AD): 56: 3/4 (AD): 154 |
| **(Fessel et al., 2003)** | National Institute on Aging-Reagan criteria | AD = 5 (2M,3F); CTRL = 5 (3M,2F) | AD = 80.6 ± 10.2; CTRL = 79.0 ± 4.9 | Substantia nigra | AD = 7.8 ± 6.2; CTRL = 6.6 ± 3.7 | Vanderbilt University Medical Center, Duke University Medical Center, and Baylor College of Medicine | Frozen | NA | F2 -IsoPs  GC-MS/NICI | **Substantia nigra:**  F2 -IsoPs: ⇔ | **Substantia nigra:** F2-IsoPs: -26.6 |
| **(Forman et al., 2007)** | National Institute on Aging-Reagan criteria | AD = 10 (4M, 6F); CTRL =10 (8M, 2F) | AD = 88.8 ± 6.7 [82-101]; CTRL = 79.6 ± 6.8 [71-91]; | Neocortex; Entorhinal cortex | AD = 6.1 ± 2.9 [2.8–12.0]; CTRL = 5.3 ± 3.0 [2.3–10.7] | NA | Frozen | NA | 8,12-iso-iPF2a-VI  GC-MS | **Neocortex and entorhinal cortex:**  8,12 Iso-IPF2a-VI**:** ⇑ | **Neocortex and entorhinal cortex:**  **8,12 Iso-IPF2a-VI:** 207.0* (average of brain regions) |
| **(Furman et al., 2018)** | NA | AD = 6 (3M, 3F); CTRL = 3 (M); | AD = [67-86]; CTRL = [68-83] | Frontal cortex | AD = [4-18]; CTRL = [6-14] | Center for Neurodegenerative Disease Research at the University of Pennsylvania | Frozen | NA | **ARA-derived lipid mediators:** 5-HETE;  11-HETE; 12-HETE; 15-HETE; D2/E2 ip; 5 F2 ip;  8 F2 ip; 15 F2 ip;  E2/15 k F2 ip; 15 k E2 ip;  U1; U2; U6;  U10;  U12; U13  MRM-LC/MS/MS | 5-HETE: ⇑;  11-HETE: ⇑;  12-HETE: ⇑;  15-HETE: not detected in the CTRL | 5-HETE: 216.7* 11-HETE: 337.5* 12-HETE: 80* 15-HETE: * D2/E2 ip: 50 5-F2 ip: 44.4 8-F2 ip: -27.8 15-F2 ip: -22.4 E2/15 k F2 ip: 166.7 15 k E2 ip: 400 U1: -37.5 U2: 28.6 U6: -5.6 U10: -45.8 U12: 13529.5 U13: 250* |
| **(Kurano 2022)** | Consortium to Establish a Registry for Alzheimer's Disease (CERAD) score | AD V= 6 (3M,3F); Cerad-b I/II= 7 (2M,5F); CTRL I/II =6 (4M,2F) | AD= 86.17 ± 1.07; Cerad-b= 85.43 ± 3.33; CTRL=83.17 ± 3.34 | Cortex | NA | Brain Bank or Aging Research (Tokyo) | Frozen | NA | 193 eicosanoids/related mediators  LC/MS-MS | 5,6-DiHETE: ⇓  PGE3: ⇓  Resolvin D5: ⇔ 13,14-dihydro-15-keto PGA2: ⇑ 11-trans-LTE4: ⇓  5,6-DHET: ⇓  5,6-DHET-lactone: -50 15-HETE: ⇓ | 5,6-DiHETE: -32.1 PGE3: -97.7 DHA: -38.1* Resolvin D5: 100 13,14-dihydro-15-keto PGA2: 20* 11-trans-LTE4: -68.1* 5,6-DHET: -94.1 5,6-DHET-lactone: -50 15-HETE: -50 |
| **(Lukiw et al., 2005)** | NA | AD = 6 (3M,3F); CTRL = 6 (3M,3F) | AD = 70.3 ± 3.3 [67-76]; CTRL = 69 ± 1.8 [66-71] | Hippocampus; Temporal lobe; Thalamus; Occipital lobe | AD = 2.0 ± 0.6 [1.3-3]; CTRL = 2.1 ± 0.6 [1.3-3] | LSU Brain Tissue Bank, New Orleans, the Canadian Brain Tissue Bank, Toronto, and the Oregon Health Sciences Center Brain Bank | Frozen | NA | NPD1  LC-PDA-ESI-MS-MS–based lipidomic analysis | **Hippocampus:**  NPD1: ⇓;  **Temporal lobe:**  NPD1: ⇓;  **Thalamus:**  NPD1: ⇔;  **Occipital lobe:**  NPD1: ⇔ | **Hippocampus:** -91.6* **Temporal lobe:** -95.2* **Thalamus:** NA **Occipital lobe:** NA |
| **(Nourooz-Zadeh et al., 1999)** | Consortium to Establish a Registry for Alzheimer's Disease (CERAD) score | AD = 10; CTRL = 8 | AD = 79.9 ± 6.1; CTRL = 74.1 ± 16.0 | Occipital lobe; Temporal lobe; Parietal lobe | AD = 29.9 ± 18.2; CTRL = 46.7 ± 12 | Brain Bank, Institute of Psychiatry, Denmark Hill, London, U.K. | Frozen | NA | F2-IsoPs; F4-IsoPs  GC-MS/NICI | ***Occipital lobe:***  **F2-IsoPs:** ⇔;  **F4-IsoPs:** ⇑;  ***Temporal lobe****:*  **F2-IsoPs:** ⇔;  **F4-IsoPs:** ⇑;  ***Parietal lobe:***  **F2-IsoPs:** ⇔;  **F4-IsoPs:** ⇔ | **Occipital lobe:** F2-IsoPs: 21.6; F4-IsoPs (compI): 1000*;  F4-IsoPs (compII): 5900*;  **Teporal lobe:** F2-IsoPs: -2.6; F4-IsoPs (compI): 700*; F4-IsoPs (compII): 900*;  **Parietal lobe:** F2-IsoPs: -1.3; F4-IsoPs (compI): -50; F4-IsoPs (compII): -55.6 |
| **(Pratico et al., 1998)** | National Institute of Aging (Khachaturian, 1985)^#^ | AD = 19 (10M,9F); CTRL = 8 (5M,3F) | AD = 79 ± 2.1 [56-92]; CTRL = 76 ± 4.8 [60-98] | Frontal cortex; Temporal cortex; Cerebellum | AD = 10.3 ± 1.4 [4-17]; CTRL = 11.4 ±1.4 [ 5-16] | NA | Frozen | NA | F2-Isoprostane (iPF2a-III and iPF2a-VI; 6-keto PGF1a)  GC-MS | **Frontal cortex:**  **iPF2a-III:** ⇑;  **iPF2a-VI:** ⇑;  keto PGF1a ⇔  **Temporal cortex:**  **iPF2a-III:** ⇑;  **iPF2a-VI:** ⇑;  **Cerebellum:**  **iPF2a-III:**⇔;  **iPF2a-VI:** ⇔; | **Frontal Cortex:** iPF2a-III: 105*; iPF2a-VI: 106.5*;  6-Keto PGF1a: -26.8; **Temporal cortex:** iPF2a-III: 117.1*; iPF2a-VI: 129.2*; **Cerebellum:** iPF2a-III:  -15.2;  iPF2a-VI: 1.6 |
| **(Pratico et al., 2004)** | National Institute of Aging (Khachaturian, 1985)^#^ | AD = 10 (4M,6F); CTRL = 10 (5M,5F) | AD = 79 ± 3 [56-92]; CTRL = 76 ± 3.4 [60-98] | Mid-frontal cortex; Mid-temporal cortex; Cerebellar cortex | AD = 10 ± 1.3 [4-17]; CTRL = 11 ± 1.1 [5-16] | University of Pennsylvania Alzheimer’s Disease Center | Frozen | NA | 8,12-iso-iPF2-VI; 12-HETE, 15-HETE  GC-MS | **Frontal cortex:**  8,12-iso-iPF2-VI: ⇑;  12 HETE: ⇑;  15 HETE ⇑;  **Temporal cortex:**  8,12-iso-iPF2-VI: ⇑;  12 HETE: ⇑;  15 HETE ⇑;  **Cerebellum:**  8,12-iso-iPF2-VI: ⇔;  12 HETE: ⇔;  15 HETE ⇔ | **Frontal cortex:** 8,12-iso-iPF2-VI **:**77.3*; 12-HETE: 53.8*; 15-HETE: 57.1* **Temporal cortex:** 8,12-iso-iPF2-VI: 67.6*; 12-HETE: 64.1*; 15-HETE: 52.9* **Cerebellum:** 8,12-iso-iPF2-VI : 10.5, 12-HETE:0; 15-HETE: 7 |
| **(Reich et al., 2001)** | neuropathological examination (NINCDS-ADRDA Alzheimer's Criteria, and Consortium to Establish a Registry for Alzheimer's Disease (CERAD) score) | AD = 9 (4M,5F) (61% APOE4); CTRL = 11 (5M,6F) (18% APOE4) | AD = 78.1 ± 2.7; CTRL = 80.7 ± 2.5 | Hippocampus; Superior and temporal gyri;  Inferior parietal lobule; Cerebellar cortex | AD = 2.6 ± 0.2; CTRL = 2.7 ± 0.2 | NA | Frozen | NA | F2- IsoPs; F4-NPs  GC-MS/NICI | **F2- IsoPs:** ⇑;  **F4-NPs:** ⇑  (Not significantly different) | **Superior and middle temporal gyri:** F4-NP2: 168.1; F2-IsoPs: 51.2;  **Hippocampus**: F4-NP2: 164.4; F2-IsoPs: 42.9; **Inferior parietal lobule:** F4-NPs: 144.8 F2-IsoPs: 100; **Cerebellum:** F4-NP2: 106.5 F2-IsoPs: 171.1 |
| **(Wang et al., 2015)** | ICD-10 criteria | AD = 10 (2M,8F); CTRL = 10 (4M,6F) | AD = 80.4 ± 6.3; CTRL =80.2 ± 7.0 | Hipppocampus lateral geniculate nucleus | AD = 21± 12.3; CTRL =17.4 ± 12.1 | Brain Bank at Karolinska Institutet | Half 1: Fixed in formalin; paraffin-embedded tissues; Half 2: frozen (-80 degrees Celsius) | NA | **DHA-derived lipid mediators:** RvD1; Maresin 1; **ARA-derived lipid mediators:** LXA4 ; 5,15-diHETE; LTB4; PGD2; PGE2; PGF2a; TXB2; 5-HETE 12-HETE 15-HETE  LC-MS-MS (lipid mediator profile) (on 3 AD and 3 CTRL)  Enzyme immunoassay  (LX4 and RvD1) | **LXA4:**  not detected in AD by LC-MS-MS **Maresin1:** not detected in AD by LC-MS-MS;  **5,15-diHETE:** ⇓;  **LTB4:** ⇓;  **PGD2:** ⇑; **PGE2:** ⇑; **PGF2a:** ⇑; **TXB2:** ⇑; **5-HETE:** ⇑; **12-HETE:** ⇓; **15-HETE:** ⇑ | **RvD1:** -25  **Maresin 1:** NA **LXA4 (EIA):** -48* **5,15-diHETE:** 69.2 **LTB4:** -46.7 **PGD2:** 261.3 **PGE2:** 115.7 **PGF2a:** 307.6 **TXB2:** 63.8 **5-HETE:** 22.6 **12-HETE:** -12.7 **15-HETE:** 38.6 |
| **(Wong et al., 1992)** | National Institute of Aging (Khachaturian, 1985)^#^ | AD = 10 (4M,6F); CTRL = 10 (4M,6F) | AD = 79.5 ± 1.5; CTRL = 77.2 ± 3.8 | Frontal cortex | AD = 5.6 ± 1.2; CTRL = 5.2 ± 0.6 | NA | Frozen | **AD:** 1 on long-term ASA, 1 on naproxen(for 1 month 4 months before death), 1 on acetaminophen (frequently) and ASA before death; **CTRL:** 2 on long-term ASA, 1 on long-term indomethacin and prednisone, 1 on naproxen (frequently), 1 on acetaminophen immediately before death. | PGA2, PGF2a, PGE2, PGD2, TXB2  Prostanoid production (microsomal fraction)  Assay | ***Frontal cortex:***  **Total prostanoid:** ⇓;  **PGE2:** ⇓;  **PGF2:** ⇓;  **PGD2:** ⇓;  **TXB2:** ⇔ | **Frontal cortex:** Total Prostanoid: -45.8* PGE2: -45.2* PGF2a: -48.6* PGD2: -62.9*  TXB2: 33.3 |
| **(Yao et al., 2003)** | NA | AD = 23 (11M,12F); CTRL = 14 (8M,6F) | AD = 75.7 ± 6 [56-90]; CTRL = 76.5 ± 5 [60-98] | Frontal cortex; Temporal cortex; Occipital cortex, Cerebellar cortex | AD = 9.3 ± 5 [3-17]; CTRL = 13 ± 10 [3.5-30] | NA | Frozen | NA | 8,12-iso-iPF2-VI  GC-MS | **Frontal cortex:**  8,12-iso-iPF2-VI: ⇑  **Temporal cortex:**  8,12-iso-iPF2-VI: ⇑;  **Occipital cortex:**  8,12-iso-iPF2-VI: ⇔;  **Cerebellum:**  8,12-iso-iPF2-VI: ⇔ | **Frontal cortex:** 123.3* **Temporal cortex:** 124.7* **Occipital cortex:** 30 **Cerebellum:** -3.4 |
| **(Zhu et al., 2016)** | Pathological examination | AD = 7 (0M,7F) (Braak stage 5–6/definite AD); CTL = 7 (3M,4F) | AD = 78.1 ± 5.3; CTRL = 21.3 ± 7.5 | Entorhinal cortex | AD = 18.6 ± 8.6; CTL = 21.3 ± 7.5 | Brain Bank at Karolinska Institutet | Frozen | NA | **ARA-derived lipid mediators:** LXA4; LXB4; 5,15-diHETE; LTB4; 20-OH-LTB4; PGD2; PGE2; PGF2α; TxB2; **EPA-derived lipid mediators:** RvE1; RvE2; **DHA-derived lipid mediators:**  RvD1; RvD2; RvD5; Maresin 1; PD1  LC-MS-MS (lipid mediator profile) | **ARA-derived lipid mediators:**  LXA4: ⇔; LXB4: ⇔; 5,15-diHETE: ⇔; LTB4: ⇔; 20-OH-LTB4: ⇔; PGD2: ⇑; PGE2: ⇔; PGF2α: ⇔; TxB2: ⇔; **EPA-derived lipid mediators:** RvE1: ⇔; RvE2: ⇔; **DHA-derived lipid mediators:**  RvD1: ⇔; RvD2: ⇔; RvD5: ⇓;  Maresin 1: not detected in AD; PD1: ⇓ | **ARA-derived lipid mediators:** LXA4: 159.8 LXB4: -55.5 5,15-diHETE: -31.4 LTB4: -33.5 20-OH-LTB4: 32.6 PGD2: 447.7* PGE2: 123.2 PGF2α: -90.2 TxB2: 78.1 **EPA-derived lipid mediators:**  RvE1: 19150.8 RvE2: 318.3 **DHA-derived lipid mediators:** RvD1: 675.9 RvD2: 383.9 RvD5: -48.6* Maresin 1: NA* PD1: -99.9* |

**Abbreviations:** AD, Alzheimer’s disease; CTRL, control subjects; F, female; GC-MS/NICI, gas chromatography-mass spectrometry/ negative ion chemical ionization; h, hours; HETE, Hydroxyeicosatetraenoic acid; IHC, immunohistochemistry; IsoPs, isoprostanes; M, male; MRM-LC/MS/MS, multiple reaction monitoring-liquid chromatography-tandem mass spectrometry; NA, not available; NPD1, neuroprotection D1; PD1, protectin D1; PGA2, prostaglandin A2; PGF2a, prostaglandin F2a; PGE2, prostaglandin E2; PGD2, Prostaglandin D2; RvD, resolvin D; RvE, resolving E; TXB2,thromboxane 2; U, unknown.

* AD and CTRL significantly different (as reported in the publication)

⇓: decreased levels in AD; ⇑: increased levels in AD; ⇔: similar levels between AD and control

Roman numerals refer to Braak stages as reported in original publication

^#^ Khachaturian, Z. S. (1985). Diagnosis of Alzheimer’s Disease. Archives of Neurology, 42(11), 1097–1105. https://doi.org/10.1001/ARCHNEUR.1985.04060100083029

**Table 2. Table reporting COX levels in AD and control brains**

| **Ref.** | **Diagnosis of AD** | **Number of cases (M,F)** | **Age (Years ± SEM) or [age range]** | **Brain region** | **Post-mortem delay (h ± SEM) or [range]** | **Brain Bank** | **Brain fixation** | **Use of anti-inflammatory** | **Enzyme & Methods** | **Localization** | **Expression level in AD** | **% of difference AD vs CTRL** |
| --- | --- | --- | --- | --- | --- | --- | --- | --- | --- | --- | --- | --- |
| **(Chang et al., 1996)** | Gradual progression of dementia in the absence of neurological, psychiatric, or systemic disorders sufficient to cause dementia, as well as neuropathologic evidence satisfying the NIH/NIA age,adjusted criteria for AD | **Neocortex:** AD = 3; CTRL = 5; **Putamen:**  AD = 5; CTRL = 4 (sex not reported) | **Neocortex:** AD = 79 ± 6.4**;** CTRL = 78 ± 6.5 / **Putamen:** AD = 81.4 ± 2.8; CTRL = 72.8 ± 3.7 | **Neocortex:** AD = 6.6 ± 2.2; CTRL = 12.7 ± 2.0 / **Putamen:** AD = 11.4 ± 3.1; CTRL = 13.7 ± 2.0 | Neocortex (Brodmann's areas 12/32); Putamen | Frozen; PFA | Rochester Alzheimer's Disease Center | NA | PGHS-2 (COX2)  Northern blot hybridization; IHC/ISH | Primarily localized in neurons | **Putamen:** COX-2: ⇔;  **Neocortex:** COX-2: ⇓ | **Putamen:** 30.6; **Neocortex**:  -62.8* |
| **(Colangelo et al., 2002)** | CERAD/NIH criteria | AD = 6 (3F,3M); CTRL = 6 (3M,3F) | AD = 70.3 ± 3.3; CTRL = 69 ± 1.8 | AD = 2.0  ± 0.6; CTRL = 2.1 ± 0.6 | Hippocampal cornu ammonis region 1 of hippocampus (CA1) | Frozen | LSU Brain Tissue Bank, New Orleans, the Canadian Brain Tissue Bank, Toronto, and the Oregon Health Sciences Center Brain Bank | NA | COX-2  gene expression | NA | **COX-2 mRNA:** ⇑; | **CA1:** 210* |
| **(Fiala et al., 2002)** | NA | AD = 8; CTRL = 5 (sex not reported) | AD = [68-86]; CTRL = [61-99] | 5-6h | Temporal lobe; Frontal lobe; Hippocampus | Paraffin-embedded tissues | UCLA ADRC Brain Bank | NA | COX-2   IHC | Expression in perivascular macrophages | **COX-2 IR:** ⇑; | Not reported |
| **(Fujimi et al., 2007)** | Guidelines of the National Institute of Neurological and Communicative Disorders and Stroke and the Alzheimer’s Disease and Related Disorders Association | AD = 25 (9M,16F); CTRL = 25 (9M,16F) | AD = > 76 [76-95]; Nondemented = > 76 [77-95] | NA | Hippocampus | **NA** | Formalin-fixed | NA | COX-2  IHC | AD CA1 neurons exhibited increased COX-2 immunoreacticitiy (correlated wiuth AD severity). Neuronal expression of COX-2 in CA3 subdivision of hippocampus, subiculum, entorhinal cortex and transentorhinal cortex observed in AD and nondemented and did not show differences | **COX-2 IR Hippocampus:**  **CA1:** ⇑;  **CA3** ⇔;  **Entorhinal cortex:** ⇔; **Trans-entorhinal cortex:** ⇔;  **Subiculum:**  ⇔; | **COX-2 IR:**  **CA1**: 19.9* **CA3:** 7.8 **Subiculum:** 5.1 **Entorhinal cortex:** 0.3 **Trans-entorhinal cortex:** 0.3 |
| **(Ho et al., 1999)** | Consortium to Establish a Registry for Alzheimer’s Disease (CERAD) criteria | SAD = 12 (5M,7F); CTRL = 11 (7M,5F) | SAD = 83.5 ± 3.3; CTRL = 75.7 ± 4.8 | SAD = 5.4 ± 1; CTRL = 5 ± 0.7 | Hippocampus | Paraffin-embedded tissues | Alzheimer’s Disease Research Center of Mount Sinai School of Medicine | NA | COX-2  IHC | Primarily localized in neurons; not detected in glial cells | **Hippocampus: COX2 IR:**  CA3: ⇑; CA2: ⇑;  CA1: ⇑; C4: ⇑; DG: ⇔ | **Hippocampus:**  CA3: 86.7*; CA2: 103.37*;  CA1: 76.7*;  C4: 78.3*; DG: 18.0 |
| **(Ho et al., 2001)** | Consortium to Establish a Registry for Alzheimer’s Disease (CERAD) criteria | Group classified based on CDR score (dementia): 0 = 8 (88%F); 0.5 = 12 (83%F); 1 = 11 (64%F); 2 = 10 80%F); 5 = 13 (77%F) | 0 = 80 ± 3; 0.5 = 84 ± 3; 1 = 87 ± 3; 2 = 88 ± 2; 5 = 86 ± 3 | 0 = 9.7 ± 5.7; 0.5 = 13.8 ± 6.9; 1 = 13 ± 6.5; 2 = 6.8 ± 3.5; 5 = 7.5 ±2.7 | Hippocampus | Paraffin-embedded tissues | Alzheimer’s Disease Research Center of Mount Sinai School of Medicine | NA | COX-2  IHC | **NA** | **Hippocampus: COX2 IR:**  **CA3:**  0.5: ⇔;  1: ⇑;  2: ⇑;  5: ⇑; **CA2:**  0.5: ⇔;  1: ⇑;  2: ⇑;  5: ⇑;   CA1:  0.5:  1: ⇔;  2: ⇔;  5: ⇑ | **Hippocampus CA3:** 0.5: 16.5; 1: 34.0*; 2: 50.5*; 5: 68.0*;  **Hippocampus CA2:** 0.5: 9.7; 1: 38.8*; 2: 47.6*; 5: 66.0*;  **Hippocampus CA1:** 0.5: -17.4; 1: 2.9; 2: 7.8; 5: 64.1*; |
| **(Hoozemans et al., 2001)** | neuropathologically confirmed on formalin-fixed, paraffin-embedded tissue from different sites | AD = 6 (6F); CTRL = 3 (3M); | AD = [76-92]; CTRL = [63-81] | Not reported | Temporal cortex; Frontal cortex | Formalin-fixed  paraffin-embedded tissue | The Netherlands Brain Bank | NA | COX-1, COX-2 IHC | COX-1: expressed in neuron and microglia cells in white and grey matter; not expressed in astrocytes and endothelial cells; COX-2: expressed in neurons and microglia; not expressed in astrocytes and endothelial cells | Temporal cortex:  COX-2 IR: ⇑; COX-1 IR: NA | Temporal cortex:  COX-2 IR: 163.9*;  COX-1 IR: NA |
| **(Hoozemans et al., 2002)** | neuropathologically confirmed on formalin-fixed, paraffin-embedded tissue from different sites | AD = 10 (3M,7F); CTRL = 17 (7M,10F)/ Braak staging:  O = 4 (3M,1F); A = 3 (2M,1F); B =10 (3M,7F); C = 10 (2M,8F) | AD = [65-91]; CTRL = [53-97]; Braak staging: O = [53-78]; A = [78-82]; B = [67-92]; C = [65-91] | 7h | Temporal cortex | Formalin-fixed paraffin-embedded tissue | The Netherlands Brain Bank | NA | COX-2  IHC | Expression in neuronal cells; most prominent in layer III of cortex | **Temporal cortex:**  **COX-2 IR:** ⇑; | **Temporal cortex:**  A: 110.7*;  B: -10.7;  C: -53.6 |
| **(Hoozemans et al., 2004)** | DSM-III-R criteria and the severity of dementia was evaluated according to the Global Deterioration Scale of Reisberg (GDS) | AD = 19 (3M, 16F); CTRL = 21 (9M, 12F) **Braak staging for amyloid deposits:** O = 7 (4M; 3F); A = 6 (3M, 3F); B = 11 (3M; 8F); C = 16 (2M; 14F) ^^ | **Braak staging for amyloid deposits**: O = 69 ± 12; A = 79 ± 4; B = 85 ± 10; C= 82 ± 10 | **Braak staging for amyloid deposits:** O = 10.4 ± 6.8; A = 7.8 ± 2.9; B = 6.6 ± 2.3; C = 5.7 ± 1.7 | Mid-temporal cortex | Formalin-fixed paraffin-embedded | The Netherlands Brain Bank | NA | COX-2  IHC | Localized in the cytoplasm, dendrites, and axons of pyramidal neurons | **Mid- Temporal cortex:**  **COX-2 IR:**  A: ⇔;  B: ⇔;  C: ⇑ | **Mid- Temporal cortex:**  **COX-2 IR:**  A: 35.9 B: -44.1 C: -82.3* |
| **(Hoozemans et al., 2005)** | NA | **Braak staging for amyloid deposits:** O = 7 (4M; 3F); A = 6 (3M, 3F); B = 11 (3M; 8F); C = 16 (2M; 14F)  ^^ | **Braak staging for amyloid deposits:** O = 69 ± 12; A = 79 ± 4; B= 85 ± 10; C= 82 ± 10 | **Braak staging for amyloid deposits:** O = 7 ± 4; A =8 .3 ± 3; B - 7 ± 2.3; C = 6 ± 2 | Temporal cortex | Formalin-fixed paraffin-embedded | The Netherlands Brain Bank | NA | COX-2  IHC | Pyramidal neurons | **Temporal cortex:**  **COX-2 IR:**  A: ⇑;  B: ⇔;  C: ⇔ | **Temporal cortex:**  **COX-2 IR:**  A: 30.6* B: -59.9 C: -71.4 |
| **(Kitamura et al., 1999)** | Consortium to Establish a Registry for Alzheimer’s Disease (CERAD) criteria | AD = 7; CTRL =6 | AD = [70-94]; CTRL = [60-87] | AD = [2-7]; CTRL = [2-21] | Temporal cortex | Frozen | NA | NA | COX-1;  COX-2  WB | NA | Temporal cortex:  COX-2 protein: ⇑;  COX-1 protein: ⇑ | Temporal cortex:  COX-2  protein: 75.4* COX-1 protein: 38.5* |
| **(Lukiw and Bazan, 1997)** | Consortium to Establish a Registry for Alzheimer’s Disease (CERAD) | SAD = 10 (6M,4F); CTRL = 15 (6F,9M) | SAD = 69.4 ± 6; CTRL = 68.6 ± 5.4 | SAD = 6.1 ± 3.9; CTRL = 5.4 ±4.7 | Neocortex | Frozen | LSU Neuroscience Center Brain Bank, New Orleans | NA | COX-1;  COX-2  Gene expression | NA | Neocortex:  COX-1: ⇔;  COX-2: ⇔ (hypervariability) | Neocortex:  COX-1: 15.4;  COX-2: 25 |
| **(Lukiw and Bazan, 1998)** | Consortium to Establish a Registry for Alzheimer’s Disease (CERAD) | AD = 8 (4F,4M); CTRL = 12 (5F 7M) | AD = 69.6 ± 6.3; CTRL = 69 ± 5.27 | AD = 3.9 ± 1.8; CTRL = 3.28 ± 1.74 | Superior temporal gyrus (Brodmann area A22) | Frozen | LSU Neuroscience Center Brain Bank, New Orleans | Not reported | COX-2  Gene expression and nuclear protein extract | NA | **Superior temporal gyrus:**  **COX-2:** ⇔ (hypervariability) | Not reported |
| **(Mohri et al., 2007)** | 1) clinical dementia rating of ≥1 (10); 2) the topographical distribution of senile plaques matching Braak stage C; and 3) of neurofibrillary tangles equal to or above stage IV | AD = 17 (10M,7F); CTRL = 12 (9M,3F) | AD = 83. 6 [70-93]; CTRL = 79.5 [71-91] | AD = 8.5 [1.5-17]; CTRL = 12.1 [2.3-14.8] | Frontal cortex | Tokyo Metropolitan Brain Bank for Aging Research | Frozen; PFA | NA | COX-1; COX-2  RT-PCR | NA | Frontal cortex:  COX-1 mRNA:  ⇔;  Cox-2 mRNA: ⇔ | COX-1 mRNA: 6.1 COX-2 mRNA: -9.1 |
| **(Pasinetti and Aisen, 1998)** | Consortium to Establish a Registry for Alzheimer’s Disease (CERAD) | AD = 9; CTRL = 9 | AD = 77 ± 3; CTRL = 82.3 ± 3 | AD = 6 ± 2; CTRL = 5.1 ± 2 | Frontal cortex (Brodmann area 6) | NA | NA | NA | COX-1;  COX-2  Northern blot; WB; IHC | Primarily localized in neurons | Frontal cortex:  COX-2 mRNA: ⇑; COX-1 mRNA: ⇔; COX-2 protein: ⇑;  COX-2 IR: ⇑ | Frontal cortex:  COX-2 mRNA: 35.1*;  COX-1 mRNA: -11.1 COX-2 protein: 79.1* |
| **(Yasojima et al., 1999)** | Clinical history and standard neuropathological findings | AD = 5 (1M,4F); CTRL = 5 (1M,4F) | AD = [65-78]; CTRL = [43-82] | AD = [6-16]; CTRL = [9-48] | Entorhinal cortex; Hippocampus; Midtemporal gyrus; Midfrontal cortex; Amygdala; Substantia nigra; Thalamus; Occipital cortex; Motor, cortex; Caudate; Cerebellum | NA | Frozen | NA | COX-1;  COX-2  qPCR; IHC | localizaiton in neuronal perikarya and dendrites | Entorhinal cortex:  COX-1 mRNA:  ⇔;  Cox-2 mRNA: ⇑;  Hippocampus:  COX-1 mRNA:  ⇔;  Cox-2 mRNA: ⇑;  Mid-temporal gyrus:  COX-1 mRNA:  ⇔;  Cox-2 mRNA: ⇑;  Midfrontal cortex:  COX-1 mRNA:  ⇔;  Cox-2 mRNA: ⇔;  Amygdala: COX-1 mRNA:  ⇔;  Cox-2 mRNA: ⇔;  Substantia nigra:  COX-1 mRNA:  ⇔;  Cox-2 mRNA: ⇑;  Thalamus: COX-1 mRNA:  ⇔;  Cox-2 mRNA: ⇑;  Occipital cortex:  COX-1 mRNA:  ⇔;  Cox-2 mRNA: ⇔;  Motor, cortex: COX-1 mRNA:  ⇔;  Cox-2 mRNA: ⇔;  Caudate:  COX-1 mRNA:  ⇔; Cox-2 mRNA: ⇔;  Cerebellum: COX-1 mRNA:  ⇔; Cox-2 mRNA: ⇔ | Entorhinal cortex:  COX-1 mRNA:  54.3;  COX-2 mRNA:   150.7*;  Hippocampus:  COX-1 mRNA: 78.4;  COX-2 mRNA: 113.7*;  Mid-temporal gyrus:  COX-1 mRNA: 82.0;  COX-2 mRNA: 104.2*;  Midfrontal cortex:  COX-1 mRNA: 51.1;  COX-2 mRNA: 79.0;  Amygdala: COX-1 mRNA: 45.2;  COX-2 mRNA: 40;  Substantia nigra:  COX-1 mRNA: 0;  COX-2 mRNA: 103.0*;  Thalamus: COX-1 mRNA: 74.4;  COX-2 mRNA: 41.4*;  Occipital cortex:  COX-1 mRNA: 48.8;  COX-2 mRNA: 11.1;  Motor, cortex: COX-1 mRNA:  21.0;  COX-2 mRNA: 29.1;  Caudate: COX-1 mRNA: 28.2;  COX-2 mRNA: 1.1;  Cerebellum: COX-1 mRNA:  60.5 COX-2 mRNA: 1.4 |
| **(Yermakova and O'Banion, 2001)** | NA | AD = 20 (6M,14F); CTRL = 7 (6M,1F) | AD = [64-92]; CTRL = [63-93] | AD = [2-18.8]; CTRL = [6.3-11.92] | Posterior hippocampus;  Temporal cortex; Motor cortex | Gluteraldehyde; Frozen | University of Rochester Alzheimer’s Disease Center Brain Bank | NA | COX-2  WB; IHC | Expressed in neurons astrocytes and blood vessels | **Hippocampus: COX-2 protein:**  ⇔;  **COX-2 IR Hippocampus:**  **Hilus:** ⇔;  CA3: ⇓;  CA2: ⇓;  **CA1:** ⇔;  **Temporal lobe:**  **Astrocytes IR:**  ⇑ | **Hippocampus: COX-2 protein:**  -48.4;  **COX-2 IR Hippocampus:**  Hilus: -29.4;  CA3: -16.23*; CA2: -18.6; CA1: -22.1*;  **Temporal lobe:**  **Astrocytes IR:**  IR: 137.6* |
| **(Yermakova et al., 1999)** | NA | AD = 10; CTRL = 10 (sex not reported) | AD = 81.2 [76-90]; CTRL = 80.7 [74-92] | AD = 2.6 [1.5-4.5]; CTRL = 2 [1.66-2.75] | Hippocampus;  Cortex | PFA | Sun Health Resaerch Institute and University of Rochester Alzheimer's Disease center brain banks | NA | COX-1  ISH, IHC | Hippocampus: Expression in CA3 and CA4 scattered in CA2 and no expression in CA1 and DG;  Cortex: expression in neurons;  Expression in neuron and microglia. No expression in astrocytes | Hippocampus: COX1 mRNA:  CA3 neurons: ⇔;  Cortex:  COX-1 density in microglia:  Layer I: ⇔;  Layer II: ⇑;  Layer III: ⇑;  White matter: ⇔; | Hippocampus: COX1 mRNA:  CA3: -13.5  Cortex:  COX-1 density in microglia:  Layer I: 10.7  Layer II: 37.6*  Layer III: 31.2*  white matter: 10.2 |
| **(Yokota et al., 2003)** | The consortium to establish a registry for Alzheimer’s disease (CERAD) | AD = 17 (9M,8F); CTRL = 26 (13M,13F) | AD = 72.4 + 11.7; CTRL = 68.8 + 10 | AD = 3.3 + 1.2; CTRL = 2.9 + 0.9 | Hippocampus | NA | Formalin-fixed embedded | NA | COX-2  s  IHC | Neuronal perikaryons | **COX-2 IR:** ⇑; **COX-2 density:** ⇓ | **COX-2 IR:**  CA1: 86.3*; CA2: 92.2*; CA3: 64.9*; CA4: 82.2*;  **COX-2 density:** CA1: -42.8*; CA2: -31.5*; CA3: -35.0*; CA4: -35.7* |
| **(Yokota et al., 2004)** | The consortium to establish a registry for Alzheimer’s disease (CERAD) | AD = 17 (7M,10F); CTRL = 22 (13M,9F) | AD = 73.9 + 11.7; CTRL = 71.2 + 11 | AD = 5.3 + 2.6; CTRL = 5.7 + 2.6 | Hippocampus | Okayama University Graduate School of Medicine and Dentistry, Okayama, and the Tokyo Metropolitan Institute of Gerontology, Tokyo | Formalin-fixed paraffin-embedded tissue blocks of | NA | COX-2 IHC | Neuronal perikaryons | **COX-2 IR:** ⇑; | **COX-2 IR:**  CA1: 101.4*; CA2: 105.1*; CA3: 68.8*; CA4: 84.7* |

**Abbreviations:** AD, Alzheimer’s disease; CDR, clinical dementia rating; CTRL, control subjects; F, female; h, hours; IHC, immunohistochemistry; IR, immunoreactivity; ISH, *in situ* hybridization; M, male; NA, not available; PFA, paraformaldehyde; SAD, Sporadic AD; WB, western blot.

* AD and CTRL significantly different (as reported in the publication)

^^ Braak staging for neurofibrillary tangles also reported but did not show any differences between groups.

⇓: decreased levels in AD; ⇑: increased levels in AD; ⇔: similar levels between AD and control

Not reported: Study does not report values for AD and control groups.Layer I, II, and III of the cortex

**Table 3. Table reporting the levels of lipoxygenases in AD and control brains.**

| **Ref.** | **Diagnosis of AD** | **Number of cases (M,F)** | **Age (Years ± SEM) or [age range]** | **Brain region** | **Post-mortem delay (h ± SEM) or [range]** | **Brain Bank** | **Brain fixation** | **Use of anti-inflammatory** | **Enzyme & Methods** | **Localization** | **Expression level in AD** | **% of difference AD vs CTRL** |
| --- | --- | --- | --- | --- | --- | --- | --- | --- | --- | --- | --- | --- |
| **(Firuzi et al., 2008)** | National Institute of Aging (Khachaturian, 1985) | AD = 5 (2M,3F); CTRL = 2 (1M,1F) | AD = 78.4 ± 2.2; CTRL = 72.0 ± 7.0 | Hippocampus; Cortex | NA | NA | NA | NA | 5-LOX  WB | NA | **Hippocampus**: ⇑;  **Cortex:** ⇑ | **Hippocampus:** 403.0* **Cortex:** 40.4* |
| **(Ikonomovic et al., 2008)** | All had clinical diagnoses of AD and autopsy-confirmed AD with advanced stages of neuropathology (Braak V/VI) | AD = 11 (4M,7F); CTRL = 5 (3M,2F) | AD = [60-93]; CTRL = [49-75] | Medial temporal lobe (hippocampus, parahippocampal gyrus, and inferior temporal cortex) | AD = [4-14]; CTRL = [10-20] | University of Pittsburgh Alzheimer’s Disease Research Center (ADRC) | PFA | NA | 5-LOX  IHC (semi quantitative) | Association with neurofibrillary structures and amyloid plaques | **Hippocampus:** ⇑ | Not reported |
| **(Lukiw et al., 2005)** | NA | AD = 6 (3M, 3F); CTRL = 6 (3M, 3F) | AD = 70.3 ± 3.3 [67-76]; CTRL = 69 ± 1.8 [66-71] | Hippocampal cornu ammonis region 1 of hippocampus (CA1) | AD = 2.0 ± 0.6 [1.3-3]; CTRL = 2.1 ± 0.6 [1.3-3] | LSU Brain Tissue Bank, New Orleans, the Canadian Brain Tissue Bank, Toronto, and the Oregon Health Sciences Center Brain Bank | Frozen | NA | 15-LOX  Gene expression (Affymetrix) | NA | **Hippocampus:** ⇓ | **Hippocampus**: -194.7* |
| **(Pratico et al., 2004)** | National Institute of Aging (Khachaturian, 1985)^#^ | AD = 10 (4M,6F); CTRL = 10 (5M,5F) | AD = 79 ± 3 [56-92]; CTRL = 76 ± 3.4 [60-98] | Mid-frontal cortex; Mid-temporal cortex; Cerebellum | AD = 10 ± 1.3 [4-17]; CTRL = 11 ± 1.1 [5-16] | University of Pennsylvania Alzheimer’s Disease Center | Frozen | NA | 15-LOX-1 (12/15-LOX)  WB; IHC | Highest intensity in hippocampus and entorhinal cortex | **Mid-frontal cortex and mid-temporal cortex:** ⇑  **Cerebellum:** ⇔ | **Frontal cortex:** 60.2* **Temporal cortex:** 55.3* **Cerebellum**:  -14.8 |
| **(Wang et al., 2015)** | ICD-10 criteria | AD = 10 (2M,8F); CTRL = 10 (4M,6F) | AD = 80.4± 6.275; ctrl =80.2 ± 7.021 | Hipppocampus lateral geniculate nucleus | AD = 21± 12.266; CTRL =17.4 ± 12.1; | Brain Bank at Karolinska Institutet | Half 1: fixed in formalin; paraffin embedded tissues; Half 2: frozen | NA | 15-LOX-2  WB; IHC | Labeling in glial cells (microglia and astrocytes), not in neurons. Predominant staining in CA2–4 and the subgranular zone of DG, fewer immunopositive cells in the CA1 | **Hippocampus (glial cells):** ⇑ | **Hippocampus (glial cells):** 154.2* |

**Abbreviations:** AD, Alzheimer’s disease; CTRL, control subjects; F, female; h, hours; IHC, immunohistochemistry; M, male; NA, not available; PFA, paraformaldehyde; WB, western blot.

* AD and CTRL significantly different (as reported in the publication)

⇓: decreased levels in AD; ⇑: increased levels in AD; ⇔: similar levels between AD and control

Not reported: Study does not report values for AD and control groups.

^#^ Khachaturian, Z. S. (1985). Diagnosis of Alzheimer’s Disease. Archives of Neurology, 42(11), 1097–1105. https://doi.org/10.1001/ARCHNEUR.1985.04060100083029

**Table 4. Table reporting the levels of prostaglandin synthase in AD and control brains.**

| **REF.** | **Diagnosis of AD** | **Number of cases (M,F)** | **Age (Years ± SEM) or [age range]** | **Post-mortem delay (h)** | **Brain region** | **Brain fixation** | **Brain bank** | **Use of anti-inflammatory** | **Enzymes & Methods** | **Localization** | **Expression level in AD** | **% of difference AD vs CTRL** |
| --- | --- | --- | --- | --- | --- | --- | --- | --- | --- | --- | --- | --- |
| **(Akitake et al., 2013)** | neuropathological evidence of the loss of temporoparietal neurons and by the presence of SPs and NFTs | AD = 9 (3M, 6F); non-AD Parkinson’s disease and cerebrovascular disorders) = 5 (2M, 3F); CTRL = 4 (1M, 3F) | AD = 85.8 ± 9.5 [68-98]; non-AD = 78.6 ± 8.2 [68-90]; CTRL = 89.3 ± 4.6 [83-94] | NA | Frontal cortex; Parietal cortex; Hippocampus | Formalin fixed and paraffin- embedded | Choju Medical Institute Brain Bank of Fukushimura Hospital (Toyohashi, Aichi, Japan) | NA | mPGES-1; mPGES-2; c-PGES | **mPGES-1:** 18 subjects: localized in neurites and cytoplasm of neurons in CA 2-3; CA1 and CA4 less stained. DG devoid of staining. AD: association with dystrophic neurites surrounding plaques. Not expressed in astrocytes. Not expressed in white matter in cortex. **mPGES-2:** staining in hippocampus. Positive staining in astrocytes. **cPGES:** only dispersed and obscure signal in tissue | **mPGES-1:** ⇑;  **mPGES-2:** ⇔;  **cPGES:** ⇔ | **Hippocampus** CA2-3: mPGES-1: 26.4*; mPGES-2: 8.5 cPGES: 18.9  (values not available for other brain regions) |
| **(Chaudhry and Dore, 2009)** | The Consortium to Establish a Registry of Alzheimer's Disease (CERAD) criteria | SAD = 5 (1M, 4F); FAD = 5 (2M, 3F,); CTRL = 5 (2M,3F) | SAD = [62-89]; FAD = [63-94]; CTL = [68-91] | SAD= [6-19]; FAD = [4-9]; CTRL = [4-16] | Middle frontal gyrus | Frozen | Johns Hopkins Brain Resource center | NA | cPGES  IF | Co-localization with neurons, microglia, and endothelial cells, but not astrocytes or smooth muscle cells. FAD and SAD: Staining barely detectable in neurons | **Middle frontal gyrus:** cPGES: ⇓ | Not reported |
| **(Chaudhry et al., 2008)** | The Consortium to Establish a Registry of Alzheimer's Disease (CERAD) criteria | SAD = 10 (3M, 7F); FAD= 5 (2M, 3F); CTRL = 9 (5M,4F) | SAD = [54-89]; FAD = [63-94]; CTRL = [68-91] | SAD = [3.5-19]; FAD = [4-9]; CTRL = [4-22] | Middle frontal gyrus | PFA; Frozen | Johns Hopkins Brain Resource center | NA | mPGES-1    IHC (5 SAD, 5 FAD, 5 CTRL); WB (10 SAD, 5 FAD, 9 CTRL) | CTRL: Staining negligible; SAD and FAD: positive staining in large pyramidal neurons, microglia and endothelial cells and astrocytes but not smooth muscle | **Middle frontal gyrus:** mPGES-1: ⇑ | **Middle frontal gyrus:** mPGES-1: 50.7* |
| **(Chaudhry et al., 2010)** | The Consortium to Establish a Registry of Alzheimer's Disease (CERAD) criteria | SAD = 10 (3M, 7F); FAD = 5 (2M, 3F); CTRL = 9 (5M, 3F, 1 NA) | SAD = [62-89]; FAD = [63-94]; CTRL= [68-91] | SAD = [3.5-19]; FAD = [4-9]; CTRL = [4-22] | Middle frontal gyrus | Formalin fixed and paraffin-embedded | Johns Hopkins Brain Resource center | NA | mPGES-2  IF; IHC; WB | CTRL: co-localization with activated microglial cells (amoeboid-shaped) cells; resting (ramified-shaped) microglia were not stained. Co-localization with neurons and endothelial cells but not with astrocytes or smooth muscle cells. | **Middle frontal gyrus:** mPGES-2: ⇔ | mPGES-2: 3.8 |
| **(Mohri et al., 2007)** | 1) clinical dementia rating of ≥1 (10); 2) the topographical distribution of senile plaques matching Braak stage C; and 3) of neurofibrillary tangles equal to or above stage IV | AD = 17 (10M, 7F; ); CTRL = 12 (9M, 3F ) | AD = 83. 6 [70-93]; CTRL = 79.5 [71-91] | AD = 8.5 [1.5-17]; CTRL = 12.1 [2.3-17.4 ] | Frontal cortex | PFA; Frozen | Tokyo Metropolitan Brain Bank for Aging Research | NA | mPGES-1; mPGES-2; c-PGES  RT-PCR; IHC | mPGES localized in some neurons | **Frontal cortex:** mPGES-1: ⇔; mPGES-2; ⇔;  cPGES: ⇔ | **Frontal cortex:** mPGES-1: -12.28;  mPGES-2: 5.5; cPGES: 1 |

**Abbreviations:** AD, Alzheimer’s disease; CTRL, control subjects; F, female; h, hours; IHC, immunohistochemistry; M, male; NA, not available; PFA, paraformaldehyde; WB, western blot.

* AD and CTRL significantly different (as reported in the publication)

# SAD and FAD analyzed together by the authors of the publications.

⇓: decreased levels in AD; ⇑: increased levels in AD; ⇔: similar levels between AD and control

Not reported: Study does not report values for AD and control groups.

**Table 5. Table reporting the levels of Phospholipase A2 in AD and control brains.**

| **REF.** | **Diagnosis of AD** | **Number of cases (M,F)** | **Age (Years ± SEM) or [age range]** | **Post-mortem delay (h)** | **Brain region** | **Brain fixation** | **Brain bank** | **Use of anti-inflammatory** | **Enzymes & Methods** | **Localization** | **Expression level in AD** | **% of difference AD vs CTRL** |
| --- | --- | --- | --- | --- | --- | --- | --- | --- | --- | --- | --- | --- |
| **(Colangelo et al., 2002)** | CERAD/NIH criteria | AD = 6 (3M, 3F); CTRL = 6 (3M,3F) | AD = 70.3 ± 3.3; CTRL = 69 ±1.8 | AD = 2.0 ± 0.6; CTRL = 2.1 ± 0.6 | Hippocampal cornu ammonis region 1 of hippocampus (CA1) | Frozen | LSU Brain Tissue Bank, New Orleans, the Canadian Brain Tissue Bank, Toronto, and the Oregon Health Sciences Center Brain Bank | NA | cPLA2  DNA microarray | NA | **Hippocampus:**  cPLA2: ⇑ | **Hippocampus:** cPLA2: 350* |
| **(Doody et al., 2015)** | National Institute of Neurological and Communicative Disorders and Stroke-Alzheimer's Disease and Related Disorders Association (NINCDS-ADRDA) criteria | AD = 10 (1M;9F); CTRL =10 (4M;6F) | AD = 79.2; CTRL= 72.5 | NA | Frontal gyrus; Parietal gyrus; Occipital gyrus; Cingulate gyrus; Hippocampus; Brainstem; Cerebellum; Basal ganglia; Amygdala; Thalamus; Pituitary gland | Formalin-fixed, paraffin-embedded | Department of Pathology of the Methodist Hospital in Houston | NA | Lp-PLA2  IHC | **Lp-PLA2**: not detected | NA | Not reported |
| **(Gattaz et al., 1995)** | Neuropathologically confirmed | AD = 23 (7M,16F); CTRL = 20 (10M,10F) | AD = 81 ± 7.5; CTRL = 75.6 ± 9.8 | AD = 30.7 ± 14.5; CTRL = 38.1 ± 11.1 | Parietal cortex (Brodman area 7); Frontal cortex (Brodman area 32) | NA | Medical Research Council Alzheimer's Disease Brain Bank, Institute of Psychiatry | NA | PLA-2  Calcium- dependent enzymatic activity | NA | **Parietal cortex:** PLA2A: ⇓; **Frontal cortex:** PLA2A: ⇓ | **Parietal cortex**: -36.9* **Frontal cortex:** -29.0* |
| **(Kanfer et al., 1993)** | NA | AD =18; CTRL = 11 (ctrl included non-AD demented controls included patients with Parkinson's disease and dementia, amyotrophic lateral sclerosis and dementia, and multi-infarct dementia) | AD= 70 ± 5.6; non-AD = 69.5 ± 14.5 | AD = 5.7 ± 4.2; non-AD = 7.2 ± 3.3 | Junction of the superior and middle frontal gyri; Superior temporal gyrus | NA | Neuropathology Core of the NIA-funded University of Pittsburgh Alzheimer's Disease Research Center | NA | PLA2  Enzymatic activity | NA | **PLA 2:** ⇔ | **Temporal gyrus:** 14.3 |
| **(Moses et al., 2006)** | Consortium to Establish a Registry for AD (CERAD) and NIA-Reagan guidelines | AD = 16 (5M,11F); CTRL = 16 (7M,9F) | AD = 86.25 ± 8.22; CTRL = 84.44 ± 6.74 | AD = 2.59 ± 0.45; CTRL = 2.63 ± 0.62 | Hippocampus; Inferior Temporal Gyrus; Cerebellum | PFA | Brain Bank of the Sun Health Research Institute | NA | sPLA2-IIA  IHC; qPCR | Expressed in astrocytes, not expressed in microglia | **Hippocampus:** sPLA2: ⇑; **Inferior Temporal Gyrus**: ⇑;  **Cerebellum:** sPLA2:⇔ | **Hippocampus DG:** 95.1*; **Hippocampus CA3:** 100* **Inferior temporal gyri:** 73.2*;  **Cerebellum:** NA |
| **(Ross et al., 1998)** | The presence of both neuritic plaques and neurofibrillary tangles in hippocampus and neocortex in the absence of other degenerative processes | AD =10 (5M,5F); CTRL = 10 (5M,5F) | AD = 75.3 + 3; CTRL = 73 + 2 | AD = 10 + 2; CTRL = 10 + 1 | Parietal cortex (Brodmann area 7b); Temporal cortex (Brodmann area 21); Occipital cortex (Brodmann area 17); Hippocampus (Ammon's horn); Cerebellum | Frozen | NA | NA | PLA2  Calcium-dependent and calcium-independent enzymatic activity | NA | **Parietal cortex**:  PLA2 Ca-dep: ⇓;  PLA2 Ca-indep: ⇓;  **Temporal cortex:**  PLA2 Ca-dep: ⇓;  PLA2 Ca-indep: ⇓;  **Occipital cortex**:  PLA2 Ca-dep: ⇓;  PLA2 Ca-indep: ⇔;;  **Hippocampus:**  PLA2 Ca-dep: ⇓;  PLA2 Ca-indep: ⇔;  **Cerebellum:** PLA2 Ca-dep: ⇔;  PLA2 Ca-indep: ⇔; | **Parietal cortex:**  PLA2 Ca-dep:  -40.5*;  PLA2 Ca-indep: -35.8*  **Temporal cortex:**  PLA2 Ca-dep: -37.0*; PLA2 Ca-indep: -42.8*;  **Occipital cortex**:  PLA2 Ca-dep:  -32.2*; PLA2 Ca-indep: -19.9  **Hippocampus:** PLA2 Ca-dep:: -16.9*;  PLA2 Ca-indep: -23.3  **Cerebellum:** PLA2 Ca-dep: -5.1; PLA2 Ca-indep: 19.7 |
| **(Sanchez-Mejia et al., 2008)** | NA | AD = 12; CTRL = 6 (sex not reported) | NA | Hippocampus | NA | NA | UCSF | NA | PLA-IV A WB | NA | **Hippocampus:** PLA2: ⇑; | **Hippocampus**:  PLA2A: 189.1 * |
| **(Stephenson et al., 1996)** | National Institute of Aging (Khachaturian, 1985)^#^ | AD = 5 (1M,5F); CTRL = 6 (5M, 1F) | AD = [77-90]; CTRL = [36-80] | AD = [5-31]; CTRL = [3-24] | Occipital cortex; Cerebellum | Paraffin-embedded tissues | Brigham and Women’s Hospital | Not reported | cPLA2  IHC | Expression in astrocytes but not in neurons, microglia or endothelial cells. | **Occipital**  **Cortex:**  **cPLA2:⇑**  **Cerebellum:** ⇔ | **Occipital cortex:** 48.5*; Cerebellum: NA |
| **(Talbot et al., 2000)** | National Institute of Aging (Khachaturian, 1985)^#^ and CERAD and displayed densities of neurofibrillary tangles in the middle frontal gyrus (i.e., dorsolateral prefrontal cortex) as high or higher than in the entorhinal cortex of the same brain | AD = 12 (3M,9F); CTRL = 12 (4M,8F) | AD = [61-82]; CTRL = [60-98] | AD = [4-18]; CTRL = [5-22] | Dorsolateral cortex; Prefrontal cortex; Lateral temporal cortex | Frozen | Hospital of the University of Pennsylvania | NA | PLA2  Calcium-dependent and calcium-independent enzymatic activity | NA | **Dorsolateral cortex**:  PLA2 Ca-dep: ⇓;  PLA2 Ca-indep: ⇔;  **Prefrontal cortex:**  PLA2 Ca-dep: ⇔;  PLA2 Ca-indep: ⇔; **Lateral temporal cortex:**  PLA2 Ca-dep: ⇔;  PLA2 Ca-indep: ⇔; | **Frontal cortex:**  PLA2 Ca-dep: -41.5*;  PLA2 Ca-indep: -37.6;  **Temporal cortex**:  PLA2 Ca-dep:  -18.2;  PLA2 Ca-indep: -11.1 |

**Abbreviations:** AD, Alzheimer’s disease; cPLA2, cytosolic phospholipase 2; CTRL, control subjects; F, female; h, hours; IHC, immunohistochemistry; M, male; NA, not available; NFT, neurofibrillary tangles; PFA, paraformaldehyde; sPLA2a, Secretory phospholipase A2-IIA; WB, western blot.

* AD and CTRL significantly different (as reported in the publication)

⇓: decreased levels in AD; ⇑: increased levels in AD; ⇔: similar levels between AD and control

Not reported: Study does not report values for AD and control groups.

^#^ Khachaturian, Z. S. (1985). Diagnosis of Alzheimer’s Disease. Archives of Neurology, 42(11), 1097–1105. https://doi.org/10.1001/ARCHNEUR.1985.04060100083029
